# Supplementary material for: Map-Based Functional Analysis of the GhNLP Genes Reveals Their Roles in Enhancing Tolerance to N-Deficiency in Cotton
Source: Int J Mol Sci. 2019 Oct 8;20(19):4953. doi: 10.3390/ijms20194953 (PMC6801916; doi:10.3390/ijms20194953)
Supplement: Supplementary file 1 [file ijms-20-04953-s001.zip › Table S1.pdf]

Table S1: Physiochemical properties of the NLP proteins in AD, A and D cotton genomes

A. Physiochemical properties of the proteins encoded by the *NLP* genes in *G. hirsutum*

Gene ID	Domain type	Chro.	Start	End	Strand	Length (bp)	Transcript Length (bp)	CDS Length (bp)	CDS GC Content (%)	Exon Number	Mean Exon Length (bp)	Mean Intron Length (bp)	Protein Length (aa)	Molecular Weight (kDa)	Charge	Isoelectric Point	GRAVY	Subcellular localization
Gh_A01G0750	PB1	A01	14,376,117	14,378,141	-	2,025	2,025	2,025	45.8	1	2,025.00	No intron	674	74.404	-20	4.835	-0.496	nucl
Gh_A01G0794	PB1	A01	17,401,444	17,409,421	+	7,978	3,732	3,732	41.5	7	533.1	707.7	1,243	138.311	-23	5.351	NA	E.R.
Gh_A01G0844	NLP4	A01	19,416,521	19,419,606	-	3,086	2,721	2,721	42.4	4	680.3	121.7	906	100.919	8	6.906	-0.457	nucl
Gh_A01G1468	NLP2	A01	89,923,386	89,926,969	+	3,584	2,796	2,796	44.9	4	699	262.7	931	102.683	-8	5.996	-0.422	nucl
Gh_A01G1560	PB1	A01	92,419,665	92,421,097	-	1,433	1,032	1,032	47.9	2	516	401	343	37.921	9	8.087	-0.705	plas
Gh_A02G0102	NLP8	A02	931,389	935,261	-	3,873	2,979	2,979	42.5	5	595.8	223.5	992	110.336	-11.5	5.71	NA	plas
Gh_A02G0239	PB1	A02	2,978,559	2,983,643	-	5,085	3,291	3,291	40.9	9	365.7	224.3	1,096	122.017	10	6.857	-0.615	plas
Gh_A02G0925	NLP8	A02	35,249,348	35,255,298	-	5,951	2,979	2,979	44	5	595.8	743	992	108.971	-14	5.421	-0.421	E.R.
Gh_A02G0949	NLP7	A02	39,455,243	39,459,094	+	3,852	2,961	2,961	43.7	5	592.2	222.8	986	108.813	-4	6.322	-0.408	plas
Gh_A02G1702	RWP-RK	A02	83,207,000	83,214,947	-	7,948	1,026	1,026	42.7	5	205.2	1,730.50	341	39.437	13	9.42	-0.468	nucl
Gh_A03G0443	RWP-RK	A03	9,533,163	9,534,253	+	1,091	720	720	39.7	3	240	185.5	239	27.795	-2	6.107	-0.578	nucl
Gh_A03G0454	PB1	A03	9,917,433	9,922,383	-	4,951	1,656	1,656	43.9	14	118.3	253.5	551	59.923	3	7.053	-0.222	nucl
Gh_A03G0493	PB1	A03	11,495,867	11,499,341	-	3,475	2,397	2,397	44.1	7	342.4	179.7	798	86.572	-22.5	4.67	-0.33	E.R.
Gh_A03G1178	PB1	A03	84,402,981	84,403,686	-	706	561	561	49.9	2	280.5	145	186	20.594	9.5	9.567	-0.397	nucl
Gh_A03G1567	PB1	A03	96,897,807	96,899,246	-	1,440	1,440	1,440	52.4	1	1,440.00	No intron	479	52.125	-2.5	6.195	-0.576	plas
Gh_A03G1857	NLP2	A03	99,615,766	99,619,026	+	3,261	2,352	2,352	44.4	4	588	303	783	87.029	7.5	7.088	-0.365	nucl
Gh_A04G0995	PB1	A04	59,881,093	59,882,583	-	1,491	1,491	1,491	52.1	1	1,491.00	No intron	496	54.052	-2.5	6.145	-0.582	plas
Gh_A05G0047	PB1	A05	699,481	704,953	-	5,473	4,206	4,206	44.1	9	467.3	158.4	1,401	151.42	-25.5	5.163	-0.425	nucl
Gh_A05G0079	PB1	A05	956,169	957,206	+	1,038	762	762	46.5	4	190.5	92	253	28.112	4.5	7.695	-0.581	E.R.
Gh_A05G0460	PB1	A05	5,042,064	5,044,302	-	2,239	2,145	2,145	43	2	1,072.50	94	714	81.039	-16.5	5.031	-0.496	nucl
Gh_A05G1538	NLP4	A05	15,633,286	15,636,755	-	3,470	2,787	2,787	42.5	4	696.8	227.7	928	103.32	6	6.84	-0.515	nucl
Gh_A05G2263	PB1	A05	26,483,791	26,485,167	+	1,377	1,377	1,377	50.5	1	1,377.00	No intron	458	49.379	-10	4.756	-0.399	nucl
Gh_A05G3286	PB1	A05	86,197,819	86,200,325	-	2,507	819	819	45.8	4	204.8	562.7	272	30.036	4.5	7.7	-0.785	E.R.
Gh_A05G3757	PB1	A05	20,025	21,413	-	1,389	1,389	1,389	47.7	1	1,389.00	No intron	462	50.379	-14	4.536	-0.324	E.R.
Gh_A05G3990	PB1	A05	8,700	15,001	-	6,302	3,306	3,306	41.8	8	413.3	428	1,101	122.187	-23.5	5.639	-0.446	E.R.
Gh_A06G0421	PB1	A06	7,213,066	7,218,001	-	4,936	4,086	4,086	44	9	454	106.3	1,361	147.795	-36	5.054	-0.425	E.R.
Gh_A06G1787	PB1	A06	102,823,406	102,824,767	-	1,362	1,197	1,197	49.5	2	598.5	165	398	43.914	-7	5.518	-0.686	E.R.
Gh_A06G2074	PB1	A06	264,938	268,653	+	3,716	1,467	1,467	51.4	3	489	1,124.50	488	53.145	1	6.615	-0.387	nucl
Gh_A07G0445	PB1	A07	5,749,406	5,756,868	+	7,463	1,644	1,644	43.1	14	117.4	447.6	547	59.991	2	6.852	-0.218	E.R.
Gh_A07G0531	PB1	A07	7,066,078	7,068,526	-	2,449	1,980	1,980	44.5	2	990	469	659	72.175	-12.5	5.089	-0.571	plas
Gh_A07G1460	PB1	A07	42,732,155	42,738,346	+	6,192	3,693	3,693	41.5	9	410.3	312.4	1,230	135.77	-10	6.063	-0.51	plas
Gh_A08G0768	PB1	A08	28,507,225	28,514,227	+	7,003	3,414	3,414	42.7	8	426.8	512.7	1,137	126.045	-19	5.835	-0.572	E.R.
Gh_A08G0810	PB1	A08	36,708,955	36,711,334	-	2,380	2,286	2,286	40.7	2	1,143.00	94	761	87.405	-6	6.011	-0.67	nucl
Gh_A08G1723	PB1	A08	97,169,738	97,176,030	+	6,293	3,834	3,834	41.5	8	479.3	351.3	1,277	140.371	-37.5	4.756	-0.459	plas
Gh_A09G0059	PB1	A09	1,305,385	1,310,594	+	5,210	1,500	1,500	43.7	12	125	337.3	500	54.211	6.5	7.422	-0.164	nucl
Gh_A09G1689	NLP7	A09	70,394,150	70,398,090	-	3,941	2,913	2,913	45.2	5	582.6	257	970	106.503	-14	5.279	-0.362	plas
Gh_A09G2142	RWP-RK	A09	74,495,305	74,496,378	+	1,074	846	846	37.1	4	211.5	76	281	32.752	-4	5.203	-0.904	nucl
Gh_A10G1257	NLP8	A10	65,745,295	65,750,705	+	5,411	2,856	2,856	42.7	5	571.2	638.8	951	104.211	-19	5.012	-0.399	E.R.
Gh_A11G0376	PB1	A11	3,452,434	3,455,210	-	2,777	2,343	2,343	44	6	390.5	86.8	780	84.497	-32.5	4.418	-0.232	mito
Gh_A11G0542	PB1	A11	5,111,393	5,117,277	+	5,885	1,467	1,467	44.6	13	112.8	368.2	488	53.209	4.5	7.284	-0.05	nucl
Gh_A11G1536	PB1	A11	22,168,865	22,170,634	-	1,770	1,431	1,431	47.4	2	715.5	339	476	52.1	0.5	6.552	-0.462	plas
Gh_A11G3016	PB1	A11	24,001	28,527	-	4,527	1,614	1,614	43.2	14	115.3	224.1	537	58.507	8.5	8.358	-0.145	nucl
Gh_A12G0297	NLP2	A12	5,073,568	5,077,178	-	3,611	2,850	2,850	44.1	4	712.5	253.7	949	104.234	4	6.806	-0.424	nucl
Gh_A12G0439	PB1	A12	9,234,626	9,240,522	-	5,897	3,936	3,936	43.3	8	492	280.1	1,311	144.62	-23	5.338	-0.549	nucl
Gh_A12G1336	PB1	A12	70,263,427	70,264,159	+	733	648	648	45.8	2	324	85	215	23.861	16.5	9.722	-0.361	extr
Gh_A12G1611	RWP-RK	A12	77,108,077	77,109,325	+	1,249	912	912	42.8	4	228	112.3	303	35.003	-1	6.003	-0.641	E.R.
Gh_A13G1093	PB1	A13	61,245,484	61,247,601	+	2,118	2,118	2,118	45.1	1	2,118.00	No intron	705	77.988	-15.5	5.059	-0.49	plas
Gh_A13G1599	PB1	A13	74,873,340	74,875,635	+	2,296	2,178	2,178	44.1	2	1,089.00	118	725	82.002	-9.5	5.348	-0.51	nucl
Gh_A13G2318	PB1	A13	53,502	55,644	-	2,143	2,052	2,052	41.4	2	1,026.00	91	683	78.805	-6.5	5.84	-0.622	nucl
Gh_D01G0769	PB1	D01	11,024,851	11,026,875	-	2,025	2,025	2,025	45.7	1	2,025.00	No intron	674	74.46	-23	4.749	-0.529	nucl
Gh_D01G0822	PB1	D01	13,055,087	13,062,895	+	7,809	3,648	3,648	41.9	8	456	594.4	1,215	135.149	-26	5.22	-0.587	nucl
Gh_D01G0872	NLP4	D01	14,334,933	14,338,016	-	3,084	2,721	2,721	42.6	4	680.3	121	906	100.83	9	6.963	-0.478	nucl
Gh_D01G1705	NLP2	D01	53,558,837	53,562,425	+	3,589	2,799	2,799	45	4	699.8	263.3	932	103.031	-11.5	5.764	-0.432	nucl
Gh_D01G1812	PB1	D01	55,593,773	55,596,257	+	2,485	1,170	1,170	48.4	3	390	657.5	389	42.705	16.5	9.222	-0.586	plas
Gh_D02G0126	NLP8	D02	1,020,153	1,023,933	-	3,781	2,907	2,907	42.5	5	581.4	218.5	968	107.451	-11	5.716	-0.446	nucl
Gh_D02G0308	PB1	D02	4,200,579	4,205,625	-	5,047	3,291	3,291	41.4	9	365.7	219.5	1,096	122.036	16.5	7.161	-0.625	plas
Gh_D02G1107	NLP8	D02	31,070,010	31,075,995	-	5,986	2,988	2,988	43.9	5	597.6	749.5	995	109.241	-12.5	5.564	-0.416	E.R.
Gh_D02G1209	RWP-RK	D02	37,862,884	37,865,311	+	2,428	420	420	42.4	5	84	502	139	15.305	-2.5	5.087	0.399	nucl
Gh_D02G1615	PB1	D02	55,879,955	55,880,661	-	707	561	561	50.1	2	280.5	146	186	20.46	9	9.276	-0.32	nucl
Gh_D02G2018	PB1	D02	64,467,830	64,469,269	-	1,440	1,440	1,440	52.6	1	1,440.00	No intron	479	52.184	-2.5	6.195	-0.6	plas
Gh_D02G2296	NLP2	D02	66,690,173	66,693,334	+	3,162	2,349	2,349	44.7	4	587.3	271	782	87.41	13	7.741	-0.405	nucl
Gh_D03G0017	RWP-RK	D03	153,427	156,080	+	2,654	1,011	1,011	43.6	5	202.2	410.8	336	38.769	17	10.02	-0.487	nucl
Gh_D03G0813	NLP7	D03	27,828,859	27,832,737	-	3,879	3,021	3,021	43.7	5	604.2	214.5	1,006	111.275	-2	6.42	NA	plas
Gh_D03G1042	PB1	D03	35,274,434	35,277,944	+	3,511	2,397	2,397	43.9	7	342.4	185.7	798	86.478	-18.5	4.822	-0.324	E.R.
Gh_D03G1084	PB1	D03	36,248,755	36,253,730	+	4,976	1,659	1,659	43.9	14	118.5	255.2	552	60.22	4.5	7.328	-0.238	nucl
Gh_D03G1095	RWP-RK	D03	36,520,900	36,521,990	-	1,091	723	723	40.2	3	241	184	240	27.833	-1	6.314	-0.57	nucl
Gh_D04G0318	PB1	D04	4,805,424	4,807,935	+	2,512	819	819	46.3	4	204.8	564.3	272	30.008	3.5	7.287	-0.754	E.R.
Gh_D04G1546	PB1	D04	47,558,208	47,559,692	-	1,485	1,485	1,485	52.1	1	1,485.00	No intron	494	53.846	-3.5	5.979	-0.597	plas
Gh_D05G0049	PB1	D05	616,062	622,094	+	6,033	2,712	2,712	42.6	10	271.2	369	903	99.894	-23	5.446	-0.508	nucl
Gh_D05G0100	PB1	D05	1,142,405	1,147,902	-	5,498	4,212	4,212	44	9	468	160.8	1,403	151.633	-25	5.239	-0.431	nucl
Gh_D05G0138	PB1	D05	1,429,999	1,431,036	+	1,038	768	768	46.6	4	192	90	255	28.349	5.5	8.261	-0.601	E.R.

Gh_D05G1588	PB1	D05	14,366,395	14,367,742	+	1,348	1,272	1,272	50.7	2	636	76	423	46.722	-7	5.664	-0.607	E.R.
Gh_D05G1709	NLP4	D05	15,401,829	15,405,432	-	3,604	2,823	2,823	42.1	5	564.6	195.3	940	104.656	5.5	6.765	-0.481	nucl
Gh_D05G2083	PB1	D05	19,371,700	19,373,088	+	1,389	1,389	1,389	48	1	1,389.00	No intron	462	50.303	-13	4.589	-0.329	E.R.
Gh_D05G2521	PB1	D05	25,449,304	25,454,365	-	5,062	378	378	48.9	7	54	780.7	125	13.946	8.5	10.149	-0.059	nucl
Gh_D05G2522	PB1	D05	25,456,450	25,457,826	+	1,377	1,377	1,377	50.8	1	1,377.00	No intron	458	49.37	-10	4.756	-0.412	nucl
Gh_D05G3139	PB1	D05	46,703,110	46,706,616	-	3,507	2,130	2,130	49.9	2	1,065.00	1,377.00	709	76.642	-5.5	6.089	-0.603	plas
Gh_D05G3923	PB1	D05	39,779	41,995	+	2,217	2,124	2,124	43	2	1,062.00	93	707	80.207	-15.5	5.056	-0.476	nucl
Gh_D06G0459	PB1	D06	6,615,706	6,620,638	-	4,933	4,083	4,083	44.2	9	453.7	106.3	1,360	147.581	-36	5.055	-0.408	E.R.
Gh_D06G1329	PB1	D06	40,480,075	40,484,082	-	4,008	1,428	1,428	50.6	4	357	860	475	51.769	3	6.839	-0.47	nucl
Gh_D06G2192	PB1	D06	63,659,979	63,661,339	+	1,361	1,149	1,149	49.1	2	574.5	212	382	42.042	-7	5.509	-0.688	nucl
Gh_D07G0509	PB1	D07	5,766,090	5,773,486	+	7,397	1,644	1,644	43.4	14	117.4	442.5	547	59.892	3	7.046	-0.22	E.R.
Gh_D07G0600	PB1	D07	6,876,233	6,878,685	-	2,453	1,980	1,980	44.5	2	990	473	659	72.154	-13.5	5.008	-0.571	plas
Gh_D07G1556	PB1	D07	29,200,757	29,207,952	+	7,196	3,858	3,858	41.8	10	385.8	370.9	1,285	141.359	-20	5.436	-0.511	plas
Gh_D08G0914	PB1	D08	18,932,672	18,939,511	+	6,840	3,414	3,414	42.7	8	426.8	489.4	1,137	126.177	-20	5.792	-0.576	E.R.
Gh_D08G0987	PB1	D08	24,127,452	24,129,831	-	2,380	2,286	2,286	40.6	2	1,143.00	94	761	87.456	-7.5	5.903	-0.638	nucl
Gh_D08G1195	NLP7	D08	38,442,816	38,447,910	-	5,095	2,961	2,961	44.4	5	592.2	533.5	986	108.589	-11	5.707	-0.338	plas
Gh_D08G1828	RWP-RK	D08	55,164,242	55,165,790	+	1,549	1,050	1,050	43.1	6	175	99.8	349	39.63	-3	6.006	-0.714	E.R.
Gh_D08G2074	PB1	D08	59,580,227	59,586,431	+	6,205	3,840	3,840	41.5	8	480	337.9	1,279	140.758	-41	4.682	-0.452	plas
Gh_D09G0055	PB1	D09	1,421,667	1,427,838	+	6,172	1,593	1,593	44.3	14	113.8	352.2	530	57.27	8	7.733	-0.144	nucl
Gh_D09G1795	NLP7	D09	45,604,537	45,608,478	-	3,942	2,913	2,913	45.5	5	582.6	257.3	970	106.307	-19.5	4.979	-0.357	plas
Gh_D10G1228	NLP8	D10	21,658,426	21,663,753	-	5,328	2,856	2,856	42.4	5	571.2	618	951	104.239	-21.5	4.939	-0.393	E.R.
Gh_D11G0397	PB1	D11	3,340,844	3,345,359	-	4,516	1,614	1,614	43.3	14	115.3	223.2	537	58.482	8.5	8.358	-0.15	nucl
Gh_D11G0436	PB1	D11	3,669,127	3,672,465	-	3,339	2,352	2,352	44.2	7	336	164.5	783	84.638	-35.5	4.374	-0.23	nucl
Gh_D11G0626	PB1	D11	5,464,000	5,470,690	+	6,691	1,593	1,593	45.4	14	113.8	392.2	530	57.676	4	7.137	-0.107	nucl
Gh_D11G1701	PB1	D11	18,528,997	18,530,772	-	1,776	1,437	1,437	47.6	2	718.5	339	478	52.273	-0.5	6.467	-0.456	plas
Gh_D12G0368	NLP2	D12	5,577,963	5,581,575	+	3,613	2,850	2,850	44.3	4	712.5	254.3	949	104.371	1.5	6.623	-0.433	nucl
Gh_D12G0440	PB1	D12	7,218,930	7,224,968	-	6,039	3,936	3,936	43.2	8	492	300.4	1,311	144.614	-22.5	5.337	-0.547	nucl
Gh_D12G1752	RWP-RK	D12	49,834,793	49,836,064	+	1,272	903	903	43.3	4	225.8	123	300	34.592	0.5	6.755	-0.68	E.R.
Gh_D13G1358	PB1	D13	42,604,587	42,606,704	+	2,118	2,118	2,118	45.3	1	2,118.00	No intron	705	78.358	-12	5.298	-0.541	E.R.
Gh_D13G1958	PB1	D13	54,986,437	54,988,732	+	2,296	2,178	2,178	44.7	2	1,089.00	118	725	81.947	-6.5	5.617	-0.523	nucl
Gh_D13G2470	PB1	D13	37,306	39,440	+	2,135	2,052	2,052	41.7	2	1,026.00	83	683	78.603	-2.5	6.295	-0.585	nucl
Gh_Sca004734G01	PB1	scaffold	7,350	8,697	-	1,348	1,272	1,272	50.9	2	636	76	423	46.38	-6.5	5.677	-0.538	nucl
Gh_Sca101252G01	PB1	scaffold	68	439	-	372	372	372	40.6	1	372	No intron	124	14.316	-7	4.372	-0.429	mito
Gh_Sca135291G01	PB1	scaffold	120	308	-	189	189	189	49.2	1	189	No intron	63	7.169	-7.5	4.006	-0.717	mito

B. Physiochemical properties of the proteins encoded by the *NLP* genes in *G. arboreum*

Gene ID	Gene Name	Chro.	Start	End	Strand	Length (bp)	Transcript Length (bp)	CDS Length (bp)	CDS GC Content (%)	Exon Number	Mean Exon Length (bp)	Mean Intron Length (bp)	Protein Length (aa)	Molecular Weight (kDa)	Charge	pI	GRAVY	Subcellular localization
Ga01G1008	PB1	Chr01	15,473,058	15,475,082	-	2,025	2,025	2,025	45.6	1	2,025.00	No intron	674	74.464	-21.5	4.773	-0.501	nucl
Ga01G1064	PB1	Chr01	19,142,010	19,150,106	+	8,097	3,660	3,660	41.4	8	457.5	633.9	1,219	135.556	-20.5	5.496	-0.627	E.R.
Ga01G1125	NLP4	Chr01	21,025,005	21,028,146	-	3,142	2,778	2,778	42.5	4	694.5	121.3	925	102.859	6	6.798	-0.452	nucl
Ga01G1831	NLP7	Chr01	85,611,469	85,615,321	-	3,853	2,922	2,922	43.9	6	487	186.2	973	107.191	-5	6.274	-0.389	plas
Ga01G2094	PB1	Chr01	99,418,463	99,421,937	+	3,475	2,397	2,397	44.2	7	342.4	179.7	798	86.5	-19	4.795	-0.338	E.R.
Ga01G2121	PB1	Chr01	100,608,969	100,613,915	-	4,947	1,656	1,656	43.8	14	118.3	253.2	551	59.923	3	7.053	-0.222	nucl
Ga01G2168	RWP-RK	Chr01	102,164,669	102,165,757	+	1,089	720	720	39.7	3	240	184.5	239	27.838	-1	6.315	-0.582	nucl
Ga02G0022	RWP-RK	Chr02	295,220	297,878	+	2,659	966	966	43.9	5	193.2	423.3	321	36.948	13	9.571	-0.53	nucl
Ga02G1194	NLP2	Chr02	88,655,571	88,659,153	+	3,583	2,796	2,796	45	4	699	262.3	931	102.492	-8	5.997	-0.439	nucl
Ga02G1330	RWP-RK	Chr02	91,581,780	91,584,298	-	2,519	1,098	1,098	47.6	4	274.5	473.7	365	40.418	12	8.388	-0.606	plas
Ga03G0114	NLP8	Chr03	933,118	936,979	-	3,862	2,970	2,970	42.4	5	594	223	989	109.873	-10.5	5.785	-0.45	plas
Ga03G0310	PB1	Chr03	3,414,164	3,419,248	-	5,085	3,291	3,291	41	9	365.7	224.3	1,096	122.109	10.5	6.885	-0.624	plas
Ga03G0673	PB1	Chr03	10,137,142	10,139,571	-	2,430	2,226	2,226	43.1	2	1,113.00	204	741	83.413	18	8.11	-0.305	E.R.
Ga03G1010	NLP8	Chr03	30,544,953	30,550,898	+	5,946	2,979	2,979	44	5	595.8	741.8	992	108.906	-13	5.495	-0.425	E.R.
Ga03G1811	PB1	Chr03	118,194,702	118,195,407	-	706	561	561	49.7	2	280.5	145	186	20.564	9	9.251	-0.358	nucl
Ga03G2357	RWP-RK	Chr03	132,187,980	132,189,419	-	1,440	1,440	1,440	52.3	1	1,440.00	No intron	479	52.135	-2.5	6.195	-0.578	plas
Ga03G2697	NLP2	Chr03	135,003,180	135,007,468	+	4,289	2,472	2,472	43.8	5	494.4	454.3	823	91.73	8.5	7.177	-0.287	nucl
Ga04G0362	PB1	Chr04	4,287,007	4,288,497	+	1,491	1,491	1,491	52	1	1,491.00	No intron	496	53.937	-3.5	5.979	-0.584	plas
Ga04G1727	NLP5	Chr04	92,357,331	92,359,836	-	2,506	819	819	45.7	4	204.8	562.3	272	30.064	4.5	7.7	-0.793	E.R.
Ga05G0062	PB1	Chr05	648,941	655,189	+	6,249	3,309	3,309	42	8	413.6	420	1,102	122.242	-21.5	5.733	-0.459	E.R.
Ga05G0123	PB1	Chr05	1,221,676	1,227,148	-	5,473	4,206	4,206	44.2	9	467.3	158.4	1,401	151.391	-26	5.162	-0.425	nucl
Ga05G0157	PB1	Chr05	1,470,064	1,471,098	+	1,035	762	762	46.5	4	190.5	91	253	28.165	5.5	8.262	-0.609	E.R.
Ga05G0605	PB1	Chr05	5,422,760	5,424,998	-	2,239	2,145	2,145	42.9	2	1,072.50	94	714	81.138	-16.5	5.035	-0.505	nucl
Ga05G1166	PB1	Chr05	10,236,830	10,239,277	+	2,448	2,238	2,238	40.5	2	1,119.00	210	745	83.99	17.5	7.858	-0.27	nucl
Ga05G1781	PB1	Chr05	16,240,987	16,242,334	+	1,348	1,257	1,257	51.1	2	628.5	91	418	45.807	-7	5.656	-0.525	E.R.
Ga05G1907	NLP4	Chr05	17,145,404	17,149,022	-	3,619	2,829	2,829	42.5	5	565.8	197.5	942	104.829	6.5	6.86	-0.479	nucl
Ga05G2305	PB1	Chr05	21,452,220	21,453,608	+	1,389	1,389	1,389	47.7	1	1,389.00	No intron	462	50.362	-14	4.536	-0.326	E.R.
Ga05G2814	PB1	Chr05	28,929,525	28,930,901	+	1,377	1,377	1,377	50.6	1	1,377.00	No intron	458	49.347	-10	4.756	-0.418	nucl
Ga05G3626	PB1	Chr05	76,830,437	76,832,828	+	2,392	2,136	2,136	49.9	2	1,068.00	256	711	77.069	-5	6.147	-0.634	plas
Ga06G0496	PB1	Chr06	6,832,290	6,837,221	-	4,932	4,053	4,053	44	10	405.3	97.7	1,350	146.715	-40	4.923	-0.433	E.R.
Ga06G1549	PB1	Chr06	95,228,976	95,232,529	+	3,554	1,542	1,542	49.9	3	514	1,006.00	513	56.107	4	6.912	-0.365	nucl
Ga06G2383	PB1	Chr06	129,806,481	129,807,842	-	1,362	1,272	1,272	49.3	2	636	90	423	46.728	-2.5	6.231	-0.642	E.R.
Ga07G0597	PB1	Chr07	6,594,651	6,602,088	+	7,438	1,638	1,638	43.3	14	117	446.2	545	59.718	3	7.046	-0.222	E.R.
Ga07G0705	PB1	Chr07	8,064,696	8,067,148	-	2,453	1,983	1,983	44.6	2	991.5	470	660	72.281	-12.5	5.089	-0.571	plas
Ga07G1754	PB1	Chr07	44,841,923	44,849,097	+	7,175	3,864	3,864	41.6	11	351.3	331.1	1,287	142.029	-15.5	5.832	-0.502	plas
Ga08G1003	RWP-RK	Chr08	36,431,985	36,439,010	+	7,026	3,426	3,426	42.9	8	428.3	514.3	1,141	126.501	-19.5	5.828	-0.575	E.R.
Ga08G1035	RWP-RK	Chr08	42,571,099	42,573,478	+	2,380	2,286	2,286	40.7	2	1,143.00	94	761	87.299	-6.5	5.939	-0.655	nucl
Ga08G1060	PB1	Chr08	46,685,059	46,687,816	+	2,758	2,535	2,535	43.1	2	1,267.50	223	844	94.539	27	8.726	-0.398	cyto
Ga08G1986	RWP-RK	Chr08	115,797,556	115,815,245	+	17,690	981	981	39.6	6	163.5	3,341.80	326	37.378	4	7.383	-0.597	nucl
Ga08G2277	PB1	Chr08	121,903,582	121,910,792	+	7,211	3,768	3,768	41.8	9	418.7	430.4	1,255	137.582	-39.5	4.689	-0.439	plas

Ga08G2905	PB1	Chr08	128,836,714	128,838,577	+	1,864	1,215	1,215	39.6	7	173.6	108.2	404	45.8	-19	4.542	-0.539	nucl
Ga09G0065	PB1	Chr09	1,329,521	1,335,722	+	6,202	1,593	1,593	44.3	14	113.8	354.5	530	57.203	9.5	8.251	-0.137	nucl
Ga09G2119	PB1	Chr09	79,013,481	79,017,423	-	3,943	2,916	2,916	45.3	5	583.2	256.8	971	106.588	-15	5.218	-0.358	plas
Ga09G2756	PB1	Chr09	84,439,491	84,441,835	-	2,345	2,256	2,256	42.7	2	1,128.00	89	751	84.642	5.5	7.003	-0.391	nucl
Ga09G2758	RWP-RK	Chr09	84,450,923	84,451,999	+	1,077	849	849	37.7	4	212.3	76	282	32.665	-4	5.203	-0.885	nucl
Ga10G1690	NLP8	Chr10	92,139,114	92,144,520	+	5,407	2,856	2,856	42.7	5	571.2	637.8	951	104.301	-20	4.973	-0.391	E.R.
Ga11G0754	FRS3	Chr11	12,939,357	12,941,820	+	2,464	2,331	2,331	44	2	1,165.50	133	776	87.706	9.5	7.374	-0.336	plas
Ga11G2252	PB1	Chr11	101,117,146	101,118,915	+	1,770	1,431	1,431	47.1	2	715.5	339	476	52.15	1	6.601	-0.433	plas
Ga11G3460	PB1	Chr11	118,880,180	118,886,601	-	6,422	1,593	1,593	45.6	14	113.8	371.5	530	57.654	9	8.286	-0.116	nucl
Ga11G3656	PB1	Chr11	120,564,424	120,567,735	+	3,312	2,352	2,352	44.2	7	336	160	783	84.76	-32.5	4.429	-0.248	nucl
Ga11G3698	PB1	Chr11	120,909,781	120,914,314	+	4,534	1,614	1,614	43	14	115.3	224.6	537	58.521	8.5	8.358	-0.146	nucl
Ga12G1060	RWP-RK	Chr12	11,596,165	11,597,413	-	1,249	894	894	43.1	4	223.5	118.3	297	34.316	-1	6.001	-0.715	E.R.
Ga12G1409	PB1	Chr12	19,838,829	19,839,564	-	736	648	648	45.4	2	324	88	215	23.822	15.5	9.57	-0.331	plas
Ga12G2628	PB1	Chr12	97,386,890	97,392,787	-	5,898	3,939	3,939	43.1	8	492.4	279.9	1,312	144.784	-21.5	5.426	-0.548	nucl
Ga13G1636	PB1	Chr13	102,514,965	102,517,081	+	2,117	2,085	2,085	45.2	2	1,042.50	32	694	76.782	-13	5.159	-0.501	plas
Ga13G2231	PB1	Chr13	117,360,813	117,363,720	-	2,908	2,286	2,286	41.8	3	762	311	761	86.215	-3	6.246	-0.375	nucl
Ga13G2276	PB1	Chr13	118,092,601	118,094,896	+	2,296	2,178	2,178	44.2	2	1,089.00	118	725	81.973	-12.5	5.169	-0.509	nucl
Ga13G2501	PB1	Chr13	120,304,240	120,306,382	-	2,143	2,052	2,052	41.3	2	1,026.00	91	683	78.793	-4.5	6.059	-0.614	nucl
Ga14G1659	NLP2	tig00015610	837,745	841,356	-	3,612	2,850	2,850	44.2	4	712.5	254	949	104.265	3.5	6.779	-0.415	nucl
Ga14G2164	NLP7	tig00018630	187,263	191,629	+	4,367	2,961	2,961	44	5	592.2	351.5	986	108.719	-14	5.474	-0.341	nucl

C. Physiochemical properties of the proteins encoded by the *NLP* genes in *G. raimondii*

Gene ID	Gene Name	Chro	Start	End	Strand	Length (bp)	Transcript Length (bp)	CDS Length (bp)	CDS GC Content (%)	Exon Number	Mean Exon Length (bp)	Mean Intron Length (bp)	Protein Length (aa)	Molecular Weight (kDa)	Charge	Isoelectric Point	GRAVY	Subcellular localization
Gorai.001G058100	CBSCBSPB5	Chr01	5738607	5746851	+	8245	2378	1644	43.4	15	158.5	417.3	547	59.918	3	7.046	-0.227	nucl
Gorai.001G068000	NA	Chr01	6818609	6821267	-	2659	2181	1986	44.7	2	1090.5	478	661	72.443	-12.5	5.092	-0.592	plas
Gorai.001G185100	EDR1	Chr01	29557800	29565550	+	7751	4257	3963	41.6	11	387	325.3	1320	145.54	-15	5.771	-0.493	plas
Gorai.002G103800	Ttc1	Chr02	13237618	13240656	-	3039	2832	2025	46	2	1416	207	674	74.428	-23	4.75	-0.533	nucl
Gorai.002G110000	EDR1	Chr02	15142629	15153444	+	10816	4510	3753	41.5	8	563.8	900.1	1250	139.608	-19.5	5.553	-0.578	E.R.
Gorai.002G115800	NLP4	Chr02	16450295	16455270	-	4976	3860	2778	42.7	5	772	279	925	102.796	6	6.798	-0.468	nucl
Gorai.002G206700	NLP2	Chr02	55259990	55264633	+	4644	3473	2799	44.9	5	694.6	292.8	932	102.896	-11.5	5.763	-0.433	nucl
Gorai.002G218300	NA	Chr02	56990540	56993556	-	3017	1734	1032	47.8	4	433.5	314	343	37.787	11.5	8.296	-0.695	plas
Gorai.003G002500	RKD5	Chr03	148877	152037	+	3161	1474	966	44.3	5	294.8	421.8	321	36.948	12	9.401	-0.571	nucl
Gorai.003G063900	GIS2	Chr03	12233508	12236431	+	2924	1137	795	48.1	5	227.4	446.8	264	29.205	18	8.225	-0.509	nucl
Gorai.003G074300	NLP7	Chr03	17754935	17759130	+	4196	3218	2961	43.8	5	643.6	220.3	986	108.771	-4.5	6.293	-0.397	E.R.
Gorai.003G115800	NBR1	Chr03	35238461	35242691	+	4231	3113	2397	44	7	444.7	186.3	798	86.386	-20	4.75	-0.317	E.R.
Gorai.003G120400	CBSCBSPB5	Chr03	36148775	36154621	+	5847	2351	1659	43.8	15	156.7	243	552	60.254	4.5	7.328	-0.239	nucl
Gorai.003G121600	RKD4	Chr03	36396958	36398115	-	1158	789	723	40.4	3	263	184.5	240	27.837	-1	6.314	-0.569	nucl
Gorai.004G101000	STY8	Chr04	18347512	18355724	+	8213	4566	3414	42.8	9	507.3	455.9	1137	126.211	-20	5.792	-0.577	E.R.
Gorai.004G108400	NA	Chr04	22283295	22287561	+	4267	3065	2535	43.2	3	1021.7	601	844	94.459	27	8.726	-0.401	cyto
Gorai.004G109400	unc45b	Chr04	22788713	22792660	-	3948	3649	2286	40.6	3	1216.3	149.5	761	87.433	-7.5	5.904	-0.644	nucl
Gorai.004G131700	NLP7	Chr04	35735761	35741248	-	5488	3361	2961	44.2	5	672.2	531.8	986	108.563	-10.5	5.775	-0.338	plas
Gorai.004G198100	RKD3	Chr04	51913763	51915383	+	1621	1144	1071	42.9	6	190.7	95.4	356	40.479	-4	5.82	-0.665	E.R.
Gorai.004G225000	EDR1	Chr04	55961518	55968882	+	7365	4700	3834	41.5	10	470	296.1	1277	140.517	-38	4.732	-0.445	plas
Gorai.005G013500	NLP8	Chr05	982668	987909	-	5242	3916	2082	42.4	8	489.5	189.4	693	76.919	-1	6.4	-0.457	nucl
Gorai.005G035500	EDR1	Chr05	3370861	3375903	-	5043	3291	3291	41.4	9	365.7	219	1096	122.061	16.5	7.161	-0.624	plas
Gorai.005G072800	NA	Chr05	7999164	8002301	-	3138	2772	2226	43.2	3	924	183	741	83.515	15.5	7.874	-0.335	E.R.
Gorai.005G126000	NLP8	Chr05	28134228	28141832	-	7605	4153	2982	44	7	593.3	575.3	993	109.107	-14	5.485	-0.431	E.R.
Gorai.005G133400	PIPC	Chr05	33490552	33493311	-	2760	759	564	42.6	6	126.5	400.2	187	20.937	1	6.93	0.195	nucl
Gorai.005G178300	NA	Chr05	52259034	52262007	-	2974	2129	561	50.1	4	532.3	273.3	186	20.49	8.5	9.228	-0.368	nucl
Gorai.005G227800	NA	Chr05	61083672	61085543	-	1872	1851	1440	52.5	2	925.5	21	479	52.282	-1.5	6.326	-0.619	plas
Gorai.005G259100	NLP2	Chr05	63487945	63491581	+	3637	2824	2349	44.7	4	706	271	782	87.283	11	7.508	-0.381	nucl
Gorai.006G007000	CBSCBSPB3	Chr06	1417393	1424324	+	6932	2308	1593	44.4	14	164.9	354.8	530	57.17	7	7.481	-0.132	nucl
Gorai.006G206500	NLP7	Chr06	46211226	46215591	-	4366	3345	2913	45.2	5	669	255.3	970	106.376	-21	4.908	-0.352	plas
Gorai.006G266700	FRS6	Chr06	50522060	50527039	-	4980	2904	2253	42.7	4	726	692	750	84.511	6.5	7.111	-0.372	nucl
Gorai.006G266900	RKD1	Chr06	50535326	50536377	+	1052	798	798	36.5	5	159.6	63.5	266	31.326	-5	4.968	-0.851	nucl
Gorai.007G043100	CBSCBSPB5	Chr07	2984522	2990000	-	5479	2470	1614	43.2	15	164.7	214.9	537	58.496	8.5	8.358	-0.15	nucl
Gorai.007G046900	NBR1	Chr07	3261901	3265802	-	3902	2923	2352	44.5	7	417.6	163.2	783	84.461	-35	4.385	-0.243	nucl
Gorai.007G067100	CBSCBSPB3	Chr07	4737488	4744662	+	7175	2036	1593	45.5	15	135.7	367.1	530	57.617	7	7.902	-0.102	nucl
Gorai.007G184900	NA	Chr07	17737913	17740296	-	2384	2045	1437	47.9	2	1022.5	339	478	52.193	0	6.507	-0.473	plas
Gorai.007G309200	NA	Chr07	52275678	52279944	-	4267	3094	2310	43.6	5	618.8	293.3	769	86.953	11.5	7.645	-0.332	plas
Gorai.008G041100	NLP2	Chr08	5356850	5361647	+	4798	3753	2850	44.3	5	750.6	261.3	949	104.46	2.5	6.701	-0.441	nucl
Gorai.008G049200	EDR1	Chr08	6849418	6857973	-	8556	4442	3948	43.1	11	403.8	397.7	1315	145.076	-24.5	5.291	-0.547	nucl
Gorai.008G160900	NA	Chr08	42698996	42700408	+	1413	1325	612	45.6	2	662.5	88	203	22.313	13.5	9.575	-0.347	extr
Gorai.008G193400	RKD1	Chr08	47736225	47737496	+	1272	903	903	43.5	4	225.8	123	300	34.56	0	6.54	-0.68	E.R.
Gorai.009G006600	STY8	Chr09	559527	567335	+	7809	4362	3324	42	10	436.2	369.2	1107	122.843	-24	5.636	-0.476	nucl
Gorai.009G012400	CTR1	Chr09	1023422	1029976	-	6555	5275	4212	44.2	9	586.1	160	1403	151.514	-25.5	5.201	-0.417	nucl
Gorai.009G059900	unc45b	Chr09	4290989	4294322	-	3334	2930	2145	43.1	3	976.7	202	714	81.028	-18.5	4.95	-0.486	nucl
Gorai.009G114800	NA	Chr09	8413698	8418522	+	4825	3011	2238	40.3	4	752.8	604.7	745	83.96	14	7.642	-0.276	nucl
Gorai.009G174700	NA	Chr09	13491337	13494600	+	3264	1982	1257	51.2	3	660.7	641	418	45.998	-7	5.658	-0.575	E.R.
Gorai.009G187500	NLP4	Chr09	14396710	14402027	-	5318	3361	2823	42.5	6	560.2	218.2	940	104.659	3	6.641	-0.488	nucl
Gorai.009G226800	NA	Chr09	17768850	17771608	+	2759	1674	1389	47.9	1	1674	No intron	462	50.367	-13	4.589	-0.352	E.R.
Gorai.009G278600	NA	Chr09	23452279	23454562	+	2284	2054	1368	50.9	2	1027	148	455	49.04	-10	4.756	-0.409	nucl
Gorai.009G348900	NA	Chr09	43769676	43773566	-	3891	3031	2124	50	4	757.8	286.7	707	76.379	-5	6.147	-0.602	plas
Gorai.010G056300	CTR1	Chr10	6416782	6422116	-	5335	4495	4083	44.1	9	499.4	105	1360	147.724	-35.5	5.05	-0.419	E.R.
Gorai.010G148800	NA	Chr10	39711200	39716461	-	5262	1794	1428	50.8	4	448.5	1156	475	51.742	4	6.967	-0.508	nucl
Gorai.010G253900	NA	Chr10	61947447	61948997	-	1551	1462	1272	49.1	2	731	89	423	46.638	-2	6.301	-0.646	nucl
Gorai.011G138700	NLP8	Chr11	21422864	21429703	-	6840	3733	2856	42.6	6	622.2	621.4	951	104.149	-21	4.943	-0.396	E.R.

Gorai.012G016100	NA	Chr12	1837773	1840576	-	2804	994	360	44.2	6	165.7	362	119	13.212	-5.5	4.353	0.233	nucl
Gorai.012G040100	NLP5	Chr12	4989645	5000646	+	11002	976	819	45.8	5	195.2	2503.5	272	30.086	3.5	7.305	-0.771	E.R.
Gorai.012G145600	NA	Chr12	31980879	31983070	-	2192	2192	1485	52	1	2192	No intron	494	53.903	-3.5	5.979	-0.613	plas
Gorai.013G149700	Ttc1	Chr13	40965375	40968365	+	2991	2681	2118	45.1	2	1340.5	310	705	78.513	-12	5.296	-0.535	E.R.
Gorai.013G209900	NA	Chr13	52160839	52164967	-	4129	2940	2286	42	4	735	396.3	761	86.151	0.5	6.548	-0.373	nucl
Gorai.013G214100	unc45b	Chr13	53493233	53496357	+	3125	2768	2178	44.6	3	922.7	178.5	725	81.901	-10.5	5.278	-0.519	nucl
Gorai.013G236600	Ttc1	Chr13	55452501	55455873	-	3373	3197	2052	41.7	3	1065.7	88	683	78.677	-3.5	6.201	-0.582	nucl
