# Supplementary material for: Map-Based Functional Analysis of the GhNLP Genes Reveals Their Roles in Enhancing Tolerance to N-Deficiency in Cotton
Source: Int J Mol Sci. 2019 Oct 8;20(19):4953. doi: 10.3390/ijms20194953 (PMC6801916; doi:10.3390/ijms20194953)
Supplement: Supplementary file 1 [file ijms-20-04953-s001.zip › Table S2.pdf]

Table S2: Duplication analysis of the cotton *NIN* genes

Sequence names		Sd	Sn	S	N	ps	pn	ds	dn	ds/dn	ps/pn	Positive/ purifying selection
Gorai.005G035500	Gorai.005G126000	440.6667	1736.333	659.1667	2322.833	0.6685	0.7475	1.6648	4.2798	0.389	0.8943	yes
Gorai.002G218300	Gorai.003G002500	147.8333	564.1667	212.6667	753.3333	0.6951	0.7489	1.9615	4.8894	0.4012	0.9282	yes
Gh_A11G0542	Gh_A11G1536	240.5	820.5	336.3333	1094.667	0.7151	0.7495	2.2999	5.5528	0.4142	0.954	yes
Gorai.008G049200	Gorai.008G193400	133	536	187.6667	715.3333	0.7087	0.7493	2.1745	5.2337	0.4155	0.9458	yes
Gorai.009G174700	Gorai.009G226800	212.1667	721.8333	294	963	0.7217	0.7496	2.4567	5.5934	0.4392	0.9628	yes
Gorai.009G006600	Gorai.009G012400	487.5	1931.5	729.5	2594.5	0.6683	0.7445	1.6625	3.681	0.4516	0.8977	yes
Gorai.009G187500	Gorai.010G056300	426	1642	623.3333	2199.667	0.6834	0.7465	1.8163	4.0205	0.4518	0.9155	yes
Ga13G2501	Gh_A01G1560	150.3333	609.6667	216.1667	815.8333	0.6955	0.7473	1.9657	4.2182	0.466	0.9306	yes
Gh_A11G3016	Gh_A12G0439	249.8333	927.1667	368	1246	0.6789	0.7441	1.7669	3.6357	0.486	0.9124	yes
Gh_D03G1042	Gh_D03G1084	278	949	389.3333	1269.667	0.714	0.7474	2.2783	4.2601	0.5348	0.9553	yes
Gh_A03G1178	Gh_A05G0460	83	321	125.1667	435.8333	0.6631	0.7365	1.6166	3.0142	0.5363	0.9003	yes
Gorai.005G126000	Gorai.005G178300	88.8333	320.1667	129.3333	431.6667	0.6869	0.7417	1.856	3.3778	0.5495	0.9261	yes
Ga03G1811	Gh_A02G0925	89	319	130	431	0.6846	0.7401	1.8298	3.2486	0.5633	0.925	yes
Gorai.004G198100	Gorai.004G225000	156.5	629.5	224.1667	846.8333	0.6981	0.7434	2.0037	3.545	0.5652	0.9392	yes
Gorai.001G058100	Gorai.001G068000	269.5	937.5	384	1260	0.7018	0.744	2.0589	3.6272	0.5676	0.9433	yes
Gh_D05G0049	Gh_D05G0100	398	1545	601	2111	0.6622	0.7319	1.609	2.7923	0.5762	0.9048	yes
Gorai.004G131700	Gorai.005G013500	307.1667	1195.833	455.1667	1626.833	0.6748	0.7351	1.7254	2.9374	0.5874	0.9181	yes
Gh_A07G0445	Gh_A07G0531	279.6667	943.3333	383	1261	0.7302	0.7481	2.7258	4.4772	0.6088	0.9761	yes
Gh_D02G0308	Gh_D03G1042	371.8333	1378.167	534.3333	1862.667	0.6959	0.7399	1.9717	3.2298	0.6105	0.9405	yes
Gh_D07G0509	Gh_D07G0600	274.5	938.5	384.1667	1259.833	0.7145	0.7449	2.2886	3.749	0.6105	0.9592	yes
Gh_D01G0822	Gh_D02G0308	501.3333	1903.667	719	2572	0.6973	0.7402	1.9911	3.2495	0.6127	0.9421	yes
Gh_A11G1536	Gh_A11G3016	242.6667	818.3333	334.6667	1096.333	0.7251	0.7464	2.5539	4.0101	0.6369	0.9714	yes
Gh_A03G0454	Gh_A03G1178	94.8333	314.1667	135.6667	425.3333	0.699	0.7386	2.0164	3.1422	0.6417	0.9464	yes
Gh_D07G0600	Gh_D07G1556	306.6667	1132.333	442.3333	1537.667	0.6933	0.7364	1.9366	3.0073	0.644	0.9415	yes
Gorai.006G206500	Gorai.006G266900	114.3333	472.6667	161	637	0.7101	0.742	2.2011	3.4073	0.646	0.957	yes
Gh_D11G0397	Gh_D11G0436	259.1667	892.8333	376.5	1216.5	0.6884	0.7339	1.874	2.8826	0.6501	0.9379	yes
Gorai.004G101000	Gorai.004G108400	396.1667	1473.833	552	1983	0.7177	0.7432	2.3586	3.5311	0.6679	0.9656	yes
Gh_D07G1556	Gh_D08G0914	507.6667	1956.333	738.8333	2675.167	0.6871	0.7313	1.8591	2.7684	0.6715	0.9396	yes
Gorai.001G068000	Gorai.001G185100	309.5	1133.5	443.5	1542.5	0.6979	0.7348	1.9996	2.9264	0.6833	0.9497	yes
Gh_D05G0138	Gh_D05G2083	115.1667	437.8333	167.8333	600.1667	0.6862	0.7295	1.8482	2.7005	0.6844	0.9406	yes
Gh_D13G2470	Gh_Sca101252G01	53	218	75.3333	296.6667	0.7035	0.7348	2.0861	2.9256	0.713	0.9574	yes
Gh_D03G1084	Gh_D05G0049	270.1667	953.8333	373.6667	1285.333	0.723	0.7421	2.4936	3.414	0.7304	0.9743	yes
Gorai.008G041100	Gorai.008G049200	453.5	1648.5	627.3333	2222.667	0.7229	0.7417	2.4904	3.3758	0.7377	0.9747	yes
Gh_A04G0995	Gh_A05G0047	254.3333	848.6667	349.5	1141.5	0.7277	0.7435	2.6368	3.5573	0.7412	0.9788	yes
Gorai.003G063900	Gorai.003G120400	127.1667	453.8333	178.6667	616.3333	0.7118	0.7363	2.232	3.0044	0.7429	0.9666	yes
Gh_A12G0439	Gh_A12G1336	105.8333	369.1667	147.8333	500.1667	0.7159	0.7381	2.318	3.1069	0.7461	0.9699	yes
Gorai.007G043100	Gorai.007G046900	263.6667	898.3333	379.1667	1234.833	0.6954	0.7275	1.9648	2.6297	0.7472	0.9559	yes
Ga01G2094	Gh_A01G0794	373.3333	1352.667	536.8333	1860.167	0.6954	0.7272	1.9655	2.6192	0.7504	0.9564	yes
Gh_D09G0055	Gh_D11G0397	268	899	374.3333	1218.667	0.7159	0.7377	2.3189	3.0823	0.7523	0.9705	yes
Gorai.007G309200	Gorai.008G041100	372	1342	508	1802	0.7323	0.7447	2.8092	3.7183	0.7555	0.9833	yes
Gh_Sca101252G01	Gorai.001G058100	57.6667	207.3333	84.1667	287.8333	0.6851	0.7203	1.836	2.4223	0.7579	0.9512	yes
Gh_D01G1812	Gh_D02G1615	94.5	316.5	131.8333	429.1667	0.7168	0.7375	2.3385	3.0693	0.7619	0.972	yes
Gorai.002G110000	Gorai.002G115800	417.8333	1587.167	597.5	2180.5	0.6993	0.7279	2.0206	2.6431	0.7645	0.9607	yes
Gh_A05G3757	Gh_A06G0421	225.5	778.5	321.1667	1067.833	0.7021	0.729	2.0637	2.6833	0.7691	0.9631	yes
Gorai.009G348900	Gorai.010G253900	214	698	308.3333	963.6667	0.6941	0.7243	1.9468	2.5307	0.7693	0.9582	yes
Gorai.010G148800	Gorai.011G138700	243.6667	809.3333	335.8333	1092.167	0.7256	0.741	2.5678	3.32	0.7734	0.9791	yes
Gorai.005G227800	Gorai.006G266700	237.1667	833.8333	322	1118	0.7365	0.7458	3.0154	3.8934	0.7745	0.9876	yes
Ga01G2121	Ga03G0310	260	940	368.1667	1287.833	0.7062	0.7299	2.1304	2.7148	0.7847	0.9675	yes
Gh_A02G0239	Gh_A03G0454	260.8333	939.1667	369	1287	0.7069	0.7297	2.1418	2.7083	0.7908	0.9687	yes
Gh_A07G0531	Gh_A07G1460	307.3333	1122.667	436.6667	1543.333	0.7038	0.7274	2.0906	2.6276	0.7956	0.9675	yes
Gorai.001G185100	Gorai.002G103800	309.6667	1156.333	438.5	1586.5	0.7062	0.7289	2.1303	2.6766	0.7959	0.9689	yes
Gorai.005G259100	Gorai.006G206500	383	1364	519.3333	1829.667	0.7375	0.7455	3.0698	3.8355	0.8004	0.9893	yes
Gorai.006G266700	Gorai.007G043100	252.5	906.5	361	1253	0.6994	0.7235	2.0228	2.5062	0.8071	0.9668	yes
Gh_D05G1709	Gh_A05G3990	437.3333	1639.667	603.3333	2219.667	0.7249	0.7387	2.5468	3.1464	0.8094	0.9813	yes
Gorai.009G012400	Gorai.009G059900	336.1667	1230.833	468.3333	1676.667	0.7178	0.7341	2.3609	2.8901	0.8169	0.9778	yes
Gh_A06G0421	Gh_A07G0445	267.8333	916.1667	380.3333	1263.667	0.7042	0.725	2.097	2.5511	0.822	0.9713	yes
Gh_D08G0987	Gh_D09G0055	245.5	904.5	347	1246	0.7075	0.7259	2.1528	2.5791	0.8347	0.9746	yes
Gorai.003G120400	Gorai.004G101000	269	943	372.8333	1286.167	0.7215	0.7332	2.4527	2.8484	0.8611	0.9841	yes
Gorai.002G103800	Gorai.002G110000	310.8333	1144.167	440.3333	1584.667	0.7059	0.722	2.1253	2.4665	0.8617	0.9777	yes
Gh_D01G0769	Gh_D01G0822	310.8333	1142.167	440.1667	1584.833	0.7062	0.7207	2.1298	2.4315	0.8759	0.9799	yes
Gh_D12G0440	Gh_D13G1358	337.6667	1208.333	467.5	1650.5	0.7223	0.7321	2.4735	2.8015	0.8829	0.9866	yes
Gorai.003G074300	Gorai.003G115800	385.6667	1352.333	538	1859	0.7169	0.7275	2.3393	2.6283	0.89	0.9854	yes
Gh_A01G1560	Gh_A03G0493	168.8333	577.1667	235.8333	796.1667	0.7159	0.7249	2.3181	2.5489	0.9095	0.9875	yes
Gh_A13G2318	Gh_D01G1812	178.6667	672.3333	247.6667	922.3333	0.7214	0.7289	2.45	2.6798	0.9142	0.9896	yes
Gh_D05G2522	Gh_D05G3923	212.5	758.5	305.1667	1071.833	0.6963	0.7077	1.9781	2.1559	0.9175	0.984	yes
Gh_A05G0460	Gh_A05G2263	218.3333	773.6667	305.6667	1071.333	0.7143	0.7222	2.2834	2.47	0.9244	0.9891	yes
Gh_A07G1460	Gh_A08G0768	518.1667	1903.833	737.5	2676.5	0.7026	0.7113	2.0711	2.2235	0.9315	0.9877	yes
Gorai.012G040100	Gorai.013G236600	119.5	460.5	170.3333	648.6667	0.7016	0.7099	2.0549	2.1969	0.9354	0.9882	yes

Gorai.008G193400	Gorai.009G006600	138.5	532.5	186.6667	716.3333	0.742	0.7434	3.4021	3.5462	0.9594	0.9981	yes
Gh_D11G0436	Gh_D11G1701	243.3333	798.6667	336.6667	1100.333	0.7228	0.7258	2.4869	2.5766	0.9652	0.9958	yes
Gh_A06G1787	Gh_A06G2074	209	666	286.5	910.5	0.7295	0.7315	2.6995	2.7754	0.9727	0.9973	yes
Gh_D06G0459	Gh_D06G2192	192.5	637.5	267	882	0.721	0.7228	2.4389	2.4873	0.9805	0.9975	yes
Gh_D11G1701	Gh_D12G0440	235	800	326.6667	1110.333	0.7194	0.7205	2.399	2.4269	0.9885	0.9985	yes
Gorai.005G013500	Gorai.005G035500	320.1667	1171.833	446.8333	1635.167	0.7165	0.7166	2.3319	2.3346	0.9988	0.9998	yes
Gh_D05G3923	Gh_D06G0459	334.6667	1203.333	462	1662	0.7244	0.724	2.5327	2.5223	1.0041	1.0005	no
Gorai.004G108400	Gorai.004G109400	353.3333	1321.667	481.6667	1804.333	0.7336	0.7325	2.8654	2.8182	1.0168	1.0015	no
Gorai.009G226800	Gorai.009G278600	216.8333	708.1667	319.3333	1048.667	0.679	0.6753	1.7682	1.73	1.0221	1.0055	no
Gorai.007G067100	Gorai.007G184900	246.5	802.5	337	1100	0.7315	0.7295	2.7749	2.7014	1.0272	1.0026	no
Gh_D05G2083	Gh_D05G2522	219	714	321.3333	1055.667	0.6815	0.6763	1.7953	1.7406	1.0315	1.0077	no
Gh_D06G2192	Gh_D07G0509	200.8333	639.1667	273.6667	875.3333	0.7339	0.7302	2.8791	2.7257	1.0563	1.005	no
Gh_A09G0059	Gh_A11G0542	204.3333	646.6667	344	1123	0.594	0.5758	1.1776	1.0951	1.0754	1.0315	no
Gh_A08G0768	Gh_A08G0810	344.1667	1295.833	475.8333	1810.167	0.7233	0.7159	2.5013	2.3173	1.0794	1.0104	no
Gh_A05G2263	Gh_A05G3757	220.3333	708.6667	321.6667	1055.333	0.685	0.6715	1.834	1.6928	1.0834	1.0201	no
Gorai.005G178300	Gorai.006G007000	99.5	315.5	134	427	0.7425	0.7389	3.4576	3.1582	1.0948	1.005	no
Gh_Sca135291G01	Gorai.003G074300	27.8333	102.1667	39.8333	149.1667	0.6987	0.6849	2.0124	1.8333	1.0977	1.0202	no
Gorai.002G115800	Gorai.002G206700	442.5	1588.5	600.6667	2177.333	0.7367	0.7296	3.0232	2.702	1.1189	1.0098	no
Gh_A08G0810	Gh_A09G0059	233	825	324.8333	1175.167	0.7173	0.702	2.3493	2.0621	1.1393	1.0217	no
Gh_D08G0914	Gh_D08G0987	347	1297	475.5	1810.5	0.7298	0.7164	2.7092	2.3286	1.1634	1.0187	no
Ga03G2697	Gh_A01G0844	394.5	1395.5	536.1667	1935.833	0.7358	0.7209	2.974	2.4364	1.2206	1.0207	no
Gh_A13G1599	Gh_A13G2318	304.1667	1149.833	418.1667	1633.833	0.7274	0.7038	2.626	2.0897	1.2566	1.0336	no
Gh_A12G1336	Gh_A13G1093	115.6667	356.3333	156.1667	491.8333	0.7407	0.7245	3.2895	2.5361	1.2971	1.0223	no
Gh_D04G1546	Gh_D05G1588	221.6667	711.3333	298.1667	973.8333	0.7434	0.7304	3.5534	2.7352	1.2991	1.0178	no
Gh_D13G1958	Gh_D13G2470	308.3333	1161.667	419.8333	1632.167	0.7344	0.7117	2.9055	2.2316	1.302	1.0319	no
Gorai.007G046900	Gorai.007G309200	385.1667	1300.833	519	1791	0.7421	0.7263	3.418	2.5915	1.3189	1.0218	no
Gh_D13G1358	Gh_D13G1958	325.5	1143.5	451	1667	0.7217	0.686	2.4587	1.8455	1.3323	1.0521	no
Gorai.008G160900	Gorai.009G114800	104.8333	336.1667	141.6667	470.3333	0.74	0.7147	3.2381	2.293	1.4122	1.0353	no
Gorai.011G138700	Gorai.012G016100	57.8333	208.1667	77.3333	282.6667	0.7478	0.7364	4.3892	3.0096	1.4584	1.0155	no
Gorai.013G149700	Gorai.013G214100	328.6667	1144.333	450.1667	1667.833	0.7301	0.6861	2.722	1.8473	1.4735	1.0641	no
Gh_A13G1093	Gh_A13G1599	329	1144	450.1667	1667.833	0.7308	0.6859	2.7505	1.845	1.4908	1.0655	no
Gorai.009G114800	Gorai.009G174700	214.6667	720.3333	286.5	970.5	0.7493	0.7422	5.204	3.4273	1.5184	1.0095	no
Gorai.012G145600	Gorai.013G149700	255.3333	833.6667	341.5	1143.5	0.7477	0.729	4.3345	2.6834	1.6153	1.0256	no
Gh_Sca004734G01	Gh_Sca135291G01	32.1667	101.8333	43.3333	145.6667	0.7423	0.6991	3.4349	2.0174	1.7026	1.0618	no
Gorai.004G109400	Gorai.004G131700	362.8333	1288.167	485.6667	1800.333	0.7471	0.7155	4.1622	2.3097	1.802	1.0441	no
Gorai.010G253900	Gorai.012G145600	223.6667	696.3333	299.1667	972.8333	0.7476	0.7158	4.3186	2.3154	1.8652	1.0445	no
Gorai.003G002500	Gorai.003G063900	129.1667	444.8333	172.6667	622.3333	0.7481	0.7148	4.4717	2.2939	1.9494	1.0466	no
Gh_A03G1567	Gh_A04G0995	220.8333	519.1667	338.8333	1101.167	0.6517	0.4715	1.5244	0.7429	2.0519	1.3824	no
Gh_D02G2018	Gh_D04G1546	219	514	337.3333	1102.667	0.6492	0.4661	1.5053	0.7287	2.0657	1.3927	no
Ga01G1008	Gh_A01G0750	8	6	443	1582	0.0181	0.0038	0.0183	0.0038	4.8075	4.7615	no
