# Supplementary material for: Map-Based Functional Analysis of the GhNLP Genes Reveals Their Roles in Enhancing Tolerance to N-Deficiency in Cotton
Source: Int J Mol Sci. 2019 Oct 8;20(19):4953. doi: 10.3390/ijms20194953 (PMC6801916; doi:10.3390/ijms20194953)
Supplement: Supplementary file 1 [file ijms-20-04953-s001.zip › Table S3.pdf]

Supplementary Table 3: Cis promoter analysis of the *G. hirsutum NLP* genes

Gene ID	Factor or Site Name	Loc.	(Str.)	Signal Sequence	Function
Gh_A09G0059	LTRE1HVBLT49	377	(-)	CCGAAA	"LTRE-1" (low-temperature-responsive element)
Gh_A11G0542	CBFHV	103	(+)	RYCGAC	dehydration-responsive element (DRE) binding proteins
Gh_D09G0055	LTRE1HVBLT49	377	(-)	CCGAAA	"LTRE-1" (low-temperature-responsive element)
Gh_D11G0626	CBFHV	103	(+)	RYCGAC	dehydration-responsive element (DRE) binding proteins
Gh_A03G0454	LTRE1HVBLT49	100	(+)	CCGAAA	"LTRE-1" (low-temperature-responsive element)
Gh_A07G0445	ASF1MOTIFCAMV	1319	(+)	TGACG	A xenobiotic-stress-activated transcription factor/ Abiotic and biotic stress differentially stimulate as-1 element
Gh_A11G3016	CBFHV	1496	(+)	RYCGAC	dehydration-responsive element (DRE) binding proteins
Gh_D03G1084	LTRE1HVBLT49	100	(+)	CCGAAA	"LTRE-1" (low-temperature-responsive element)
Gh_D07G0509	ASF1MOTIFCAMV	1319	(+)	TGACG	A xenobiotic-stress-activated transcription factor/ Abiotic and biotic stress differentially stimulate as-1 element
Gh_D11G0397	CBFHV	1496	(+)	RYCGAC	dehydration-responsive element (DRE) binding proteins
Gh_A01G0794	CBFHV	3726	(-)	RYCGAC	dehydration-responsive element (DRE) binding proteins
Gh_A05G0047	CBFHV	1406	(-)	RYCGAC	dehydration-responsive element (DRE) binding proteins
Gh_A06G0421	LTRE1HVBLT49	3256	(-)	CCGAAA	"LTRE-1" (low-temperature-responsive element)
Gh_D05G0100	CBFHV	1406	(-)	RYCGAC	dehydration-responsive element (DRE) binding proteins
Gh_D06G0459	LTRE1HVBLT49	3253	(-)	CCGAAA	"LTRE-1" (low-temperature-responsive element)
Gh_A02G0239	LTRE1HVBLT49	3058	(-)	CCGAAA	"LTRE-1" (low-temperature-responsive element)
Gh_A07G1460	MYCCONSENSUSAT	47	(-)	CANNTG	abiotic stress signalling in plants
Gh_A08G1723	ACGTABREMOTIFA2OSEM	643	(-)	ACGTGKC	ABA-responsive expression
Gh_A12G0439	CBFHV	3706	(-)	RYCGAC	dehydration-responsive element (DRE) binding proteins
Gh_D02G0308	LTRE1HVBLT49	3058	(-)	CCGAAA	"LTRE-1" (low-temperature-responsive element)
Gh_D05G0049	ASF1MOTIFCAMV	330	(-)	TGACG	A xenobiotic-stress-activated transcription factor/ Abiotic and biotic stress differentially stimulate as-1 element
Gh_D07G1556	ASF1MOTIFCAMV	22	(-)	TGACG	A xenobiotic-stress-activated transcription factor/ Abiotic and biotic stress differentially stimulate as-1 element
Gh_D08G2074	ACGTABREMOTIFA2OSEM	649	(-)	ACGTGKC	ABA-responsive expression
Gh_D12G0440	CBFHV	3706	(-)	RYCGAC	dehydration-responsive element (DRE) binding proteins
Gh_A05G0079	MYCCONSENSUSAT	10	(-)	CANNTG	abiotic stress signalling in plants
Gh_D05G0138	MYCCONSENSUSAT	10	(-)	CANNTG	abiotic stress signalling in plants
Gh_A01G1560	ASF1MOTIFCAMV	2	(+)	TGACG	A xenobiotic-stress-activated transcription factor/ Abiotic and biotic stress differentially stimulate as-1 element
Gh_A03G1178	CBFHV	116	(-)	RYCGAC	dehydration-responsive element (DRE) binding proteins
Gh_A03G1567	CBFHV	65	(+)	RYCGAC	dehydration-responsive element (DRE) binding proteins
Gh_A04G0995	ACGTABREMOTIFA2OSEM	1359	(-)	ACGTGKC	ABA-responsive expression
Gh_A05G2263	CBFHV	65	(+)	RYCGAC	dehydration-responsive element (DRE) binding proteins
Gh_A05G3757	CBFHV	1149	(-)	RYCGAC	dehydration-responsive element (DRE) binding proteins
Gh_A06G1787	ACGTABREMOTIFA2OSEM	576	(-)	ACGTGKC	ABA-responsive expression
Gh_A06G2074	ACGTABREMOTIFA2OSEM	4	(-)	ACGTGKC	ABA-responsive expression
Gh_A07G0531	CBFHV	1028	(-)	RYCGAC	dehydration-responsive element (DRE) binding proteins
Gh_A11G1536	CBFHV	135	(+)	RYCGAC	dehydration-responsive element (DRE) binding proteins
Gh_A12G1336	ASF1MOTIFCAMV	419	(-)	TGACG	A xenobiotic-stress-activated transcription factor/ Abiotic and biotic stress differentially stimulate as-1 element
Gh_D01G1812	LTRE1HVBLT49	941	(+)	CCGAAA	"LTRE-1" (low-temperature-responsive element)
Gh_D02G1209	MYCCONSENSUSAT	161	(-)	CANNTG	abiotic stress signalling in plants
Gh_D02G1615	CBFHV	116	(-)	RYCGAC	dehydration-responsive element (DRE) binding proteins
Gh_D02G2018	CBFHV	65	(+)	RYCGAC	dehydration-responsive element (DRE) binding proteins
Gh_D04G1546	ACGTABREMOTIFA2OSEM	1353	(-)	ACGTGKC	ABA-responsive expression
Gh_D05G1588	ACGTABREMOTIFA2OSEM	536	(+)	ACGTGKC	ABA-responsive expression
Gh_D05G2083	CBFHV	1149	(-)	RYCGAC	dehydration-responsive element (DRE) binding proteins
Gh_D05G2522	CBFHV	65	(+)	RYCGAC	dehydration-responsive element (DRE) binding proteins
Gh_D05G3139	CBFHV	248	(-)	RYCGAC	dehydration-responsive element (DRE) binding proteins
Gh_D06G1329	ABRECE1HVA22	920	(+)	TGCCACCGG	"ABRE (ABA responsive element)/dehydration-responsive
Gh_D06G2192	CBFHV	725	(+)	RYCGAC	dehydration-responsive element (DRE) binding proteins
Gh_D07G0600	CBFHV	1031	(-)	RYCGAC	dehydration-responsive element (DRE) binding proteins
Gh_D11G1701	CBFHV	135	(+)	RYCGAC	dehydration-responsive element (DRE) binding proteins
Gh_Sca004734G01	ACGTABREMOTIFA2OSEM	536	(+)	ACGTGKC	ABA-responsive expression
Gh_Sca101252G01	MYCCONSENSUSAT	44	(-)	CANNTG	abiotic stress signalling in plants

Gh_Sca135291G01	CBFHV	40	(+)	RYCGAC	dehydration-responsive element (DRE) binding proteins
Gh_A03G0493	CBFHV	1040	(+)	RYCGAC	dehydration-responsive element (DRE) binding proteins
Gh_A11G0376	ACGTABREMOTIFA2OSEM	399	(+)	ACGTGKC	ABA-responsive expression
Gh_D03G1042	CBFHV	1040	(+)	RYCGAC	dehydration-responsive element (DRE) binding proteins
Gh_D11G0436	ACGTABREMOTIFA2OSEM	408	(+)	ACGTGKC	ABA-responsive expression
Gh_A01G1468	LTRE1HVBLT49	230	(+)	CCGAAA	"LTRE-1" (low-temperature-responsive element)
Gh_A03G1857	CBFHV	2203	(-)	RYCGAC	dehydration-responsive element (DRE) binding proteins
Gh_A12G0297	CBFHV	278	(+)	RYCGAC	dehydration-responsive element (DRE) binding proteins
Gh_D01G1705	ACGTABREMOTIFA2OSEM	1271	(+)	ACGTGKC	ABA-responsive expression
Gh_D02G2296	CBFHV	136	(-)	RYCGAC	dehydration-responsive element (DRE) binding proteins
Gh_D05G2521	CBFHV	147	(+)	RYCGAC	dehydration-responsive element (DRE) binding proteins
Gh_D12G0368	CBFHV	1084	(+)	RYCGAC	dehydration-responsive element (DRE) binding proteins
Gh_A01G0844	CBFHV	1977	(+)	RYCGAC	dehydration-responsive element (DRE) binding proteins
Gh_A05G1538	CBFHV	287	(-)	RYCGAC	dehydration-responsive element (DRE) binding proteins
Gh_D01G0872	CBFHV	1977	(+)	RYCGAC	dehydration-responsive element (DRE) binding proteins
Gh_D05G1709	ASF1MOTIFCAMV	2016	(-)	TGACG	A xenobiotic-stress-activated transcription factor/ Abiotic and biotic stress differentially stimulate as-1 element
Gh_A05G3286	CBFHV	71	(+)	RYCGAC	dehydration-responsive element (DRE) binding proteins
Gh_D04G0318	CBFHV	71	(+)	RYCGAC	dehydration-responsive element (DRE) binding proteins
Gh_A02G0949	LTRE1HVBLT49	2755	(-)	CCGAAA	"LTRE-1" (low-temperature-responsive element)
Gh_A09G1689	CBFHV	417	(+)	RYCGAC	dehydration-responsive element (DRE) binding proteins
Gh_D03G0813	CBFHV	254	(-)	RYCGAC	dehydration-responsive element (DRE) binding proteins
Gh_D08G1195	CBFHV	471	(-)	RYCGAC	dehydration-responsive element (DRE) binding proteins
Gh_D09G1795	CBFHV	417	(+)	RYCGAC	dehydration-responsive element (DRE) binding proteins
Gh_A02G0102	CBFHV	80	(-)	RYCGAC	dehydration-responsive element (DRE) binding proteins
Gh_A02G0925	ACGTABREMOTIFA2OSEM	260	(-)	ACGTGKC	ABA-responsive expression
Gh_A10G1257	LTRE1HVBLT49	1539	(-)	CCGAAA	"LTRE-1" (low-temperature-responsive element)
Gh_D02G0126	CBFHV	7	(-)	RYCGAC	dehydration-responsive element (DRE) binding proteins
Gh_D02G1107	ACGTABREMOTIFA2OSEM	260	(-)	ACGTGKC	ABA-responsive expression
Gh_D10G1228	ACGTABREMOTIFA2OSEM	1158	(-)	ACGTGKC	ABA-responsive expression
Gh_A09G2142	ASF1MOTIFCAMV	718	(-)	TGACG	A xenobiotic-stress-activated transcription factor/ Abiotic and biotic stress differentially stimulate as-1 element
Gh_D12G1752	CBFHV	783	(-)	RYCGAC	dehydration-responsive element (DRE) binding proteins
Gh_A12G1611	MYCCONSENSUSAT	270	(-)	CANNTG	abiotic stress signalling in plants
Gh_D08G1828	CBFHV	664	(-)	RYCGAC	dehydration-responsive element (DRE) binding proteins
Gh_A03G0443	CBFHV	147	(+)	RYCGAC	dehydration-responsive element (DRE) binding proteins
Gh_D03G1095	CBFHV	147	(+)	RYCGAC	dehydration-responsive element (DRE) binding proteins
Gh_A02G1702	MYCCONSENSUSAT	352	(-)	CANNTG	abiotic stress signalling in plants
Gh_D03G0017	MYCCONSENSUSAT	346	(-)	CANNTG	abiotic stress signalling in plants
Gh_D01G0822	CBFHV	2969	(-)	RYCGAC	dehydration-responsive element (DRE) binding proteins
Gh_A05G3990	ASF1MOTIFCAMV	408	(+)	TGACG	A xenobiotic-stress-activated transcription factor/ Abiotic and biotic stress differentially stimulate as-1 element
Gh_A08G0768	LTRE1HVBLT49	3131	(-)	CCGAAA	"LTRE-1" (low-temperature-responsive element)
Gh_D08G0914	CBFHV	2571	(+)	RYCGAC	dehydration-responsive element (DRE) binding proteins
Gh_A01G0750	CBFHV	1357	(-)	RYCGAC	dehydration-responsive element (DRE) binding proteins
Gh_A13G1093	MYCCONSENSUSAT	101	(-)	CANNTG	abiotic stress signalling in plants
Gh_A13G2318	LTRE1HVBLT49	1229	(-)	CCGAAA	"LTRE-1" (low-temperature-responsive element)
Gh_D01G0769	ASF1MOTIFCAMV	483	(-)	TGACG	A xenobiotic-stress-activated transcription factor/ Abiotic and biotic stress differentially stimulate as-1 element
Gh_D13G1358	CBFHV	457	(+)	RYCGAC	dehydration-responsive element (DRE) binding proteins
Gh_D13G2470	LTRE1HVBLT49	744	(-)	CCGAAA	"LTRE-1" (low-temperature-responsive element)
Gh_A05G0460	CBFHV	166	(+)	RYCGAC	dehydration-responsive element (DRE) binding proteins
Gh_A08G0810	LTRE1HVBLT49	642	(-)	CCGAAA	"LTRE-1" (low-temperature-responsive element)
Gh_A13G1599	CBFHV	9	(+)	RYCGAC	dehydration-responsive element (DRE) binding proteins
Gh_D05G3923	CBFHV	166	(+)	RYCGAC	dehydration-responsive element (DRE) binding proteins
Gh_D08G0987	LTRE1HVBLT49	562	(+)	CCGAAA	"LTRE-1" (low-temperature-responsive element)
Gh_D13G1958	CBFHV	9	(+)	RYCGAC	dehydration-responsive element (DRE) binding proteins
