# Supplementary material for: Map-Based Functional Analysis of the GhNLP Genes Reveals Their Roles in Enhancing Tolerance to N-Deficiency in Cotton
Source: Int J Mol Sci. 2019 Oct 8;20(19):4953. doi: 10.3390/ijms20194953 (PMC6801916; doi:10.3390/ijms20194953)
Supplement: Supplementary file 1 [file ijms-20-04953-s001.zip › Table S4.pdf]

Supplementary Table 4: miRNAs predicted to be targeting various upland cotton, *G. hirsutum NLP* genes

miRNA_Acc.	**Target_Acc.**	**miRNA_start**	**miRNA_end**	**Target_start**	**Target_end**	**miRNA_aligned_fragment**	**Target_aligned_fragment**	**Inhibition**
ghr-miR160	Gh_D11G0436	1	21	1663	1683	UAUGAGGAGCCAUGCAUGUAU	CAACAUGUCUGGGUUCUCAUC	Cleavage
ghr-miR162a	Gh_D05G1709	1	21	390	410	UCGAUAAACCUCUGCAUCCAG	UGUAAUGCAGAGGUUAAUUGG	Cleavage
ghr-miR164	Gh_D03G1095	1	21	117	137	UGGAGAAGCAGGGCACGUGCA	UUCACCUGCAUUGCUUCCCCA	Cleavage
ghr-miR164	Gh_A03G0443	1	21	117	137	UGGAGAAGCAGGGCACGUGCA	UUCACCUGCAUUGCUUCCCCA	Cleavage
ghr-miR164	Gh_A12G0439	1	21	1060	1080	UGGAGAAGCAGGGCACGUGCA	GCUACUGGUUCUGCUUCUUUG	Cleavage
ghr-miR164	Gh_D12G0440	1	21	1060	1080	UGGAGAAGCAGGGCACGUGCA	GCUACUGGUUCUGCUUCUUUG	Cleavage
ghr-miR166b	Gh_A07G1460	1	21	66	86	UCGGACCAGGCUUCAUUCCCC	UGAGAAGAGAGUUUGGUUCGA	Cleavage
ghr-miR167a	Gh_A13G2318	1	21	1838	1857	UGAAGCUGCCAGCAUGAUCUA	UUGAACAUGCUGG-AGCUUCA	Cleavage
ghr-miR167a	Gh_D08G0987	1	21	2060	2079	UGAAGCUGCCAGCAUGAUCUA	UUGAGCAUGCUGG-AGCUUCU	Cleavage
ghr-miR167a	Gh_A08G0810	1	21	2060	2079	UGAAGCUGCCAGCAUGAUCUA	UUGAGCAUGCUGG-AGCUUCU	Cleavage
ghr-miR167a	Gh_D13G2470	1	21	1838	1857	UGAAGCUGCCAGCAUGAUCUA	UUGAACUUGCUGG-AGCUUCA	Cleavage
ghr-miR167b	Gh_A13G2318	1	21	1838	1857	UGAAGCUGCCAGCAUGAUCUA	UUGAACAUGCUGG-AGCUUCA	Cleavage
ghr-miR167b	Gh_D08G0987	1	21	2060	2079	UGAAGCUGCCAGCAUGAUCUA	UUGAGCAUGCUGG-AGCUUCU	Cleavage
ghr-miR167b	Gh_A08G0810	1	21	2060	2079	UGAAGCUGCCAGCAUGAUCUA	UUGAGCAUGCUGG-AGCUUCU	Cleavage
ghr-miR167b	Gh_D13G2470	1	21	1838	1857	UGAAGCUGCCAGCAUGAUCUA	UUGAACUUGCUGG-AGCUUCA	Cleavage
ghr-miR169a	Gh_D08G2074	1	21	2141	2161	UAGCCAAGGAUGACUUGCCUG	UGGUCACUUCAUCCUUGGAUA	Cleavage
ghr-miR169a	Gh_A08G1723	1	21	2135	2155	UAGCCAAGGAUGACUUGCCUG	UGGUCACUUCAUCCUUGGAUA	Cleavage
ghr-miR169a	Gh_D09G0055	1	21	614	634	UAGCCAAGGAUGACUUGCCUG	UGUUCAAGCCAUCCUUGUCUA	Cleavage
ghr-miR169a	Gh_A09G0059	1	21	617	637	UAGCCAAGGAUGACUUGCCUG	UGUUCAAGCCAUCCUUGUCUA	Cleavage
ghr-miR172	Gh_A12G0439	1	21	1288	1308	AGAAUCCUGAUGAUGCUGCAG	UUGCAGUAUAGUUAUGGUUCU	Cleavage
ghr-miR172	Gh_D12G0440	1	21	1288	1308	AGAAUCCUGAUGAUGCUGCAG	UUGCAGUAUAGUUAUGGUUCU	Cleavage
ghr-miR172	Gh_A13G2318	1	21	908	927	AGAAUCCUGAUGAUGCUGCAG	CAGCAGUAUCA-CAGGGUUCC	Translation
ghr-miR2948-5p	Gh_A01G0750	1	22	1078	1099	UGUGGGAGAGUUGGGCAAGAAU	GAGUUUGCUCAACUUUUCCGCA	Cleavage
ghr-miR2948-5p	Gh_D01G0769	1	22	1078	1099	UGUGGGAGAGUUGGGCAAGAAU	GAGUUUGCUCAACUUUUCCGCA	Cleavage
ghr-miR2948-5p	Gh_D13G1358	1	22	1186	1207	UGUGGGAGAGUUGGGCAAGAAU	GAGUUUGCUCAGCUUUUCCGCA	Cleavage
ghr-miR2948-5p	Gh_A13G1093	1	22	1186	1207	UGUGGGAGAGUUGGGCAAGAAU	GAGUUUGCUCAGCUUUUCCGCA	Cleavage
ghr-miR2948-5p	Gh_D03G1042	1	22	1256	1277	UGUGGGAGAGUUGGGCAAGAAU	GAGUUGGCCCAGCUCUCCCUCA	Cleavage
ghr-miR2948-5p	Gh_D05G3139	1	22	753	774	UGUGGGAGAGUUGGGCAAGAAU	CUCCGUUCCUGAUUCUCCUAUG	Cleavage
ghr-miR2948-5p	Gh_A03G0493	1	22	1256	1277	UGUGGGAGAGUUGGGCAAGAAU	GAGUUGGCCCAGCACUCCCUCA	Cleavage
ghr-miR2949a-5p	Gh_D07G1556	1	22	243	264	ACUUUUGAACUGGAUUUGCCGA	UGCCCGAAUUCAGUUGAAAAGA	Cleavage
ghr-miR2949a-5p	Gh_A07G1460	1	22	117	138	ACUUUUGAACUGGAUUUGCCGA	UGCCCGAAUUCAGUUGAAAAGA	Cleavage
ghr-miR2949a-5p	Gh_D09G1795	1	22	2007	2027	ACUUUUGAACUGGAUUUGCCGA	AUGGCCAA-CCAGUUUGAAAGG	Cleavage
ghr-miR2949b	Gh_D07G1556	1	22	243	264	UCUUUUGAACUGGAUUUGCCGA	UGCCCGAAUUCAGUUGAAAAGA	Cleavage
ghr-miR2949b	Gh_A07G1460	1	22	117	138	UCUUUUGAACUGGAUUUGCCGA	UGCCCGAAUUCAGUUGAAAAGA	Cleavage
ghr-miR2949b	Gh_D09G1795	1	22	2007	2027	UCUUUUGAACUGGAUUUGCCGA	AUGGCCAA-CCAGUUUGAAAGG	Cleavage
ghr-miR2949b	Gh_A05G3990	1	22	2152	2173	UCUUUUGAACUGGAUUUGCCGA	CAGGCAAAACAGGUUGAAAAGG	Cleavage
ghr-miR2949c	Gh_D07G1556	1	22	243	264	UCUUUUGAACUGGAUUUGCCGA	UGCCCGAAUUCAGUUGAAAAGA	Cleavage
ghr-miR2949c	Gh_A07G1460	1	22	117	138	UCUUUUGAACUGGAUUUGCCGA	UGCCCGAAUUCAGUUGAAAAGA	Cleavage
ghr-miR2949c	Gh_D09G1795	1	22	2007	2027	UCUUUUGAACUGGAUUUGCCGA	AUGGCCAA-CCAGUUUGAAAGG	Cleavage
ghr-miR2949c	Gh_A05G3990	1	22	2152	2173	UCUUUUGAACUGGAUUUGCCGA	CAGGCAAAACAGGUUGAAAAGG	Cleavage
ghr-miR2950	Gh_D05G0100	1	21	3169	3189	UGGUGUGCAGGGGGUGGAAUA	CCUUCCACUCCUGACAUACCA	Cleavage
ghr-miR2950	Gh_A05G0047	1	21	3169	3189	UGGUGUGCAGGGGGUGGAAUA	CCUUCCACUCCUGACAUACCA	Cleavage
ghr-miR2950	Gh_A06G2074	1	21	400	420	UGGUGUGCAGGGGGUGGAAUA	CGUUCCACCUCCUUCGCAGCU	Cleavage

ghr-miR2950	Gh_D06G1329	1	21	361	381	UGGUGUGCAGGGGGUGGAAUA	CGUUCCACCUCCUUCGCAGCC	Cleavage
ghr-miR396a	Gh_A05G0047	1	21	3082	3102	UUCCACAGCUUUCUUGAACUG	CAGUCCACAAAAGCUGAGGAA	Cleavage
ghr-miR396a	Gh_D05G0100	1	21	3082	3102	UUCCACAGCUUUCUUGAACUG	CAGUCCACAAAAGCUGAGGAA	Cleavage
ghr-miR396a	Gh_D10G1228	1	21	1069	1089	UUCCACAGCUUUCUUGAACUG	UACACCGAGGAAGCUGUGGAU	Cleavage
ghr-miR396a	Gh_D07G1556	1	21	813	833	UUCCACAGCUUUCUUGAACUG	AGGUUCUAAAAAGCUGAGGAU	Cleavage
ghr-miR396a	Gh_A07G1460	1	21	645	665	UUCCACAGCUUUCUUGAACUG	AGGUUCUAAAAAGCUGAGGAU	Cleavage
ghr-miR396a	Gh_D08G2074	1	21	1281	1300	UUCCACAGCUUUCUUGAACUG	AAGUUCU-GAGAACUGUGGAA	Cleavage
ghr-miR396a	Gh_A08G1723	1	21	1275	1294	UUCCACAGCUUUCUUGAACUG	AAGUUCU-GAGAACUGUGGAA	Cleavage
ghr-miR396b	Gh_A05G0047	1	21	3082	3102	UUCCACAGCUUUCUUGAACUG	CAGUCCACAAAAGCUGAGGAA	Cleavage
ghr-miR396b	Gh_D05G0100	1	21	3082	3102	UUCCACAGCUUUCUUGAACUG	CAGUCCACAAAAGCUGAGGAA	Cleavage
ghr-miR396b	Gh_D10G1228	1	21	1069	1089	UUCCACAGCUUUCUUGAACUG	UACACCGAGGAAGCUGUGGAU	Cleavage
ghr-miR396b	Gh_D07G1556	1	21	813	833	UUCCACAGCUUUCUUGAACUG	AGGUUCUAAAAAGCUGAGGAU	Cleavage
ghr-miR396b	Gh_A07G1460	1	21	645	665	UUCCACAGCUUUCUUGAACUG	AGGUUCUAAAAAGCUGAGGAU	Cleavage
ghr-miR396b	Gh_D08G2074	1	21	1281	1300	UUCCACAGCUUUCUUGAACUG	AAGUUCU-GAGAACUGUGGAA	Cleavage
ghr-miR396b	Gh_A08G1723	1	21	1275	1294	UUCCACAGCUUUCUUGAACUG	AAGUUCU-GAGAACUGUGGAA	Cleavage
ghr-miR398	Gh_D02G2296	1	21	2043	2063	UGUGUUCUCAGGUCACCCCUU	CAGGGGUGACUUGAGAGUAAA	Cleavage
ghr-miR398	Gh_A03G1857	1	21	2046	2066	UGUGUUCUCAGGUCACCCCUU	CGGGGGUGACUUGAGAGUAAA	Cleavage
ghr-miR398	Gh_D01G0769	1	21	513	533	UGUGUUCUCAGGUCACCCCUU	UAAGGGUGGCCCCAGAGCACA	Translation
ghr-miR399a	Gh_A10G1257	1	21	1027	1047	CGCCAAUGGAGAUUUGUCCGG	GCGCAUAGAUUGCCAUUGGCU	Translation
ghr-miR399b	Gh_A10G1257	1	21	1027	1047	CGCCAAUGGAGAUUUGUCCGG	GCGCAUAGAUUGCCAUUGGCU	Translation
ghr-miR479	Gh_A11G0376	1	22	2008	2030	CGUGAUAUUGGU-UCGGCUCAUC	GAUGAGCCGAUGCCAAUGUCACC	Cleavage
ghr-miR7484a	Gh_D08G0914	1	24	2968	2991	UUUGUAUAUUAGAUCAAAGAGCAA	GUACAUUUUGAUUUGAAAUGUGAA	Cleavage
ghr-miR7484a	Gh_A08G0768	1	24	2968	2991	UUUGUAUAUUAGAUCAAAGAGCAA	GUACAUUUUGAUUUGAAAUGUGAA	Cleavage
ghr-miR7484a	Gh_A05G3990	1	24	2860	2883	UUUGUAUAUUAGAUCAAAGAGCAA	GUACAUUUUGAUUUGAAAUGUGAA	Cleavage
ghr-miR7484a	Gh_D13G1358	1	24	795	818	UUUGUAUAUUAGAUCAAAGAGCAA	GUCAGUUUUGAUCAAAUAUAAGGA	Translation
ghr-miR7484a	Gh_A13G1093	1	24	795	818	UUUGUAUAUUAGAUCAAAGAGCAA	GUCAGUUUUGAUCAAAUAUAAGGA	Translation
ghr-miR7484a	Gh_A01G0794	1	24	3169	3192	UUUGUAUAUUAGAUCAAAGAGCAA	GUGCAUUUUGAUCUGAAAUGUGAC	Cleavage
ghr-miR7484a	Gh_A12G0439	1	24	3454	3477	UUUGUAUAUUAGAUCAAAGAGCAA	GUGCAUUUUGAUUUAAAAUGUGAC	Cleavage
ghr-miR7484a	Gh_D12G0440	1	24	3454	3477	UUUGUAUAUUAGAUCAAAGAGCAA	GUGCAUUUUGAUUUAAAAUGUGAC	Cleavage
ghr-miR7484a	Gh_D07G1556	1	24	3379	3402	UUUGUAUAUUAGAUCAAAGAGCAA	GUCCAUUUUGAUCUGAAAUGUGAU	Cleavage
ghr-miR7484a	Gh_D08G2074	1	24	3361	3384	UUUGUAUAUUAGAUCAAAGAGCAA	GUCCAUUUUGAUCUGAAAUGUGAC	Cleavage
ghr-miR7484a	Gh_A08G1723	1	24	3355	3378	UUUGUAUAUUAGAUCAAAGAGCAA	GUCCAUUUUGAUCUGAAAUGUGAC	Cleavage
ghr-miR7484a	Gh_A07G1460	1	24	3214	3237	UUUGUAUAUUAGAUCAAAGAGCAA	GUCCAUUUUGAUCUGAAAUGUGAU	Cleavage
ghr-miR7484a	Gh_D03G1084	1	24	1247	1270	UUUGUAUAUUAGAUCAAAGAGCAA	AUACAUUUGGCUUUAAAAUACAAG	Cleavage
ghr-miR7484a	Gh_A03G0454	1	24	1244	1267	UUUGUAUAUUAGAUCAAAGAGCAA	AUACAUUUGGCUUUAAAAUACAAG	Cleavage
ghr-miR7484b	Gh_D08G0914	1	24	2968	2991	UUUGUAUAUUAGAUCAAAGAGCAA	GUACAUUUUGAUUUGAAAUGUGAA	Cleavage
ghr-miR7484b	Gh_A08G0768	1	24	2968	2991	UUUGUAUAUUAGAUCAAAGAGCAA	GUACAUUUUGAUUUGAAAUGUGAA	Cleavage
ghr-miR7484b	Gh_A05G3990	1	24	2860	2883	UUUGUAUAUUAGAUCAAAGAGCAA	GUACAUUUUGAUUUGAAAUGUGAA	Cleavage
ghr-miR7484b	Gh_D13G1358	1	24	795	818	UUUGUAUAUUAGAUCAAAGAGCAA	GUCAGUUUUGAUCAAAUAUAAGGA	Translation
ghr-miR7484b	Gh_A13G1093	1	24	795	818	UUUGUAUAUUAGAUCAAAGAGCAA	GUCAGUUUUGAUCAAAUAUAAGGA	Translation
ghr-miR7484b	Gh_A01G0794	1	24	3169	3192	UUUGUAUAUUAGAUCAAAGAGCAA	GUGCAUUUUGAUCUGAAAUGUGAC	Cleavage
ghr-miR7484b	Gh_A12G0439	1	24	3454	3477	UUUGUAUAUUAGAUCAAAGAGCAA	GUGCAUUUUGAUUUAAAAUGUGAC	Cleavage
ghr-miR7484b	Gh_D12G0440	1	24	3454	3477	UUUGUAUAUUAGAUCAAAGAGCAA	GUGCAUUUUGAUUUAAAAUGUGAC	Cleavage
ghr-miR7484b	Gh_D07G1556	1	24	3379	3402	UUUGUAUAUUAGAUCAAAGAGCAA	GUCCAUUUUGAUCUGAAAUGUGAU	Cleavage
ghr-miR7484b	Gh_D08G2074	1	24	3361	3384	UUUGUAUAUUAGAUCAAAGAGCAA	GUCCAUUUUGAUCUGAAAUGUGAC	Cleavage
ghr-miR7484b	Gh_A08G1723	1	24	3355	3378	UUUGUAUAUUAGAUCAAAGAGCAA	GUCCAUUUUGAUCUGAAAUGUGAC	Cleavage

ghr-miR7484b	Gh_A07G1460	1	24	3214	3237	UUUGUAUAUUAGAUCAAAGAGCAA	GUCCAUUUUGAUCUGAAAUGUGAU	Cleavage
ghr-miR7484b	Gh_D03G1084	1	24	1247	1270	UUUGUAUAUUAGAUCAAAGAGCAA	AUACAUUUGGCUUUAAAAUACAAG	Cleavage
ghr-miR7484b	Gh_A03G0454	1	24	1244	1267	UUUGUAUAUUAGAUCAAAGAGCAA	AUACAUUUGGCUUUAAAAUACAAG	Cleavage
ghr-miR7485	Gh_D08G2074	1	24	647	670	AAAGACAUCUUUGAAUUCUUGGAG	UUAUUUCCAUUCAGAAAUGUCUUU	Cleavage
ghr-miR7485	Gh_A08G1723	1	24	641	664	AAAGACAUCUUUGAAUUCUUGGAG	UUAUUUCCAUUCAGAAAUGUCUUU	Cleavage
ghr-miR7487	Gh_D09G1795	1	24	2427	2450	AUACUCUUAUAGGACACUUGUUAA	AUGUCAAGAGCCCUUUGGGGGUAU	Translation
ghr-miR7487	Gh_D09G1795	1	24	2878	2900	AUACUCUUAUAGGACACUUGUUAA	CUAGGUAGU-UCCUGUGAGAGUAC	Cleavage
ghr-miR7487	Gh_A09G1689	1	24	2427	2450	AUACUCUUAUAGGACACUUGUUAA	AUGUCAAGAGCCCUUUGGGGGUAU	Translation
ghr-miR7488	Gh_D02G2296	1	21	561	581	UUUUGAGUACAGGGGACAAAA	GAUCGUCUCGUGUACUCAAAA	Cleavage
ghr-miR7488	Gh_D02G1209	1	21	113	133	UUUUGAGUACAGGGGACAAAA	UUUUCACCUCUGUUCUCAAGA	Cleavage
ghr-miR7488	Gh_A05G1538	1	21	2029	2049	UUUUGAGUACAGGGGACAAAA	AUUAGCUCCUUGUACCCAAAA	Cleavage
ghr-miR7488	Gh_A03G1857	1	21	561	581	UUUUGAGUACAGGGGACAAAA	GAUCGUCUCGUGCACUCAAAA	Cleavage
ghr-miR7488	Gh_D08G2074	1	21	2485	2505	UUUUGAGUACAGGGGACAAAA	GAUUCUUAUCUGUGCUCAAAC	Cleavage
ghr-miR7488	Gh_A08G1723	1	21	2479	2499	UUUUGAGUACAGGGGACAAAA	GAUUCUUAUCUGUGCUCAAAC	Cleavage
ghr-miR7489	Gh_A09G1689	1	24	1384	1406	AUUGUUGCCAAUACAGGAGAACGU	UUGGGCUCCA-UACUGGCAACAAU	Translation
ghr-miR7489	Gh_D08G1195	1	24	1383	1406	AUUGUUGCCAAUACAGGAGAACGU	AUUGGGUUCCAUAUUGACAACAAU	Cleavage
ghr-miR7489	Gh_D03G0813	1	24	1398	1421	AUUGUUGCCAAUACAGGAGAACGU	GUUGGGUUCCAUAUUGUCAACAAU	Cleavage
ghr-miR7489	Gh_A02G0949	1	24	1398	1421	AUUGUUGCCAAUACAGGAGAACGU	GUUGGGUUCCAUAUUGUCAACAAU	Cleavage
ghr-miR7489	Gh_D01G0769	1	24	937	960	AUUGUUGCCAAUACAGGAGAACGU	UCUCACUCGUCUCUUGGUGAUAAU	Cleavage
ghr-miR7489	Gh_A01G0750	1	24	937	960	AUUGUUGCCAAUACAGGAGAACGU	UCUCACUCGUCUCUUGGUGAUAAU	Cleavage
ghr-miR7489	Gh_D09G1795	1	24	1384	1406	AUUGUUGCCAAUACAGGAGAACGU	UUGGGCUCCA-UGCUGGCAACAAU	Translation
ghr-miR7491	Gh_A02G1702	1	24	622	646	UGGGAUCUUCGAGAG-GAUUGAGCC	GCCAGAAUUGCUCUUGAAGAUCUUG	Cleavage
ghr-miR7491	Gh_D03G0017	1	24	607	631	UGGGAUCUUCGAGAG-GAUUGAGCC	GCCAGAAUUGCUCUUGAAGAUCUUG	Cleavage
ghr-miR7491	Gh_A07G0531	1	24	72	95	UGGGAUCUUCGAGAGGAUUGAGCC	AGUUGAAUCAUCUCCACGGUCUCA	Translation
ghr-miR7491	Gh_D07G0600	1	24	72	95	UGGGAUCUUCGAGAGGAUUGAGCC	AGUUGAAUCAUCUCCACGGUCUCA	Translation
ghr-miR7491	Gh_D12G0368	1	24	2441	2465	UGGGAUCUU-CGAGAGGAUUGAGCC	AGCCAAAGCUUCUUGCAAGAUCCCA	Cleavage
ghr-miR7491	Gh_A12G0297	1	24	2441	2465	UGGGAUCUU-CGAGAGGAUUGAGCC	AGCCAAAGCUUCUUGCAAGAUCCCA	Cleavage
ghr-miR7494	Gh_D12G0440	1	23	1175	1197	AGCUUGUGGACUAGUUUUAACAA	CUUAUGAAUCUAGUUCACAGCCA	Cleavage
ghr-miR7494	Gh_A12G0439	1	23	1175	1197	AGCUUGUGGACUAGUUUUAACAA	CUUAUGAAUCUAGUUCACAGCCA	Cleavage
ghr-miR7494	Gh_A11G1536	1	23	446	468	AGCUUGUGGACUAGUUUUAACAA	GAGGUGAAAGUGGUGAACAAGCU	Cleavage
ghr-miR7495a	Gh_A08G1723	1	21	2411	2431	UUACUUUAGAUGUCUCCUUCA	AGAAGGAUAUAUCUAAAGAAG	Cleavage
ghr-miR7495a	Gh_A12G0439	1	21	2204	2224	UUACUUUAGAUGUCUCCUUCA	CUAAGGAGAAAUCUGAAUUGA	Cleavage
ghr-miR7495a	Gh_D12G0440	1	21	2204	2224	UUACUUUAGAUGUCUCCUUCA	CUAAGGAGAAAUCUGAAUUGA	Cleavage
ghr-miR7495a	Gh_A02G1702	1	21	404	424	UUACUUUAGAUGUCUCCUUCA	CUAGGGAGUUAUCUAAGGAAA	Cleavage
ghr-miR7495a	Gh_D03G0017	1	21	404	424	UUACUUUAGAUGUCUCCUUCA	CUAGGGAGUUAUCUAAGGAAA	Cleavage
ghr-miR7495a	Gh_D03G1095	1	21	77	97	UUACUUUAGAUGUCUCCUUCA	GAAAGGUGGAAUUUGAAGUAG	Cleavage
ghr-miR7495a	Gh_A03G0443	1	21	77	97	UUACUUUAGAUGUCUCCUUCA	GAAAGGUGGAAUUUGAAGUAG	Cleavage
ghr-miR7495a	Gh_D07G1556	1	21	2441	2461	UUACUUUAGAUGUCUCCUUCA	AAAAUGAUAUAUCUAAAGAAG	Cleavage
ghr-miR7495a	Gh_D02G1107	1	21	749	769	UUACUUUAGAUGUCUCCUUCA	CCGAGUGGACUUCUAAUGUAA	Translation
ghr-miR7495a	Gh_A02G0925	1	21	749	769	UUACUUUAGAUGUCUCCUUCA	CCGAGUGGACUUCUAAUGUAA	Translation
ghr-miR7495a	Gh_A07G0531	1	21	43	63	UUACUUUAGAUGUCUCCUUCA	ACAAGUGUACAUCUAAACUAU	Cleavage
ghr-miR7495a	Gh_D07G0600	1	21	43	63	UUACUUUAGAUGUCUCCUUCA	ACAAGUGUACAUCUAAACUAU	Cleavage
ghr-miR7495b	Gh_A08G1723	1	21	2411	2431	UUACUUUAGAUGUCUCCUUCA	AGAAGGAUAUAUCUAAAGAAG	Cleavage
ghr-miR7495b	Gh_A12G0439	1	21	2204	2224	UUACUUUAGAUGUCUCCUUCA	CUAAGGAGAAAUCUGAAUUGA	Cleavage
ghr-miR7495b	Gh_D12G0440	1	21	2204	2224	UUACUUUAGAUGUCUCCUUCA	CUAAGGAGAAAUCUGAAUUGA	Cleavage
ghr-miR7495b	Gh_A02G1702	1	21	404	424	UUACUUUAGAUGUCUCCUUCA	CUAGGGAGUUAUCUAAGGAAA	Cleavage

ghr-miR7495b	Gh_D03G0017	1	21	404	424	UUACUUUAGAUGUCUCCUUCA	CUAGGGAGUUAUCUAAGGAAA	Cleavage
ghr-miR7495b	Gh_D03G1095	1	21	77	97	UUACUUUAGAUGUCUCCUUCA	GAAAGGUGGAAUUUGAAGUAG	Cleavage
ghr-miR7495b	Gh_A03G0443	1	21	77	97	UUACUUUAGAUGUCUCCUUCA	GAAAGGUGGAAUUUGAAGUAG	Cleavage
ghr-miR7495b	Gh_D07G1556	1	21	2441	2461	UUACUUUAGAUGUCUCCUUCA	AAAAUGAUAUAUCUAAAGAAG	Cleavage
ghr-miR7495b	Gh_D02G1107	1	21	749	769	UUACUUUAGAUGUCUCCUUCA	CCGAGUGGACUUCUAAUGUAA	Translation
ghr-miR7495b	Gh_A02G0925	1	21	749	769	UUACUUUAGAUGUCUCCUUCA	CCGAGUGGACUUCUAAUGUAA	Translation
ghr-miR7495b	Gh_A07G0531	1	21	43	63	UUACUUUAGAUGUCUCCUUCA	ACAAGUGUACAUCUAAACUAU	Cleavage
ghr-miR7495b	Gh_D07G0600	1	21	43	63	UUACUUUAGAUGUCUCCUUCA	ACAAGUGUACAUCUAAACUAU	Cleavage
ghr-miR7496a	Gh_D11G0436	1	24	753	776	AUGACCAAAUUGAUAGAAUGUGUA	UUCUGAUUCUAUUGAUUUGAAUGU	Cleavage
ghr-miR7496a	Gh_A11G0376	1	24	744	767	AUGACCAAAUUGAUAGAAUGUGUA	UCCUGAUUCUAUUGAUUUGAAUGU	Cleavage
ghr-miR7496a	Gh_A05G0460	1	24	837	860	AUGACCAAAUUGAUAGAAUGUGUA	GAAUUGUACUGUUAAUUUGGUGAG	Cleavage
ghr-miR7496a	Gh_D05G3923	1	24	816	839	AUGACCAAAUUGAUAGAAUGUGUA	GAAUUGUACUGUUAAUUUGGUGAG	Cleavage
ghr-miR7496b	Gh_D11G0436	1	24	753	776	AUGACCAAAUUGAUAGAAUGUGUA	UUCUGAUUCUAUUGAUUUGAAUGU	Cleavage
ghr-miR7496b	Gh_A11G0376	1	24	744	767	AUGACCAAAUUGAUAGAAUGUGUA	UCCUGAUUCUAUUGAUUUGAAUGU	Cleavage
ghr-miR7496b	Gh_A05G0460	1	24	837	860	AUGACCAAAUUGAUAGAAUGUGUA	GAAUUGUACUGUUAAUUUGGUGAG	Cleavage
ghr-miR7496b	Gh_D05G3923	1	24	816	839	AUGACCAAAUUGAUAGAAUGUGUA	GAAUUGUACUGUUAAUUUGGUGAG	Cleavage
ghr-miR7497	Gh_A06G1787	1	23	96	117	ACAUGUGGACUGUCAUAUGGGUU	UAUACGUGU-ACGGUUCAUGUGU	Cleavage
ghr-miR7497	Gh_D06G2192	1	23	96	117	ACAUGUGGACUGUCAUAUGGGUU	UAUACGUGU-ACGGUUCAUGUGU	Cleavage
ghr-miR7498	Gh_A03G1178	1	24	488	510	AUGGUGACACAUGGUAGUCUCACA	ACUAUGGUUACCAUGU-UCACCAU	Cleavage
ghr-miR7498	Gh_D02G1615	1	24	488	510	AUGGUGACACAUGGUAGUCUCACA	ACUAUGGUUCCCAUG-GUCACCAU	Cleavage
ghr-miR7499	Gh_D05G3923	1	24	801	824	AUAUAAUUUUCGGUUAAUUCGGUU	GGCUCAAUUACCUGUGAAUUGUAC	Translation
ghr-miR7500	Gh_D08G2074	1	24	1899	1922	AUCGAGUUAUUCGAGUUAAUCGAG	UAAAAUUGAUUUGAGUAAAUCCAU	Cleavage
ghr-miR7500	Gh_A08G1723	1	24	1893	1916	AUCGAGUUAUUCGAGUUAAUCGAG	UAAAAUUGAUUUGAGUAAAUCCAU	Cleavage
ghr-miR7501	Gh_A07G0531	1	24	1159	1182	AUAUCUGAUUCUGACACGAAAAAA	CAAACUCAUGUUACAAUCAGAGAU	Translation
ghr-miR7502	Gh_D08G0914	1	24	2334	2357	UUUUUAACAGUAGAAAUGAAUGAA	ACAGUUUGUUGCUUCAGUUAAAAA	Translation
ghr-miR7502	Gh_A08G0768	1	24	2334	2357	UUUUUAACAGUAGAAAUGAAUGAA	ACAGUUUGUUGCUUCAGUUAAAAA	Translation
ghr-miR7502	Gh_D07G0509	1	24	546	569	UUUUUAACAGUAGAAAUGAAUGAA	AGCUAUUGCUGCUGCUGUUGAAGG	Cleavage
ghr-miR7502	Gh_A07G0445	1	24	546	569	UUUUUAACAGUAGAAAUGAAUGAA	AGCUAUUGCUGCUGCUGUUGAAGG	Cleavage
ghr-miR7502	Gh_D03G1084	1	24	588	611	UUUUUAACAGUAGAAAUGAAUGAA	AGCUAUUGCUGCUGCUGUUGAAGG	Cleavage
ghr-miR7502	Gh_A03G0454	1	24	588	611	UUUUUAACAGUAGAAAUGAAUGAA	AGCUAUUGCUGCUGCUGUUGAAGG	Cleavage
ghr-miR7504a	Gh_A12G0439	1	24	502	525	UAUGAAACUGUGAUUCCACGUCAU	AGUCAGGGGCAUCAAGGUUUUGUA	Translation
ghr-miR7504a	Gh_D12G0440	1	24	502	525	UAUGAAACUGUGAUUCCACGUCAU	AGUCAGGGGCAUCAAGGUUUUGUA	Translation
ghr-miR7504b	Gh_A11G1536	1	24	330	353	AGGAGGAAAAAUCUGAUUUGUCAU	UUCACGAAUCAGACUUUUCCUUUU	Translation
ghr-miR7504b	Gh_D11G1701	1	24	330	353	AGGAGGAAAAAUCUGAUUUGUCAU	UUCACGAAUCAGACUGUUCCUUUU	Translation
ghr-miR7504b	Gh_D02G0126	1	24	118	142	AGGAGG-AAAAAUCUGAUUUGUCAU	GCUACAGAUCAGAUGUUUGCCUCCU	Translation
ghr-miR7504b	Gh_A10G1257	1	24	187	210	AGGAGGAAAAAUCUGAUUUGUCAU	GCUACAGAUCAGAUGUUUGCUUCC	Translation
ghr-miR7504b	Gh_D10G1228	1	24	187	210	AGGAGGAAAAAUCUGAUUUGUCAU	GCUACAGAUCAGAUGUUUGCUUCC	Translation
ghr-miR7504b	Gh_D08G2074	1	24	1802	1824	AGGAGGAAAAAUCUGAUUUGUCAU	CCGAUAUAUU-GAUUUUUUCUCCA	Cleavage
ghr-miR7505	Gh_A08G0810	1	21	1883	1904	UUCAG-AAACCAUCCCUUCCUU	UGGGAUUGGAUGGUUUACUGAA	Cleavage
ghr-miR7505	Gh_D08G0987	1	21	1883	1904	UUCAG-AAACCAUCCCUUCCUU	UGGGAUUGGAUGGUUUACUGAA	Cleavage
ghr-miR7506	Gh_A05G3990	1	24	1266	1289	AUGUCUGGGACAUGGCGUUGGCAC	GAAUGAACACAAUGUUCUAGAUGG	Cleavage
ghr-miR7507	Gh_A01G0844	1	24	1340	1363	AAGGUAGUGAAGUAGGCAAUUGGG	GGAAGAUGCUUAAUUCACUAUCUA	Cleavage
ghr-miR7507	Gh_D01G0872	1	24	1340	1363	AAGGUAGUGAAGUAGGCAAUUGGG	GGAAGAUGCUUAAUUCACUAUCUA	Cleavage
ghr-miR7509	Gh_A05G3990	1	24	2967	2990	UCAAAAGCACUUUUUGACAGCAAU	AUUAGUUUCAGGAGGUGUUUGUGG	Cleavage
ghr-miR7509	Gh_D05G0049	1	24	2373	2396	UCAAAAGCACUUUUUGACAGCAAU	AUUAGUUUCAGGAGGUGUUUGUGG	Cleavage
ghr-miR7509	Gh_A11G0376	1	24	1986	2009	UCAAAAGCACUUUUUGACAGCAAU	UGAUAUCUCAGAAGCAGCUUUUGA	Translation

ghr-miR7510a	Gh_D10G1228	1	24	1758	1781	AAGGUCAUGAUCUUUAGCGGCGUU	UACAGCGGAAAAGAAUGUGACCUU	Translation
ghr-miR7510a	Gh_D13G1358	1	24	694	717	AAGGUCAUGAUCUUUAGCGGCGUU	AAACUUGUUUACGAUCAUGACAUU	Cleavage
ghr-miR7510a	Gh_A13G1093	1	24	694	717	AAGGUCAUGAUCUUUAGCGGCGUU	AAACUUGUUUACGAUCAUGACAUU	Cleavage
ghr-miR7510b	Gh_A09G1689	1	23	1310	1332	AAGAACAUGAUCUUUAGCGGCGU	CCGGAGAUGAUGAUUAUGUUCUU	Cleavage
ghr-miR7510b	Gh_D09G1795	1	23	1310	1332	AAGAACAUGAUCUUUAGCGGCGU	CCGGAGAUGAUGAUUAUGUUCUU	Cleavage
ghr-miR7510b	Gh_D03G0813	1	23	1325	1347	AAGAACAUGAUCUUUAGCGGCGU	CUGGAGAUGAUGAUUAUGUUCUU	Cleavage
ghr-miR7510b	Gh_A02G0949	1	23	1325	1347	AAGAACAUGAUCUUUAGCGGCGU	CUGGAGAUGAUGAUUAUGUUCUU	Cleavage
ghr-miR7511	Gh_D07G1556	1	24	1817	1840	AGAAGUUUUGCAUGUGUAGCUGAG	CAAAACUGGAUAUGCAGAACUGCU	Cleavage
ghr-miR7511	Gh_A07G1460	1	24	1649	1672	AGAAGUUUUGCAUGUGUAGCUGAG	CAAAACUGGAUAUGCAGAACUGCU	Cleavage
ghr-miR7511	Gh_D07G1556	1	24	1354	1377	AGAAGUUUUGCAUGUGUAGCUGAG	GCCAACCACACAUGCCAAGCUUAU	Cleavage
ghr-miR7511	Gh_A07G1460	1	24	1186	1209	AGAAGUUUUGCAUGUGUAGCUGAG	GCCAACCACACAUGCCAAGCUUAU	Cleavage
ghr-miR7511	Gh_D08G2074	1	24	3107	3130	AGAAGUUUUGCAUGUGUAGCUGAG	AAGAUCGACUGAUGAAAGACUUCU	Translation
ghr-miR7511	Gh_A08G1723	1	24	3101	3124	AGAAGUUUUGCAUGUGUAGCUGAG	AAGAUCGACUGAUGAAAGACUUCU	Translation
ghr-miR7512	Gh_A10G1257	1	21	2192	2212	UGCUACUUGUAGUUAUGCAUG	ACAGCAUGGCUGCAAGGAGUG	Cleavage
ghr-miR7512	Gh_D01G0769	1	21	1336	1356	UGCUACUUGUAGUUAUGCAUG	UCAGCACAACUUCAAGUGGCU	Translation
