# Supplementary material for: Map-Based Functional Analysis of the GhNLP Genes Reveals Their Roles in Enhancing Tolerance to N-Deficiency in Cotton
Source: Int J Mol Sci. 2019 Oct 8;20(19):4953. doi: 10.3390/ijms20194953 (PMC6801916; doi:10.3390/ijms20194953)
Supplement: Supplementary file 1 [file ijms-20-04953-s001.zip › Table S5.pdf]

Supplementary Table 5: miRNAs predicted to be targeting various upland cotton, *G. raimondii NLP* genes

miRNA_Acc.	Target_Acc.	miRNA_start	miRNA_end	Target_start	Target_end	miRNA_aligned_fragment	Target_aligned_fragment	Inhibition
gra-miR166d	Gorai.001G068000	1	21	1702	1722	UGAUGGGAAUGUUGUUUGGCU	UAUAGAACAGCAGUUCCAUCA	Cleavage
gra-miR166d	Gorai.004G108400	1	21	407	427	UGAUGGGAAUGUUGUUUGGCU	GCCUGAAGGAUGUUACCAUCA	Cleavage
gra-miR167a	Gorai.009G114800	1	21	675	695	UCAGAUCAUCUUGCAGCUUCA	UCAAGCUGCAAGGUUGUCUAC	Cleavage
gra-miR167b	Gorai.009G114800	1	21	675	695	UCAGAUCAUCUUGCAGCUUCA	UCAAGCUGCAAGGUUGUCUAC	Cleavage
gra-miR167c	Gorai.001G185100	1	22	2786	2807	UCAGAUGAAGCUGCCAGCAUGA	CUGGGAUGCUAGUUUCAUCUGC	Cleavage
gra-miR172a	Gorai.013G149700	1	21	1962	1982	GCGGCAUUAUCAAGAUUCACA	AAAGAAUCUCGAUACUGCCGU	Cleavage
gra-miR172a	Gorai.005G072800	1	21	1432	1451	GCGGCAUUAUCAAGAUUCACA	UCUGAAU-UUCAUAGUGCUGC	Translation
gra-miR172a	Gorai.009G012400	1	21	357	377	GCGGCAUUAUCAAGAUUCACA	UAUGGUAGUUGGUAAUGCUGU	Cleavage
gra-miR172a	Gorai.004G225000	1	21	2860	2879	GCGGCAUUAUCAAGAUUCACA	UCUGAGU-UUGAGGAUGCUGC	Cleavage
gra-miR172b	Gorai.005G072800	1	21	1432	1451	GCAGCAUUAUCAAGAUUCACA	UCUGAAU-UUCAUAGUGCUGC	Translation
gra-miR172b	Gorai.009G012400	1	21	357	377	GCAGCAUUAUCAAGAUUCACA	UAUGGUAGUUGGUAAUGCUGU	Cleavage
gra-miR172b	Gorai.004G225000	1	21	2860	2879	GCAGCAUUAUCAAGAUUCACA	UCUGAGU-UUGAGGAUGCUGC	Cleavage
gra-miR172b	Gorai.005G259100	1	21	1521	1541	GCAGCAUUAUCAAGAUUCACA	AGGGAGUCUUAAAGAUGCUGC	Translation
gra-miR172b	Gorai.008G041100	1	21	1926	1946	GCAGCAUUAUCAAGAUUCACA	AGGGAGUCUUAAAGAUGCUGC	Translation
gra-miR172b	Gorai.002G206700	1	21	1875	1895	GCAGCAUUAUCAAGAUUCACA	AGGGAGUCUUAAAGAUGCUGC	Translation
gra-miR3267	Gorai.007G043100	1	22	383	404	UCUGUCGCAGGGGAGAUGGCUG	UUUCUGACUCUCUUGCGGUGGA	Cleavage
gra-miR3476	Gorai.007G067100	1	21	676	696	UCGGACUGGAUUUGUUGACAA	UCAUCAUCAGAUCCUGUCCGU	Cleavage
gra-miR398	Gorai.005G259100	1	21	2043	2063	UGUGUUCUCAGGUCACCCCUU	CGGGGGUGACUUGAGAGUAAA	Cleavage
gra-miR398	Gorai.002G103800	1	21	513	533	UGUGUUCUCAGGUCACCCCUU	UAAGGGUGGCCCCAGAGCACA	Translation
gra-miR482	Gorai.010G148800	1	22	1359	1380	UCUUUCCAAUUCCUCCCAUUCC	GGGUGGGGAGGAAUUGGGUAGA	Cleavage
gra-miR482	Gorai.001G185100	1	22	3757	3778	UCUUUCCAAUUCCUCCCAUUCC	AUUAUUGGGGGGAUUGUGAAGA	Cleavage
gra-miR482	Gorai.004G101000	1	22	3241	3262	UCUUUCCAAUUCCUCCCAUUCC	AUAAUUGGGGGAAUUGUAAACA	Cleavage
gra-miR482	Gorai.009G006600	1	22	3151	3172	UCUUUCCAAUUCCUCCCAUUCC	AUAAUUGGAGGAAUUGUGAACA	Cleavage
gra-miR482	Gorai.002G110000	1	22	3553	3574	UCUUUCCAAUUCCUCCCAUUCC	AUUAUAGGAGGAAUUGUAAGCA	Cleavage
gra-miR482	Gorai.005G035500	1	22	3100	3121	UCUUUCCAAUUCCUCCCAUUCC	AUCAUUGGUGGAAUUGUAAACA	Cleavage
gra-miR5207	Gorai.009G348900	1	24	832	855	CUUAAGGUGGGUUUGGAUGGGCGA	CCUCCGAUCCGGGUCCACGUUGAG	Cleavage
gra-miR530a	Gorai.006G266700	1	21	2098	2118	AGGUGCAGAUGCAGUUGCAGG	GCUGUAGCUGUAACUGUAACU	Cleavage
gra-miR530a	Gorai.012G145600	1	21	1096	1116	AGGUGCAGAUGCAGUUGCAGG	CCAGCACCUGCAUCAGCAUCA	Cleavage
gra-miR530a	Gorai.009G114800	1	21	1251	1272	AGGUGCAGA-UGCAGUUGCAGG	GGUGCAACUGAAAUCUGCACUU	Translation
gra-miR530a	Gorai.009G114800	1	21	1459	1479	AGGUGCAGAUGCAGUUGCAGG	GCUGCUCGUGCAACUGCACUU	Cleavage
gra-miR530a	Gorai.003G115800	1	21	2011	2031	AGGUGCAGAUGCAGUUGCAGG	ACUCCACCUUCAUCUGUGUCU	Cleavage
gra-miR530b	Gorai.009G114800	1	21	1459	1479	AGGUGCAGGUGCAGGCGCAGC	GCUGCUCGUGCAACUGCACUU	Cleavage
gra-miR530b	Gorai.001G068000	1	21	343	363	AGGUGCAGGUGCAGGCGCAGC	GAUUCCCUUGUAUCUGUAUCU	Cleavage
gra-miR530b	Gorai.012G145600	1	21	1090	1110	AGGUGCAGGUGCAGGCGCAGC	CCAGCUCCAGCACCUGCAUCA	Cleavage
gra-miR5741	Gorai.004G101000	1	21	1310	1330	UAGUAUAAGGACUAAAUUGGU	ACCAAUUGAAUCCUUGUGUUG	Cleavage
gra-miR5741	Gorai.006G007000	1	21	1232	1252	UAGUAUAAGGACUAAAUUGGU	AUCGAUUCAGUUUUGGUACUG	Cleavage
gra-miR7484c	Gorai.009G059900	1	20	323	342	AGAAGUCGAAUUGCAUUUUG	AUGAAUGUAAUUUGGCUUUA	Cleavage
gra-miR7484c	Gorai.013G236600	1	20	293	312	AGAAGUCGAAUUGCAUUUUG	GUGAAUGUAAUUUGGCUUUA	Cleavage
gra-miR7484c	Gorai.006G206500	1	20	2647	2666	AGAAGUCGAAUUGCAUUUUG	GAAGAUAUAAUUAGAUUUCG	Cleavage
gra-miR7484g	Gorai.008G049200	1	24	3456	3479	UUCUUAUAUGUUAGAUCAAAGAGC	GCAUUUUGAUUUAAAAUGUGACAA	Translation
gra-miR7484g	Gorai.002G110000	1	24	3192	3215	UUCUUAUAUGUUAGAUCAAAGAGC	GCAUUUUGAUCUGAAAUGUGACAA	Translation
gra-miR7484g	Gorai.001G185100	1	24	3486	3509	UUCUUAUAUGUUAGAUCAAAGAGC	CCAUUUUGAUCUGAAAUGUGAUAA	Translation
gra-miR7484g	Gorai.004G225000	1	24	3357	3380	UUCUUAUAUGUUAGAUCAAAGAGC	CCAUUUUGAUCUGAAAUGUGACAA	Translation

gra-miR7484g	Gorai.013G214100	1	24	408	431	UUCUUAUAUGUUAGAUCAAAGAGC	UAGAUUGGAUUUAGCUUAUAGGGA	Cleavage
gra-miR7484g	Gorai.009G059900	1	24	405	428	UUCUUAUAUGUUAGAUCAAAGAGC	UAGAUUGGAUUUAGCUUAUAGGGA	Cleavage
gra-miR7484g	Gorai.004G101000	1	24	2970	2993	UUCUUAUAUGUUAGAUCAAAGAGC	ACAUUUUGAUUUGAAAUGUGAAAA	Translation
gra-miR7484g	Gorai.009G006600	1	24	2880	2903	UUCUUAUAUGUUAGAUCAAAGAGC	ACAUUUUGAUUUGAAAUGUGAAAA	Translation
gra-miR7484h	Gorai.006G007000	1	21	988	1008	UUUAACCGUAGAAAUGGAUGA	GCAGCAAUUUCUAUGGUUGAA	Cleavage
gra-miR7484h	Gorai.007G067100	1	21	994	1014	UUUAACCGUAGAAAUGGAUGA	GCAGCAAUUUCUAUGGUUGAA	Cleavage
gra-miR7484h	Gorai.009G114800	1	21	1725	1745	UUUAACCGUAGAAAUGGAUGA	AGAUUCAUGUCUGUGGUUGAC	Cleavage
gra-miR7484i	Gorai.006G007000	1	21	988	1008	UUUAACCGUAGAAAUGGAUGA	GCAGCAAUUUCUAUGGUUGAA	Cleavage
gra-miR7484i	Gorai.007G067100	1	21	994	1014	UUUAACCGUAGAAAUGGAUGA	GCAGCAAUUUCUAUGGUUGAA	Cleavage
gra-miR7484i	Gorai.009G114800	1	21	1725	1745	UUUAACCGUAGAAAUGGAUGA	AGAUUCAUGUCUGUGGUUGAC	Cleavage
gra-miR7484m	Gorai.004G101000	1	21	2971	2991	UUUGUAUGUUAGAUCGAAGAG	CAUUUUGAUUUGAAAUGUGAA	Cleavage
gra-miR7484m	Gorai.009G006600	1	21	2881	2901	UUUGUAUGUUAGAUCGAAGAG	CAUUUUGAUUUGAAAUGUGAA	Cleavage
gra-miR7484m	Gorai.003G120400	1	21	1250	1270	UUUGUAUGUUAGAUCGAAGAG	CAUUUGGCUUUAAAAUACAAG	Cleavage
gra-miR7484n	Gorai.006G266700	1	24	99	122	CUAGUUUGCUCUUUGAUCUAAUGU	UAUAGAUAUUAAUGAGCAAACUAG	Cleavage
gra-miR7484n	Gorai.002G103800	1	24	1603	1626	CUAGUUUGCUCUUUGAUCUAAUGU	UUGGAAGAGCAGAGAGCGAAUGAG	Cleavage
gra-miR7484n	Gorai.005G133400	1	24	358	381	CUAGUUUGCUCUUUGAUCUAAUGU	GAAGCAAAUGAAAGGGCAAGCCAG	Cleavage
gra-miR7484n	Gorai.013G236600	1	24	15	38	CUAGUUUGCUCUUUGAUCUAAUGU	UGUAAAGAACAAGAAGCAAACAGG	Translation
gra-miR7486f	Gorai.006G007000	1	24	1437	1460	CGCUUUGCUGACGUGGAAACAAAU	GGAUUUUUCCGAGUCAGCUGAGCA	Cleavage
gra-miR7486f	Gorai.002G206700	1	24	1327	1350	CGCUUUGCUGACGUGGAAACAAAU	AAAGAUUGCCAUGACAGUGAAGCA	Translation
gra-miR7486f	Gorai.005G259100	1	24	1090	1113	CGCUUUGCUGACGUGGAAACAAAU	AAGGAUUGUCAUGACAGCGAAGCA	Translation
gra-miR7486g	Gorai.006G007000	1	24	1437	1460	CGCUUUGCUGACGUGGAAACAAAU	GGAUUUUUCCGAGUCAGCUGAGCA	Cleavage
gra-miR7486g	Gorai.002G206700	1	24	1327	1350	CGCUUUGCUGACGUGGAAACAAAU	AAAGAUUGCCAUGACAGUGAAGCA	Translation
gra-miR7486g	Gorai.005G259100	1	24	1090	1113	CGCUUUGCUGACGUGGAAACAAAU	AAGGAUUGUCAUGACAGCGAAGCA	Translation
gra-miR7486i	Gorai.008G160900	1	23	51	73	GCUGACGUGGAAGGAAAUCGCUA	GGGUAAAAUCCUUCCACGUUAUC	Cleavage
gra-miR7486j	Gorai.007G046900	1	24	433	456	CAUCAGCGCGAUGUCAGGGGACGU	CUCUCCUCUGAUGUUGCUUUGAUA	Cleavage
gra-miR7492d	Gorai.004G198100	1	24	946	969	UGGGCUUAGAUUUUUUGCGGCGUU	AAUUGGGUGGAAAGUUUAAGUUCA	Cleavage
gra-miR7492d	Gorai.005G126000	1	24	1798	1821	UGGGCUUAGAUUUUUUGCGGCGUU	ACAGCAGAAAAAAAUGUGAGCUUA	Cleavage
gra-miR7492e	Gorai.007G309200	1	21	1757	1777	CCAUGAUCUUUAGCGGCGUUU	UCAUACGGCAAAAGGUUAUGG	Cleavage
gra-miR7492f	Gorai.007G309200	1	21	1757	1777	CCAUGAUCUUUAGCGGCGUUU	UCAUACGGCAAAAGGUUAUGG	Cleavage
gra-miR7492o	Gorai.006G266700	1	24	245	268	CUAAAGAUCUGAGCAUUAGUGGCG	UGCAGCGGAUGCUUAGUUUUUUAG	Cleavage
gra-miR7492o	Gorai.005G259100	1	24	429	452	CUAAAGAUCUGAGCAUUAGUGGCG	GACACCUGAUGUUCGUUUCUUUAG	Cleavage
gra-miR7493b	Gorai.010G056300	1	24	2720	2743	UUGGAUUGUUAAAAGGUUAAUUGU	CCAACUCACUUUUUAGCAAUCAAG	Cleavage
gra-miR7493b	Gorai.009G012400	1	24	2858	2881	UUGGAUUGUUAAAAGGUUAAUUGU	CAGAUUCACUCUUUAGCAAUCAAG	Cleavage
gra-miR7493b	Gorai.009G059900	1	24	430	453	UUGGAUUGUUAAAAGGUUAAUUGU	GUUUAUAAUGUUUUAACCAUUGAA	Cleavage
gra-miR7494b	Gorai.010G253900	1	23	698	720	AGAGGGAGAAGCAGAAGAGAAUA	CGGUUGUUUCUUCUUCUCCUUUU	Cleavage
gra-miR7494b	Gorai.009G348900	1	23	796	819	AGAGGGAGAAGCAGAAG-AGAAUA	GGUUCUACUUCUUCUUCUCCUUCG	Cleavage
gra-miR7494b	Gorai.004G225000	1	23	297	318	AGAGGGAGAAGCAGAAGAGAAUA	UGAUCU-UUCUGAUUUUUCUUCU	Translation
gra-miR7494b	Gorai.008G041100	1	23	191	213	AGAGGGAGAAGCAGAAGAGAAUA	CCGGCUCUAAUACUUUUCCUUUU	Cleavage
gra-miR7494c	Gorai.013G209900	1	24	514	537	AUGGAGGAAAACAGAGGGAGAAGC	CGUCCUCUUUCUGUUGUUCCCUAU	Cleavage
gra-miR7494e	Gorai.010G253900	1	21	315	335	UCGUGAUCUUUAGCGGUGUUU	GACUACCGAUGAAGAUCUCGA	Cleavage
gra-miR7494e	Gorai.007G309200	1	21	1757	1777	UCGUGAUCUUUAGCGGUGUUU	UCAUACGGCAAAAGGUUAUGG	Cleavage
gra-miR7494f	Gorai.010G253900	1	21	315	335	UCGUGAUCUUUAGCGGUGUUU	GACUACCGAUGAAGAUCUCGA	Cleavage
gra-miR7494f	Gorai.007G309200	1	21	1757	1777	UCGUGAUCUUUAGCGGUGUUU	UCAUACGGCAAAAGGUUAUGG	Cleavage
gra-miR7502b	Gorai.005G035500	1	21	2781	2801	UUGUUAAAAGUUUCAUCCAUU	ACUGGAUGCAGCUUUUGGCAU	Cleavage
gra-miR7502b	Gorai.004G225000	1	21	3309	3329	UUGUUAAAAGUUUCAUCCAUU	CAUGGGUGCAGCUUUUGGCAU	Cleavage
gra-miR7502b	Gorai.009G012400	1	21	2574	2594	UUGUUAAAAGUUUCAUCCAUU	UUUGGAUAAUAUCUUUAACAA	Cleavage

gra-miR7502b	Gorai.005G072800	1	21	525	545	UUGUUAAAAGUUUCAUCCAUU	CGUAGGUCAGACUUUUAAUAC	Cleavage
gra-miR7502b	Gorai.001G185100	1	21	3438	3458	UUGUUAAAAGUUUCAUCCAUU	CAGGGAUGCAGCUUUUGGCAU	Cleavage
gra-miR7502c	Gorai.005G035500	1	21	2781	2801	UUGUUAAAAGUUUCAUCCAUU	ACUGGAUGCAGCUUUUGGCAU	Cleavage
gra-miR7502c	Gorai.004G225000	1	21	3309	3329	UUGUUAAAAGUUUCAUCCAUU	CAUGGGUGCAGCUUUUGGCAU	Cleavage
gra-miR7502c	Gorai.009G012400	1	21	2574	2594	UUGUUAAAAGUUUCAUCCAUU	UUUGGAUAAUAUCUUUAACAA	Cleavage
gra-miR7502c	Gorai.005G072800	1	21	525	545	UUGUUAAAAGUUUCAUCCAUU	CGUAGGUCAGACUUUUAAUAC	Cleavage
gra-miR7502c	Gorai.001G185100	1	21	3438	3458	UUGUUAAAAGUUUCAUCCAUU	CAGGGAUGCAGCUUUUGGCAU	Cleavage
gra-miR7502d	Gorai.006G007000	1	24	987	1010	CUUUUAACAGUAGAAUUUGAUGGA	UGCAGCAAUUUCUAUGGUUGAAAG	Cleavage
gra-miR7502d	Gorai.007G067100	1	24	993	1016	CUUUUAACAGUAGAAUUUGAUGGA	UGCAGCAAUUUCUAUGGUUGAAAG	Cleavage
gra-miR7502d	Gorai.005G035500	1	24	2511	2534	CUUUUAACAGUAGAAUUUGAUGGA	AGGAACAGAAGUUGCUGUUAAGAG	Cleavage
gra-miR7502d	Gorai.008G041100	1	24	342	365	CUUUUAACAGUAGAAUUUGAUGGA	AACUGGAAGUUUUAUAGUUGAAGG	Cleavage
gra-miR7502d	Gorai.004G225000	1	24	3095	3118	CUUUUAACAGUAGAAUUUGAUGGA	CAGAACAAGAUCGACUGAUGAAAG	Cleavage
gra-miR7502d	Gorai.002G103800	1	24	1857	1880	CUUUUAACAGUAGAAUUUGAUGGA	AAAUCUAGAUACUGCUGUUGAGCG	Cleavage
gra-miR7502e	Gorai.006G007000	1	21	987	1007	UUAACGGUAGAAAUGGAUGAA	UGCAGCAAUUUCUAUGGUUGA	Cleavage
gra-miR7502e	Gorai.007G067100	1	21	993	1013	UUAACGGUAGAAAUGGAUGAA	UGCAGCAAUUUCUAUGGUUGA	Cleavage
gra-miR7502e	Gorai.005G035500	1	21	909	929	UUAACGGUAGAAAUGGAUGAA	UCAGUAUGUUUUUGCUGUUAA	Cleavage
gra-miR7502e	Gorai.007G184900	1	21	342	362	UUAACGGUAGAAAUGGAUGAA	ACUGUUCCUUUUUCCCGUUAG	Cleavage
gra-miR7502f	Gorai.001G185100	1	21	745	765	UUUAGCAGUAGAAAUAGAUGA	AUAUCUGUUUCUUCUGAUGAA	Cleavage
gra-miR7502f	Gorai.004G225000	1	21	757	777	UUUAGCAGUAGAAAUAGAUGA	AUAUCUGUUUCUUCUGAUGAA	Cleavage
gra-miR7502f	Gorai.005G035500	1	21	910	930	UUUAGCAGUAGAAAUAGAUGA	CAGUAUGUUUUUGCUGUUAAU	Cleavage
gra-miR7502f	Gorai.007G067100	1	21	994	1014	UUUAGCAGUAGAAAUAGAUGA	GCAGCAAUUUCUAUGGUUGAA	Cleavage
gra-miR7502f	Gorai.006G007000	1	21	988	1008	UUUAGCAGUAGAAAUAGAUGA	GCAGCAAUUUCUAUGGUUGAA	Cleavage
gra-miR7504c	Gorai.001G068000	1	24	438	461	AAGAGAAAAAAUCGGAUUUAUCAU	CUCGAGAAUCCGUUUGUUUCUUUU	Cleavage
gra-miR7504g	Gorai.013G236600	1	24	1975	1998	AGGAGGAAAUCUGAUUUGUCAUUC	AAGUGGCAGAGCAGAAUUCCUUCA	Cleavage
gra-miR7504g	Gorai.009G278600	1	24	585	608	AGGAGGAAAUCUGAUUUGUCAUUC	GGUUGCCAAUCCUGAUUUCCUCUU	Cleavage
gra-miR7504h	Gorai.007G184900	1	24	331	353	AGGAGGAAUAAGUCUGAUUUGUCA	UCACGAAUCAGACU-GUUCCUUUU	Translation
gra-miR7504h	Gorai.005G013500	1	24	544	567	AGGAGGAAUAAGUCUGAUUUGUCA	UAUAUACUCGAGCUUUUUCUUCCU	Cleavage
gra-miR7504k	Gorai.004G198100	1	24	718	741	UUUCUCCUGAUUUUUAGCAUUUUU	AACAGUAAUAAUAAUCAAGAGAAA	Cleavage
gra-miR7504l	Gorai.004G198100	1	24	718	741	UUUCUCCUGAUUUUUAGCAUUUUU	AACAGUAAUAAUAAUCAAGAGAAA	Cleavage
gra-miR7504m	Gorai.004G198100	1	24	718	741	UUUCUCCUGAUUUUUAGCAUUUUU	AACAGUAAUAAUAAUCAAGAGAAA	Cleavage
gra-miR7506b	Gorai.004G109400	1	20	107	126	CUGGGACAUGGCGUUGGCAA	UUUUCAUUGCCAUGUCUCAA	Cleavage
gra-miR7506b	Gorai.010G056300	1	20	368	387	CUGGGACAUGGCGUUGGCAA	CUGCUAAUGCCGUGCAUCAG	Cleavage
gra-miR7584e	Gorai.009G187500	1	21	2703	2723	AGGAGUUAGAUUGUAUUUUAU	GGGAAACACAAUUAGACUCCU	Cleavage
gra-miR7584e	Gorai.003G074300	1	21	2694	2714	AGGAGUUAGAUUGUAUUUUAU	GGAAGAUAUAAUCAGAUUUCG	Cleavage
gra-miR827b	Gorai.009G012400	1	21	1013	1033	UUUGUUUAUGGUCAUCUAAGC	AUUUAAAUGACAAUGGGCAGA	Translation
gra-miR827b	Gorai.002G206700	1	21	448	468	UUUGUUUAUGGUCAUCUAAGC	AUCUGGGUGCCUGUAAACAGA	Cleavage
gra-miR827b	Gorai.010G056300	1	21	1001	1021	UUUGUUUAUGGUCAUCUAAGC	AUUUGCAUGACAAUGGGCAGA	Translation
gra-miR8633	Gorai.009G187500	1	22	2778	2799	UAAGUGAAGAAAGAGGUAGGUU	CGUUUCCUGCUUUCUUCACAUA	Cleavage
gra-miR8633	Gorai.007G184900	1	22	503	523	UAAGUGAAGAAAGAGGUAGGUU	GUUCUACUUCUU-CUUCGCUUU	Translation
gra-miR8641	Gorai.006G206500	1	24	2058	2081	UUUUUACUUUGGGACACUGAUGGC	ACCUUUGGAGCCUCAAGGUGAAAA	Cleavage
gra-miR8641	Gorai.003G120400	1	24	384	406	UUUUUACUUUGGGACACUGAUGGC	GACACCAGU-UUCCAAAGUAAUGA	Cleavage
gra-miR8641	Gorai.001G058100	1	24	342	364	UUUUUACUUUGGGACACUGAUGGC	AACACCAGU-UUCUAAAGUAAUGA	Cleavage
gra-miR8646	Gorai.009G187500	1	21	2727	2747	UAGUGAGGAUGGGAAAUUUGU	ACAAGCUUCUCAUCAUCACUU	Cleavage
gra-miR8646	Gorai.002G115800	1	21	2727	2747	UAGUGAGGAUGGGAAAUUUGU	GCAAGUUUCACAUCAUCAUUU	Cleavage
gra-miR8646	Gorai.008G049200	1	21	2713	2733	UAGUGAGGAUGGGAAAUUUGU	CUGGGUUUCUCAUCUCCACUU	Cleavage
gra-miR8646	Gorai.002G110000	1	21	825	846	UAGUGAGGAUGGGAA-AUUUGU	UAGAAUGUUUCUGUUCUCACUG	Cleavage

gra-miR8647	Gorai.002G103800	1	21	1607	1627	ACCGAUUUGCUCUUUGAUCUA	AAGAGCAGAGAGCGAAUGAGU	Cleavage
gra-miR8648	Gorai.011G138700	1	21	829	849	UUUCAGCGCAGUAGAAGGAUU	UCUUGUUGUGCUGUGCUGGAA	Cleavage
gra-miR8648	Gorai.005G126000	1	21	868	888	UUUCAGCGCAGUAGAAGGAUU	UCUUGUUGUGCUGUGUUGGAA	Cleavage
gra-miR8649	Gorai.009G187500	1	24	1041	1064	ACACUGUGGAAGUGGAUCUCUCUC	CACUGAGAACUACUUUCAUUGUGU	Cleavage
gra-miR8651	Gorai.001G185100	1	24	1539	1562	UUAAUGGAUCAUAACAGCAGGUAU	AAGCCUGUUGUCAGGAUCUGUUGA	Translation
gra-miR8652	Gorai.001G185100	1	23	1468	1490	AUUAUGUGUAUGAAACUUUAGUU	CAUGAAAGGUUUAUGCACAAAGA	Cleavage
gra-miR8655	Gorai.002G206700	1	21	2608	2628	AUAUGUUCUGAUUAUAUCUGU	GAAGAUGUCAGCAGAAUGUGU	Translation
gra-miR8655	Gorai.007G043100	1	21	590	610	AUAUGUUCUGAUUAUAUCUGU	CUAUAUCUGGUCAGAAUACAU	Cleavage
gra-miR8656	Gorai.009G012400	1	21	1273	1293	UGGAAAAGUGGUAAUAAGGGG	AAUCCUGUUGCUGCUUAUCCA	Cleavage
gra-miR8657a	Gorai.007G043100	1	24	643	666	UGUAGUAAUUGUAGAAGUUCAGGG	CCGUCACUGUCUACAAUUAUCACA	Cleavage
gra-miR8657a	Gorai.003G120400	1	24	688	711	UGUAGUAAUUGUAGAAGUUCAGGG	CCCUCACUGUCUACGAUUAUAACA	Cleavage
gra-miR8657a	Gorai.001G058100	1	24	646	669	UGUAGUAAUUGUAGAAGUUCAGGG	CCCUCACUGUCUACAAUAAUUGCA	Cleavage
gra-miR8657a	Gorai.009G114800	1	24	1961	1984	UGUAGUAAUUGUAGAAGUUCAGGG	AAAGGAGUUUAUAUGAUUAUUGCU	Cleavage
gra-miR8657a	Gorai.012G145600	1	24	753	776	UGUAGUAAUUGUAGAAGUUCAGGG	UCAAGAACAUCUACAAGAAUUGCA	Cleavage
gra-miR8657a	Gorai.005G072800	1	24	1949	1972	UGUAGUAAUUGUAGAAGUUCAGGG	GCAAGAAUGUGUAUGAUUACUGCU	Cleavage
gra-miR8657b	Gorai.007G043100	1	24	643	666	UGUAGUAAUUGUAGAAGUUCAGGG	CCGUCACUGUCUACAAUUAUCACA	Cleavage
gra-miR8657b	Gorai.003G120400	1	24	688	711	UGUAGUAAUUGUAGAAGUUCAGGG	CCCUCACUGUCUACGAUUAUAACA	Cleavage
gra-miR8657b	Gorai.001G058100	1	24	646	669	UGUAGUAAUUGUAGAAGUUCAGGG	CCCUCACUGUCUACAAUAAUUGCA	Cleavage
gra-miR8657b	Gorai.009G114800	1	24	1961	1984	UGUAGUAAUUGUAGAAGUUCAGGG	AAAGGAGUUUAUAUGAUUAUUGCU	Cleavage
gra-miR8657b	Gorai.012G145600	1	24	753	776	UGUAGUAAUUGUAGAAGUUCAGGG	UCAAGAACAUCUACAAGAAUUGCA	Cleavage
gra-miR8657b	Gorai.005G072800	1	24	1949	1972	UGUAGUAAUUGUAGAAGUUCAGGG	GCAAGAAUGUGUAUGAUUACUGCU	Cleavage
gra-miR8657c	Gorai.007G043100	1	24	643	666	UGUAGUAAUUGUAGAAGUUCAGGG	CCGUCACUGUCUACAAUUAUCACA	Cleavage
gra-miR8657c	Gorai.003G120400	1	24	688	711	UGUAGUAAUUGUAGAAGUUCAGGG	CCCUCACUGUCUACGAUUAUAACA	Cleavage
gra-miR8657c	Gorai.001G058100	1	24	646	669	UGUAGUAAUUGUAGAAGUUCAGGG	CCCUCACUGUCUACAAUAAUUGCA	Cleavage
gra-miR8657c	Gorai.009G114800	1	24	1961	1984	UGUAGUAAUUGUAGAAGUUCAGGG	AAAGGAGUUUAUAUGAUUAUUGCU	Cleavage
gra-miR8657c	Gorai.012G145600	1	24	753	776	UGUAGUAAUUGUAGAAGUUCAGGG	UCAAGAACAUCUACAAGAAUUGCA	Cleavage
gra-miR8657c	Gorai.005G072800	1	24	1949	1972	UGUAGUAAUUGUAGAAGUUCAGGG	GCAAGAAUGUGUAUGAUUACUGCU	Cleavage
gra-miR8657d	Gorai.007G043100	1	24	643	666	UGUAGUAAUUGUAGAAGUUCAGGG	CCGUCACUGUCUACAAUUAUCACA	Cleavage
gra-miR8657d	Gorai.001G058100	1	24	646	669	UGUAGUAAUUGUAGAAGUUCAGGG	CCCUCACUGUCUACAAUAAUUGCA	Cleavage
gra-miR8657d	Gorai.003G120400	1	24	688	711	UGUAGUAAUUGUAGAAGUUCAGGG	CCCUCACUGUCUACGAUUAUAACA	Cleavage
gra-miR8657d	Gorai.009G114800	1	24	1961	1984	UGUAGUAAUUGUAGAAGUUCAGGG	AAAGGAGUUUAUAUGAUUAUUGCU	Cleavage
gra-miR8657d	Gorai.012G145600	1	24	753	776	UGUAGUAAUUGUAGAAGUUCAGGG	UCAAGAACAUCUACAAGAAUUGCA	Cleavage
gra-miR8657d	Gorai.005G072800	1	24	1949	1972	UGUAGUAAUUGUAGAAGUUCAGGG	GCAAGAAUGUGUAUGAUUACUGCU	Cleavage
gra-miR8657e	Gorai.007G043100	1	24	643	666	UGUAGUAAUUGUAGAAGUUCAGGG	CCGUCACUGUCUACAAUUAUCACA	Cleavage
gra-miR8657e	Gorai.001G058100	1	24	646	669	UGUAGUAAUUGUAGAAGUUCAGGG	CCCUCACUGUCUACAAUAAUUGCA	Cleavage
gra-miR8657e	Gorai.003G120400	1	24	688	711	UGUAGUAAUUGUAGAAGUUCAGGG	CCCUCACUGUCUACGAUUAUAACA	Cleavage
gra-miR8657e	Gorai.009G114800	1	24	1961	1984	UGUAGUAAUUGUAGAAGUUCAGGG	AAAGGAGUUUAUAUGAUUAUUGCU	Cleavage
gra-miR8657e	Gorai.012G145600	1	24	753	776	UGUAGUAAUUGUAGAAGUUCAGGG	UCAAGAACAUCUACAAGAAUUGCA	Cleavage
gra-miR8657e	Gorai.005G072800	1	24	1949	1972	UGUAGUAAUUGUAGAAGUUCAGGG	GCAAGAAUGUGUAUGAUUACUGCU	Cleavage
gra-miR8658	Gorai.001G185100	1	21	1417	1437	UUUGAAAAGUACAGGGACUAU	GUAUUUUUUGUAGUUUUCAAC	Cleavage
gra-miR8658	Gorai.007G046900	1	21	112	132	UUUGAAAAGUACAGGGACUAU	AAAAUUUCUGGGCUUUUCAAU	Translation
gra-miR8658	Gorai.007G309200	1	21	1402	1422	UUUGAAAAGUACAGGGACUAU	UCAAUCUCUGAAAUUUUCAAG	Translation
gra-miR8658	Gorai.013G209900	1	21	1375	1395	UUUGAAAAGUACAGGGACUAU	UCAAUCUCUGAAAUUUUCAAG	Translation
gra-miR8659a	Gorai.002G110000	1	24	1508	1531	AAUUUUUUAAGGUUGUUUGUGGAA	UGCCGCAUUUCACUUUAAAAAAUG	Cleavage
gra-miR8659a	Gorai.004G101000	1	24	1721	1744	AAUUUUUUAAGGUUGUUUGUGGAA	GUAACCCAGCAAUUUUGAAAGGUG	Cleavage
gra-miR8659b	Gorai.002G110000	1	24	1508	1531	AAUUUUUUAAGGUUGUUUGUGGAA	UGCCGCAUUUCACUUUAAAAAAUG	Cleavage

gra-miR8659b	Gorai.004G101000	1	24	1721	1744	AAUUUUUUAAGGUUGUUUGUGGAA	GUAACCCAGCAAUUUUGAAAGGUG	Cleavage
gra-miR8661	Gorai.005G035500	1	21	3168	3188	CAUUACUUUUUCAUUCAUUAA	AAAGUUAAUGGAAGAGUGUUG	Cleavage
gra-miR8661	Gorai.008G049200	1	21	1249	1270	CAUUACU-UUUUCAUUCAUUAA	CAGAUGGAUGGGAAGAGUAAUG	Cleavage
gra-miR8661	Gorai.013G214100	1	21	1066	1086	CAUUACUUUUUCAUUCAUUAA	GGAAUGAAUAAAGAGGAGGUG	Cleavage
gra-miR8661	Gorai.003G121600	1	21	577	597	CAUUACUUUUUCAUUCAUUAA	UUGACUAAUGAGAUAGUGAUG	Cleavage
gra-miR8661	Gorai.002G103800	1	21	1841	1861	CAUUACUUUUUCAUUCAUUAA	UGGAUGGUUGGAAAAGAAAUC	Cleavage
gra-miR8661	Gorai.009G012400	1	21	2201	2221	CAUUACUUUUUCAUUCAUUAA	AAAAUGAAUGUAGAACUGAUA	Translation
gra-miR8661	Gorai.010G056300	1	21	890	912	CAUUACUUUUUCAU--UCAUUAA	UGGAUGAGUAUGAGAAAGUGAUU	Cleavage
gra-miR8661	Gorai.001G185100	1	21	2254	2274	CAUUACUUUUUCAUUCAUUAA	GGUUUCAAUGGGAAAGUAAUU	Cleavage
gra-miR8661	Gorai.004G101000	1	21	3309	3329	CAUUACUUUUUCAUUCAUUAA	GGCAUUGAUGGAAAAGUGUUG	Cleavage
gra-miR8663a	Gorai.005G013500	1	24	1865	1888	UUUGAACUUGGCAACUUUUUUCAC	UUGCAAAAAGGUUCAAAGUUCAAA	Translation
gra-miR8663a	Gorai.004G101000	1	24	1803	1826	UUUGAACUUGGCAACUUUUUUCAC	UUCACAAAAGAUGCUGGGUUUUAA	Cleavage
gra-miR8663a	Gorai.013G149700	1	24	1321	1344	UUUGAACUUGGCAACUUUUUUCAC	GACAAAGCAGCUGCUAAGUUCCAG	Cleavage
gra-miR8665	Gorai.009G012400	1	24	345	368	UUAAUUAUUAUAUAGAUCAAGGAU	CAAUUUGAGCAAUAUGGUAGUUGG	Cleavage
gra-miR8667	Gorai.012G040100	1	24	552	575	UUUCCAAUAGUUUUAUGCCACAUC	AGACAAGCAUCAAACUAUUGAAGA	Cleavage
gra-miR8667	Gorai.007G309200	1	24	1675	1698	UUUCCAAUAGUUUUAUGCCACAUC	CAUAUGGCAUCAAACUUUGGGGAA	Cleavage
gra-miR8668	Gorai.001G058100	1	20	1227	1246	UUUUGGAAUCAAGACUACAU	AUUUACAUUUGGUUUCAAAA	Cleavage
gra-miR8668	Gorai.003G121600	1	20	1	19	UUUUGGAAUCAAGACUACAU	AUG-GGUCUUGAUUUCGCAA	Cleavage
gra-miR8670a	Gorai.001G185100	1	24	991	1014	AAAGUUGGGCCCCUGUUGGUGCGG	GGAUGUCAAUUGGGACCCAAUUUG	Translation
gra-miR8670a	Gorai.001G185100	1	24	1029	1052	AAAGUUGGGCCCCUGUUGGUGCGG	UUUCCCCAAAUGGUGUCCAACUUC	Translation
gra-miR8670b	Gorai.001G185100	1	24	991	1014	AAAGUUGGGCCCCUGUUGGUGCGG	GGAUGUCAAUUGGGACCCAAUUUG	Translation
gra-miR8670b	Gorai.001G185100	1	24	1029	1052	AAAGUUGGGCCCCUGUUGGUGCGG	UUUCCCCAAAUGGUGUCCAACUUC	Translation
gra-miR8671	Gorai.009G012400	1	24	1460	1483	AACUGUUCGUGAUAAGAUGUCGGU	CUCGCCAUGAUGUUAUGAACCGUU	Cleavage
gra-miR8671	Gorai.005G013500	1	24	2021	2044	AACUGUUCGUGAUAAGAUGUCGGU	AUGUACCUUUUACUAUGGGCAGUU	Cleavage
gra-miR8672	Gorai.009G114800	1	24	1964	1987	AAGAGAAAUGAUUGUAUGAAACAG	GGAGUUUAUAUGAUUAUUGCUCUG	Cleavage
gra-miR8675b	Gorai.010G253900	1	24	715	738	AGGUGAUGAUGUGGUACAAUCUCA	CCUUUUUGUUCCGCGUCAUCAGCU	Cleavage
gra-miR8675b	Gorai.010G148800	1	24	853	876	AGGUGAUGAUGUGGUACAAUCUCA	CCCUUUUGUUCCACCUCAUCAGCU	Translation
gra-miR8675b	Gorai.002G115800	1	24	2724	2747	AGGUGAUGAUGUGGUACAAUCUCA	CUUGCAAGUUUCACAUCAUCAUUU	Cleavage
gra-miR8675b	Gorai.009G174700	1	24	685	708	AGGUGAUGAUGUGGUACAAUCUCA	CCCUUUUGUUCGACGUCAUCAGCU	Cleavage
gra-miR8675c	Gorai.010G253900	1	24	715	738	AGGUGAUGAUGUGGUACAAUCUUA	CCUUUUUGUUCCGCGUCAUCAGCU	Cleavage
gra-miR8675c	Gorai.010G148800	1	24	853	876	AGGUGAUGAUGUGGUACAAUCUUA	CCCUUUUGUUCCACCUCAUCAGCU	Translation
gra-miR8675c	Gorai.002G115800	1	24	2724	2747	AGGUGAUGAUGUGGUACAAUCUUA	CUUGCAAGUUUCACAUCAUCAUUU	Cleavage
gra-miR8675c	Gorai.009G174700	1	24	685	708	AGGUGAUGAUGUGGUACAAUCUUA	CCCUUUUGUUCGACGUCAUCAGCU	Cleavage
gra-miR8676	Gorai.006G206500	1	23	1604	1626	AAGGUUGAUGGUUAAAUUUGACU	GACAGUAUUUAACCAUGAAUCUU	Cleavage
gra-miR8676	Gorai.005G126000	1	23	2526	2548	AAGGUUGAUGGUUAAAUUUGACU	UGUUGAACAUAACCACCAACCUA	Cleavage
gra-miR8676	Gorai.008G049200	1	23	2757	2779	AAGGUUGAUGGUUAAAUUUGACU	AAUUGAUUAUAGCUAUCCACCUU	Cleavage
gra-miR8676	Gorai.002G110000	1	23	2936	2958	AAGGUUGAUGGUUAAAUUUGACU	AAGAAAGAUUGACCAUUGAAUUU	Cleavage
gra-miR8677	Gorai.008G049200	1	21	738	758	AAUGAAUCUAGGUUCUCUCUU	UGGUGAGGAUCUUGAUGCAUU	Cleavage
gra-miR8678	Gorai.007G046900	1	21	398	418	AAUUUGGACUGUCACGUAGGA	CUCUACGUGGCACUCUAAGUG	Cleavage
gra-miR8678	Gorai.009G114800	1	21	1838	1858	AAUUUGGACUGUCACGUAGGA	AGGUAUGUGACAGUUUGGGUG	Cleavage
gra-miR8678	Gorai.001G185100	1	21	3144	3164	AAUUUGGACUGUCACGUAGGA	GGCUAGGUGGUGGUAUGAAUU	Cleavage
gra-miR8681	Gorai.004G225000	1	22	2897	2918	ACAUUGUUGAGGGUCUAAUCGG	AGGAUGAGACCUUCAGUGAUGC	Cleavage
gra-miR8681	Gorai.001G185100	1	22	2924	2945	ACAUUGUUGAGGGUCUAAUCGG	AGGAUGAGUCCUUCAGUGAUGC	Cleavage
gra-miR8681	Gorai.004G109400	1	22	393	413	ACAUUGUUGAGGGUCUAAUCGG	GGAAUUAG-CUCUCAGAGAUGU	Cleavage
gra-miR8683	Gorai.007G184900	1	24	571	594	AGAACUCAUAACACAUUUAGAUAA	AGCGAUGAAUGUGUUGGGAGUGCC	Cleavage
gra-miR8683	Gorai.005G035500	1	24	1255	1278	AGAACUCAUAACACAUUUAGAUAA	AUCAAUAGAUUUGUUAUGGGAACU	Cleavage

gra-miR8687	Gorai.005G259100	1	24	815	838	AGAGACAAAAGAAACACGUUCUAC	CGACCUCGGGUUCCUUUUGUUUCG	Cleavage
gra-miR8688	Gorai.002G110000	1	24	2380	2403	AGAGGAUGCUUUAUAAAACUCAUA	GCAAUGCUUUCUGAGGAAUCCUCU	Cleavage
gra-miR8689	Gorai.013G236600	1	24	810	833	AGAGGUGCUCAUGGGCUGGGUCGG	AUUUCCUAGCUCGAGAGCAGUUCU	Translation
gra-miR8689	Gorai.003G002500	1	24	194	217	AGAGGUGCUCAUGGGCUGGGUCGG	GGGUACCAGCCCACGAGCUUUUCG	Translation
gra-miR8689	Gorai.007G067100	1	23	1	23	AGAGGUGCUCAUGGGCUGGGUCG	AUGACCAGCCAAUCGGUGUCUCU	Translation
gra-miR8689	Gorai.006G007000	1	24	1491	1514	AGAGGUGCUCAUGGGCUGGGUCGG	GAGAAGCAGCGCAGGGGUACCUUU	Translation
gra-miR8693	Gorai.007G309200	1	24	276	299	AGGAUGAAAAUAUUGAUGUAGCAU	CACCACUGUCGAUGUGUUUAUCCU	Cleavage
gra-miR8694a	Gorai.006G266700	1	24	1112	1135	AGGAUGCACUGUCAGCAAAAGUAU	UAAUUUUUCUUGACAGUGUUUCUU	Cleavage
gra-miR8694a	Gorai.005G013500	1	24	1427	1450	AGGAUGCACUGUCAGCAAAAGUAU	AGUCGGUUGCUGGUGCUGCAUCUU	Cleavage
gra-miR8694b	Gorai.006G266700	1	24	1112	1135	AGGAUGCACUGUCAGCAAAAGUAU	UAAUUUUUCUUGACAGUGUUUCUU	Cleavage
gra-miR8694b	Gorai.005G013500	1	24	1427	1450	AGGAUGCACUGUCAGCAAAAGUAU	AGUCGGUUGCUGGUGCUGCAUCUU	Cleavage
gra-miR8695	Gorai.005G133400	1	21	534	554	AGGAUGUAAAAGAAUAGGUGA	AAAUCUAUUUUAUUAUAUUUU	Translation
gra-miR8695	Gorai.012G016100	1	21	330	350	AGGAUGUAAAAGAAUAGGUGA	AAAUCUAUUUUAUUAUAUUUU	Translation
gra-miR8698	Gorai.013G149700	1	24	1577	1600	AGGGACAAUUAACUUUAACGGUCA	AAAUGGCUAAACUUAAUUGGUCUU	Cleavage
gra-miR8699	Gorai.004G198100	1	24	887	910	AGGGCCAUUUUGACAAAACAUGCA	AAUAUGGUCUUUCAAGAUGGCCCC	Cleavage
gra-miR8699	Gorai.005G013500	1	24	1010	1033	AGGGCCAUUUUGACAAAACAUGCA	AACACGGUAUCUCAAGAUGGCCGU	Cleavage
gra-miR8702	Gorai.002G110000	1	24	1702	1725	AGGUAUUGUCUCUGGGGAAGGGUU	GUUUAUUAUUCAGAAAGAAUACCU	Translation
gra-miR8702	Gorai.005G035500	1	24	2006	2029	AGGUAUUGUCUCUGGGGAAGGGUU	CUAAUUUGACCACAGACAGUGCUU	Cleavage
gra-miR8704	Gorai.002G103800	1	24	1063	1086	AGUCAGUUCAGGCAGUCAGACCGU	GAUGAUUGGCUGUUUGAGUUUGCU	Cleavage
gra-miR8706a	Gorai.006G206500	1	24	2263	2286	AGUUUUAGGAUUGAUUUGAUGAAA	CUGUGCCAAGGUAAUCCUGAAAUU	Cleavage
gra-miR8706a	Gorai.009G187500	1	24	936	959	AGUUUUAGGAUUGAUUUGAUGAAA	UGAGAUCAAAGAAGUUCUAAAACG	Cleavage
gra-miR8706a	Gorai.013G236600	1	24	1500	1524	AGUUUUAGGAUUGAU-UUGAUGAAA	UAGCAACAAUGUUAAUCUUGAAACU	Cleavage
gra-miR8706b	Gorai.006G206500	1	24	2263	2286	AGUUUUAGGAUUGAUUUGAUGAAA	CUGUGCCAAGGUAAUCCUGAAAUU	Cleavage
gra-miR8706b	Gorai.009G187500	1	24	936	959	AGUUUUAGGAUUGAUUUGAUGAAA	UGAGAUCAAAGAAGUUCUAAAACG	Cleavage
gra-miR8706b	Gorai.013G236600	1	24	1500	1524	AGUUUUAGGAUUGAU-UUGAUGAAA	UAGCAACAAUGUUAAUCUUGAAACU	Cleavage
gra-miR8707	Gorai.013G236600	1	24	537	560	AUAAAAACUUUUGAAUAAUUCAGU	UCCUAGUGCUUCAAAAGUUGUUAA	Cleavage
gra-miR8707	Gorai.006G266900	1	24	142	165	AUAAAAACUUUUGAAUAAUUCAGU	GCGCCAUUGAUGAACAGUUUUUGU	Translation
gra-miR8708	Gorai.007G309200	1	23	2156	2178	AGGAGGAGUUAUGGAUAGUUUUA	CUCUAGUGACUGUAACUCCUCCU	Cleavage
gra-miR8708	Gorai.006G266700	1	23	2100	2124	AGGAGGAGUUAUGG--AUAGUUUUA	UGUAGCUGUAACUGUAACUCCUCCU	Cleavage
gra-miR8709a	Gorai.010G253900	1	24	105	128	AUAUUAAAACUAUACAUGAACUUU	ACGGUUCAUGUGUAGUUUCGGUGG	Cleavage
gra-miR8709a	Gorai.001G185100	1	24	1065	1088	AUAUUAAAACUAUACAUGAACUUU	GGACACUAUGGGUGGUUUUAAUGC	Cleavage
gra-miR8709b	Gorai.007G067100	1	24	1418	1441	UCAUGUAUAAUUUUGAGAUUUGUC	UAAAGGUUUUAAGAUUGUAUCUGG	Cleavage
gra-miR8709c	Gorai.012G145600	1	20	119	138	AUACAUGAACUUCGAUUUAA	AUAAGGCUAAGUUUAUGUGC	Cleavage
gra-miR8712	Gorai.006G266700	1	24	843	866	AUAUCAUUGGUGAUGUAUCGUCUU	GCCAAAAGAUAUUGCCGAUGAUAU	Cleavage
gra-miR8712	Gorai.009G114800	1	24	837	860	AUAUCAUUGGUGAUGUAUCGUCUU	ACCGAGAGAAAUUAUUAAUGAAAU	Cleavage
gra-miR8717	Gorai.009G114800	1	24	1764	1787	AUUGGUUGUUCUGAUUCGAGGCUA	GGAGUUCAAAUUAGAACAACAUAU	Cleavage
gra-miR8717	Gorai.010G056300	1	24	2283	2306	AUUGGUUGUUCUGAUUCGAGGCUA	CACUCAUGCUUUAGAACAGCCAGU	Cleavage
gra-miR8718	Gorai.002G110000	1	21	2966	2986	AUUUUGGAAGAAUUUCAGCUG	AAGCUGACAUUCUCUCAAAAC	Cleavage
gra-miR8718	Gorai.008G049200	1	21	3230	3250	AUUUUGGAAGAAUUUCAGCUG	AAGCUGACAUCCUUUCAAAGC	Translation
gra-miR8718	Gorai.013G209900	1	21	859	879	AUUUUGGAAGAAUUUCAGCUG	AAGUUGAAAAUUUUUCCAGAU	Cleavage
gra-miR8718	Gorai.003G115800	1	21	608	628	AUUUUGGAAGAAUUUCAGCUG	CAACUGGUGUUUUACCAAAAU	Cleavage
gra-miR8718	Gorai.003G002500	1	21	617	637	AUUUUGGAAGAAUUUCAGCUG	CUCUUGAAGAUCUUGCAAAAU	Cleavage
gra-miR8718	Gorai.007G067100	1	21	36	56	AUUUUGGAAGAAUUUCAGCUG	CCGCCGGAAUUCUUAUAGAAG	Cleavage
gra-miR8720	Gorai.001G058100	1	24	1455	1478	CAGUGUAGUUCUAAACCCGUCGGG	UUUGAAGGGUUUAAGAUUACAUUU	Translation
gra-miR8720	Gorai.003G120400	1	24	1473	1496	CAGUGUAGUUCUAAACCCGUCGGG	UUGGAAGGGUUUGAAACUGCAUUU	Translation
gra-miR8721a	Gorai.003G115800	1	21	53	73	CAUCGAUAGUUUGAGGAUGUA	UCAAUGCUCGAAUUGUUGAUG	Cleavage

gra-miR8721a	Gorai.002G218300	1	21	768	788	CAUCGAUAGUUUGAGGAUGUA	AACAUCCCCAGAUUAUCGGAC	Cleavage
gra-miR8721a	Gorai.009G278600	1	21	536	556	CAUCGAUAGUUUGAGGAUGUA	UGCAGGUUCAGAAUAUUGAUG	Cleavage
gra-miR8721a	Gorai.012G040100	1	21	554	574	CAUCGAUAGUUUGAGGAUGUA	ACAAGCAUCAAACUAUUGAAG	Cleavage
gra-miR8721b	Gorai.003G115800	1	21	53	73	CAUCGAUAGUUUGAGGAUGUA	UCAAUGCUCGAAUUGUUGAUG	Cleavage
gra-miR8721b	Gorai.002G218300	1	21	768	788	CAUCGAUAGUUUGAGGAUGUA	AACAUCCCCAGAUUAUCGGAC	Cleavage
gra-miR8721b	Gorai.009G278600	1	21	536	556	CAUCGAUAGUUUGAGGAUGUA	UGCAGGUUCAGAAUAUUGAUG	Cleavage
gra-miR8721b	Gorai.012G040100	1	21	554	574	CAUCGAUAGUUUGAGGAUGUA	ACAAGCAUCAAACUAUUGAAG	Cleavage
gra-miR8722	Gorai.009G006600	1	22	2158	2179	CAUGUUUUUCCUGUUCAUCUUC	GGUGAUGACCAGGCAAAACAUG	Cleavage
gra-miR8722	Gorai.004G101000	1	22	2248	2269	CAUGUUUUUCCUGUUCAUCUUC	GAUGGUGAGCAGGCAAAACCUG	Cleavage
gra-miR8722	Gorai.008G049200	1	22	28	49	CAUGUUUUUCCUGUUCAUCUUC	AUGGAUCAACAGAAGAACCAUG	Translation
gra-miR8722	Gorai.005G126000	1	22	1720	1741	CAUGUUUUUCCUGUUCAUCUUC	AGUGAUGGGAAGGAAAAGGAUG	Cleavage
gra-miR8722	Gorai.004G109400	1	22	660	681	CAUGUUUUUCCUGUUCAUCUUC	GAAGAAGAACAAGAAAAAGAUC	Translation
gra-miR8723a	Gorai.009G278600	1	23	336	357	UGUAACAGUAAGCUGACGUGACA	UGACGCGUU-GAUUACUGUUACG	Cleavage
gra-miR8723a	Gorai.001G185100	1	23	1626	1649	UGU-AACAGUAAGCUGACGUGACA	AGGGAGGUCUGCUUACUGUUCACA	Cleavage
gra-miR8723b	Gorai.004G108400	1	24	504	527	CCAUUAACGGUGUAACAGUAAGCU	UGCUGGCCGUAACAUUGUUGAUGG	Cleavage
gra-miR8723b	Gorai.006G266700	1	24	267	290	CCAUUAACGGUGUAACAGUAAGCU	AGGCGACUCUCUCACCGUUGAUGU	Cleavage
gra-miR8726	Gorai.004G109400	1	21	509	529	CGAUGGAGUCUGGAGACAAAA	AAUUGCCUCUAGAGUCCGUUG	Cleavage
gra-miR8726	Gorai.009G348900	1	21	1202	1225	CGAUGGAGUCUGGAGA---CAAAA	AUUUGGCUUCUCCAGAUUCCAUCU	Cleavage
gra-miR8726	Gorai.007G046900	1	21	328	348	CGAUGGAGUCUGGAGACAAAA	CCGUCUUUUCAGCCUCCAUCG	Cleavage
gra-miR8728	Gorai.004G225000	1	24	578	601	CGGGCUUGGGCAAAAUUUUAGGCU	GUGGAAAAAUAUUGCCCAGGCCUA	Cleavage
gra-miR8728	Gorai.002G218300	1	24	83	106	CGGGCUUGGGCAAAAUUUUAGGCU	GCGGGAAAAUAUUGCCCAGACCUG	Cleavage
gra-miR8728	Gorai.001G185100	1	24	566	589	CGGGCUUGGGCAAAAUUUUAGGCU	GUGGAAAAAUAUUGCCUAGGCCAA	Cleavage
gra-miR8728	Gorai.002G115800	1	24	836	859	CGGGCUUGGGCAAAAUUUUAGGCU	AAAUCAAGAUUCAGCCUGAGCUCG	Cleavage
gra-miR8730	Gorai.010G056300	1	24	1433	1456	CUAAGAGAUUGGGAUUUGGUAGGA	AGUUCUCAAAUCCUAAGUUCCUGG	Cleavage
gra-miR8730	Gorai.009G226800	1	24	581	604	CUAAGAGAUUGGGAUUUGGUAGGA	UGGCGGCAAAUCCCGAUUUUCUAU	Cleavage
gra-miR8731	Gorai.006G266700	1	21	1046	1066	CUAUAAACAGUCGAUGGUAUC	GCUUCCAUCGACUGUUUGUCU	Cleavage
gra-miR8731	Gorai.005G072800	1	21	656	676	CUAUAAACAGUCGAUGGUAUC	GGUGUCCUUGGUUCUUUAUAG	Cleavage
gra-miR8733	Gorai.007G309200	1	21	956	976	GAGCUUGGAAGUGCAUCCGGC	AACUGAACUACUUUCAGGCUU	Cleavage
gra-miR8734	Gorai.009G012400	1	24	1403	1426	GAUCAUAUCUCGUACGUUAGGACA	CACCGCAGCAAGUGAGAUAUGAUU	Cleavage
gra-miR8735	Gorai.009G006600	1	24	2300	2322	GGGGACAAUACCUUCGAUUGUUGG	CCAA-AGUUGAAGGCGUUGCCCCC	Translation
gra-miR8735	Gorai.009G012400	1	24	3562	3585	GGGGACAAUACCUUCGAUUGUUGG	GUGGCAUUCUAUGGUGUUGUUCUC	Cleavage
gra-miR8736	Gorai.005G126000	1	24	244	267	GUGGAACGUGUUAAGAGAGGAAUA	UAUACCUCUUUUGACACGUUACAU	Cleavage
gra-miR8736	Gorai.003G115800	1	24	1036	1059	GUGGAACGUGUUAAGAGAGGAAUA	AAUGUCUUGUUUGGUACAUUCCAC	Cleavage
gra-miR8737	Gorai.004G108400	1	24	1764	1787	GUGUAUCUCCUGAAAACGACGACA	ACCUGGUGGUUUUAGGAUGUACAC	Cleavage
gra-miR8738a	Gorai.009G059900	1	23	1004	1026	GUUAACUUUAACGGUCAACGGUU	GGUUCUUUGUUGUUGAAGUUAGU	Cleavage
gra-miR8738b	Gorai.009G059900	1	23	1004	1026	GUUAACUUUAACGGUCAACGGUU	GGUUCUUUGUUGUUGAAGUUAGU	Cleavage
gra-miR8739	Gorai.005G035500	1	24	1958	1981	UAACUAAAUAGUGACACGUGGCAU	CUGUUACACCUCACAAUUUAGUUG	Translation
gra-miR8742a	Gorai.005G072800	1	21	700	720	UAUCUUAUUCAUCUUGGACUG	UUGUUCAGGAUUAAGAAGAUG	Translation
gra-miR8742a	Gorai.006G206500	1	21	2066	2086	UAUCUUAUUCAUCUUGGACUG	AGCCUCAAGGUGAAAAAGAUG	Cleavage
gra-miR8742a	Gorai.004G109400	1	21	2143	2163	UAUCUUAUUCAUCUUGGACUG	GGGUUUAAGAUUGACGAGAUA	Translation
gra-miR8742a	Gorai.013G214100	1	21	2035	2055	UAUCUUAUUCAUCUUGGACUG	GGAUUCAAGAUUGAUGAGAUU	Translation
gra-miR8742a	Gorai.008G160900	1	21	151	171	UAUCUUAUUCAUCUUGGACUG	UCGUUGAAGAUGGGGGAGAUG	Cleavage
gra-miR8742b	Gorai.005G072800	1	21	700	720	UAUCUUAUUCAUCUUGGACUG	UUGUUCAGGAUUAAGAAGAUG	Translation
gra-miR8742b	Gorai.006G206500	1	21	2066	2086	UAUCUUAUUCAUCUUGGACUG	AGCCUCAAGGUGAAAAAGAUG	Cleavage
gra-miR8742b	Gorai.004G109400	1	21	2143	2163	UAUCUUAUUCAUCUUGGACUG	GGGUUUAAGAUUGACGAGAUA	Translation
gra-miR8742b	Gorai.013G214100	1	21	2035	2055	UAUCUUAUUCAUCUUGGACUG	GGAUUCAAGAUUGAUGAGAUU	Translation

gra-miR8742b	Gorai.008G160900	1	21	151	171	UAUCUUAUUCAUCUUGGACUG	UCGUUGAAGAUGGGGGAGAUG	Cleavage
gra-miR8743b	Gorai.007G184900	1	24	602	625	UUUGGAAAGUUAUAAAAUGGUCAU	AUGUGCAGUCUGGAACUUUUCAAG	Cleavage
gra-miR8745	Gorai.004G109400	1	21	510	530	UCAACGGAGUUGGGAGACAAA	AUUGCCUCUAGAGUCCGUUGA	Cleavage
gra-miR8746	Gorai.001G185100	1	21	371	390	UCCAUAUUUCACUAUCUCUUA	AGAAAGAUGGUGAAAU-UGGA	Cleavage
gra-miR8746	Gorai.004G225000	1	21	332	351	UCCAUAUUUCACUAUCUCUUA	UUAGAGAGAGUGAAA-AUGGG	Cleavage
gra-miR8746	Gorai.004G101000	1	21	962	982	UCCAUAUUUCACUAUCUCUUA	UUUCAGAUAAUGUAAUAUGGA	Cleavage
gra-miR8748	Gorai.007G046900	1	24	818	841	UCGGUGGAGAUGGAUAAAAUGAAU	UGGCUUCUGAACCAUCUUCAUCGA	Cleavage
gra-miR8748	Gorai.001G185100	1	24	738	761	UCGGUGGAGAUGGAUAAAAUGAAU	UGCCCUUAUAUCUGUUUCUUCUGA	Cleavage
gra-miR8748	Gorai.004G225000	1	24	750	773	UCGGUGGAGAUGGAUAAAAUGAAU	UGCCCUUAUAUCUGUUUCUUCUGA	Cleavage
gra-miR8748	Gorai.003G115800	1	24	782	805	UCGGUGGAGAUGGAUAAAAUGAAU	UGCCCUCUGGACCAUCUGCACCGA	Cleavage
gra-miR8750	Gorai.013G236600	1	24	211	234	UCUUAGUUGGCAUAUACUCAAGGA	CACAUGGAUGUAUGCCAUCUAAGG	Cleavage
gra-miR8750	Gorai.002G110000	1	24	494	517	UCUUAGUUGGCAUAUACUCAAGGA	GACUUCUAUAUAUGUCAAUUGGGA	Cleavage
gra-miR8750	Gorai.006G266700	1	24	464	487	UCUUAGUUGGCAUAUACUCAAGGA	CUAUAGACUGUAUGCCUAUUAAUA	Cleavage
gra-miR8751a	Gorai.002G115800	1	24	2713	2736	UGAAAUUUGUAGAGACAAAACGCU	AUUAAGUUGUCCUUGCAAGUUUCA	Cleavage
gra-miR8751a	Gorai.001G185100	1	24	1700	1723	UGAAAUUUGUAGAGACAAAACGCU	CUUCCCUUGUUUUUUCAAAUUCCA	Translation
gra-miR8751a	Gorai.013G214100	1	24	331	354	UGAAAUUUGUAGAGACAAAACGCU	UGUAAUUUGGCUCUGGAGGUUUCU	Cleavage
gra-miR8751a	Gorai.009G059900	1	24	328	351	UGAAAUUUGUAGAGACAAAACGCU	UGUAAUUUGGCUUUAGAGGUUUCU	Cleavage
gra-miR8751b	Gorai.005G072800	1	24	1053	1076	UGAAAUUUGUAGAGAGAUAACGCU	CCAUGCAUCGCUCUACGGAUUCCA	Cleavage
gra-miR8755	Gorai.002G206700	1	24	2608	2631	UGGACGCGCUUUGCUGACGUGGCA	GAAGAUGUCAGCAGAAUGUGUCUC	Cleavage
gra-miR8756	Gorai.002G115800	1	24	2254	2278	UGGAC-UGUUAAAAUUUUAAUGGCA	AGACCUUCAAAUUUUAACAUGUCUG	Cleavage
gra-miR8757b	Gorai.013G236600	1	23	1839	1861	UGGGUCGAGCUUAGGCAAGCAUA	UGAACUUGCUGGAGCUUCACCUA	Cleavage
gra-miR8758	Gorai.002G115800	1	24	1846	1869	UGGGCUUCUUGCAAGAUGAAGGUA	CGCCAAUAUUUUGCUGGAAGCCUA	Translation
gra-miR8758	Gorai.003G074300	1	24	1780	1803	UGGGCUUCUUGCAAGAUGAAGGUA	CAACAGUAUUUUGCAGGGAGUCUC	Cleavage
gra-miR8758	Gorai.006G206500	1	24	1762	1785	UGGGCUUCUUGCAAGAUGAAGGUA	CAACAGUAUUUUGCAGGGAGUCUC	Cleavage
gra-miR8759	Gorai.010G148800	1	21	1140	1160	UGGUGGAAGUAUUGUGCCCGG	CCGGCCACAGUAUGUCCAGCA	Cleavage
gra-miR8759	Gorai.004G131700	1	21	236	256	UGGUGGAAGUAUUGUGCCCGG	CUGGUUACAAUCUUUUCGUCA	Translation
gra-miR8759	Gorai.004G101000	1	21	710	729	UGGUGGAAGUAUUGUGCCCGG	CCGGGCA-ACUACUGCCACCA	Cleavage
gra-miR8759	Gorai.008G049200	1	21	2285	2305	UGGUGGAAGUAUUGUGCCCGG	CAGGGCAAAGAACUUCUGUUA	Translation
gra-miR8760	Gorai.009G226800	1	24	639	662	UGUGACAUCGUCAAAUUCGGCCAU	UCCUCCGGUUGUGACGGUGGCACA	Cleavage
gra-miR8760	Gorai.004G225000	1	24	2857	2880	UGUGACAUCGUCAAAUUCGGCCAU	GAAUCUGAGUUUGAGGAUGCUGCA	Translation
gra-miR8761	Gorai.002G103800	1	24	1318	1341	UGUUGACGUUGCAUACAUGUGGAU	GCUAGCAAGGAGGUAAUGUCAGCA	Cleavage
gra-miR8761	Gorai.007G067100	1	24	1114	1137	UGUUGACGUUGCAUACAUGUGGAU	AGUGAAAUGUCUGCAAUCUUGACA	Cleavage
gra-miR8762a	Gorai.001G185100	1	24	3630	3653	UUAACAUUUGUUAACUUUGCUGAC	GGCACCAGAGUUAUUAAAUGGUAA	Translation
gra-miR8762a	Gorai.003G115800	1	24	618	641	UUAACAUUUGUUAACUUUGCUGAC	UUUACCAAAAUUUACAGCUGUUGA	Cleavage
gra-miR8762b	Gorai.001G185100	1	24	3630	3653	UUAACGUUUGUUAACUUUGUUGAU	GGCACCAGAGUUAUUAAAUGGUAA	Translation
gra-miR8762c	Gorai.001G185100	1	24	3630	3653	UUAACGUUUGUUAACUUUGUUGAU	GGCACCAGAGUUAUUAAAUGGUAA	Translation
gra-miR8762d	Gorai.013G214100	1	24	711	734	UUGUUAACUUUGAUGAUGUGGCAU	GGUUAACGUUGACAAAGUUAAUGA	Cleavage
gra-miR8762d	Gorai.003G074300	1	24	1887	1910	UUGUUAACUUUGAUGAUGUGGCAU	UCGUAAGAUCAACAAAGUUAACCG	Cleavage
gra-miR8762d	Gorai.009G114800	1	24	1779	1802	UUGUUAACUUUGAUGAUGUGGCAU	ACAACAUAUUCUUAAAGCUAACAU	Cleavage
gra-miR8762d	Gorai.009G114800	1	24	1571	1593	UUGUUAACUUUGAUGAUGUGGCAU	AUGC-ACUUUUUCAAGGUUCACGA	Cleavage
gra-miR8762e	Gorai.002G206700	1	24	2514	2537	UUUUUAACUUUACUGACAUGGCAU	AGCAACGUUCGGUGAAGUUAAAAU	Cleavage
gra-miR8762e	Gorai.004G109400	1	24	765	788	UUUUUAACUUUACUGACAUGGCAU	GAAGAAGAUCAGUAAAGCUAAAGA	Cleavage
gra-miR8762e	Gorai.005G126000	1	24	768	791	UUUUUAACUUUACUGACAUGGCAU	AAUCCAUUACAGUAAGGUUGAGUA	Cleavage
gra-miR8762e	Gorai.009G278600	1	24	198	221	UUUUUAACUUUACUGACAUGGCAU	UGCUUAUGUCGGUGGAGAUACGAA	Cleavage
gra-miR8762e	Gorai.002G103800	1	24	1586	1609	UUUUUAACUUUACUGACAUGGCAU	CAGAAAUGUGGGAGAAGUUGGAAG	Cleavage
gra-miR8762e	Gorai.004G101000	1	24	2112	2134	UUUUUAACUUUACUGACAUGGCAU	AAAUAAUG-CAGUGAAGGUGGAAA	Cleavage

gra-miR8763	Gorai.006G266700	1	23	542	563	UUAAUACUGUUAAAUUUGUUGGU	ACCAA-AGAUUUAAUAGUUUUAA	Cleavage
gra-miR8763	Gorai.006G266900	1	23	378	400	UUAAUACUGUUAAAUUUGUUGGU	UAAGGUAGGGUUAACAUUAUUGA	Cleavage
gra-miR8766	Gorai.005G072800	1	24	1059	1082	UUAUUUUGGAAUUAGAAAAGUCGU	AUCGCUCUACGGAUUCCAGAAUGG	Cleavage
gra-miR8767c	Gorai.003G120400	1	21	1468	1488	UUUUCAACUCUGCCAAGCAAU	GUUGGUUGGAAGGGUUUGAAA	Cleavage
gra-miR8767c	Gorai.007G043100	1	21	1423	1443	UUUUCAACUCUGCCAAGCAAU	CUUGGUUGGAAGGGUUUGAAA	Cleavage
gra-miR8768	Gorai.009G174700	1	21	151	171	UUCCAUGUCACAGAGAUGUUG	AAUCAGCUCUGUUACAUGGGC	Cleavage
gra-miR8769	Gorai.009G012400	1	24	482	505	UUGAACUUUGACCGGAUCUAGGGA	CAACAGGUUUUGGACAGAGUUCAA	Translation
gra-miR8769	Gorai.009G012400	1	24	2668	2691	UUGAACUUUGACCGGAUCUAGGGA	AACUUGGAUUCCCUUGAAGUUCAA	Cleavage
gra-miR8770	Gorai.001G068000	1	24	398	421	UUGAUGGUGGUAAGAAAUGUGCAU	AUGAUCGUUUAAUAGCACCAUCAG	Translation
gra-miR8771a	Gorai.004G109400	1	21	1518	1538	GGAUUGUCGUUAGGGAGGUAA	AUACUUCCCUGAAGAUGGUUC	Cleavage
gra-miR8771a	Gorai.012G040100	1	21	582	602	GGAUUGUCGUUAGGGAGGUAA	UUACAUGGCUAAUGACAAACC	Cleavage
gra-miR8771a	Gorai.005G259100	1	21	1740	1760	GGAUUGUCGUUAGGGAGGUAA	AAACUUUUCUAACAACGUUCC	Cleavage
gra-miR8771a	Gorai.009G012400	1	21	834	854	GGAUUGUCGUUAGGGAGGUAA	CCAGCUUCCUGAUGAGGAUCU	Cleavage
gra-miR8771b	Gorai.004G109400	1	21	1518	1538	GGAUUGUCGUUAGGGAGGUAA	AUACUUCCCUGAAGAUGGUUC	Cleavage
gra-miR8771b	Gorai.012G040100	1	21	582	602	GGAUUGUCGUUAGGGAGGUAA	UUACAUGGCUAAUGACAAACC	Cleavage
gra-miR8771b	Gorai.005G259100	1	21	1740	1760	GGAUUGUCGUUAGGGAGGUAA	AAACUUUUCUAACAACGUUCC	Cleavage
gra-miR8771b	Gorai.009G012400	1	21	834	854	GGAUUGUCGUUAGGGAGGUAA	CCAGCUUCCUGAUGAGGAUCU	Cleavage
gra-miR8771c	Gorai.003G002500	1	24	482	505	UAGGGAGGUAACGAAGCUUAACGG	AUCAGGAUCUUAAUUGCCUCCCUA	Cleavage
gra-miR8771d	Gorai.002G110000	1	21	1798	1818	UGCCAUGUAGGAUUGUCGUUA	CAUCCACAAUCUGAUAUGGCA	Cleavage
gra-miR8771d	Gorai.004G225000	1	21	1940	1960	UGCCAUGUAGGAUUGUCGUUA	AUACUACAAUCUUGGAUGCCA	Cleavage
gra-miR8771e	Gorai.007G184900	1	24	571	594	GGCAUUUAAAACACAUUUGGACUG	AGCGAUGAAUGUGUUGGGAGUGCC	Cleavage
gra-miR8771f	Gorai.006G007000	1	23	953	975	UUGGACAUCCAAGUUAGCAUUUA	UUGCUGCUUGUUUGGAUGUUCUG	Cleavage
gra-miR8771f	Gorai.001G185100	1	23	1973	1994	UUGGACAUCCAAGUUAGCAUUUA	AUACUGCAAGCUUGGAUG-CCAG	Cleavage
gra-miR8772	Gorai.013G236600	1	21	1146	1166	UUGGACUGUGGCUACAUAUAG	UGGUAUGAAGCCAUAUUCCGA	Cleavage
gra-miR8773	Gorai.008G049200	1	23	1930	1952	UUGGAUGAACGGUGCGUUUACUU	GCAGAAAUGAACCGUUUAUCAAA	Cleavage
gra-miR8773	Gorai.009G012400	1	23	4096	4118	UUGGAUGAACGGUGCGUUUACUU	AUGGAGAGGUGCUGGUCAUCCGA	Cleavage
gra-miR8774	Gorai.009G006600	1	24	2352	2375	UUGGAUUUUGAUUCAUAGAUUCGU	AGCUGCCGAUGAAUCAAAAUCAAU	Cleavage
gra-miR8774	Gorai.006G206500	1	24	266	289	UUGGAUUUUGAUUCAUAGAUUCGU	CCAAUUCAGUGAAUGAAAAUCCUA	Translation
gra-miR8774	Gorai.013G149700	1	24	1730	1753	UUGGAUUUUGAUUCAUAGAUUCGU	AUGAGUUGAAGGAUCCAAAUUCAA	Cleavage
gra-miR8774	Gorai.004G225000	1	24	2627	2650	UUGGAUUUUGAUUCAUAGAUUCGU	UAAGAUCUGUGCAUGGGAAUUCAA	Translation
gra-miR8774	Gorai.009G226800	1	24	825	848	UUGGAUUUUGAUUCAUAGAUUCGU	AAAAAUCGAUGAAUCAAAUGCUAG	Cleavage
gra-miR8776a	Gorai.009G006600	1	24	484	507	UUUCAAAGUCCUUGCAUACUAUUU	AGGAGGUAUGUGGAUGCUUUGAAA	Translation
gra-miR8776b	Gorai.009G006600	1	24	484	507	UUUCAAAGUCCUUGCAUACUAUUU	AGGAGGUAUGUGGAUGCUUUGAAA	Translation
gra-miR8776c	Gorai.009G006600	1	24	484	507	UUUCAAAGUCCUUGCAUACUAUUU	AGGAGGUAUGUGGAUGCUUUGAAA	Translation
gra-miR8776d	Gorai.009G006600	1	24	484	507	UUUCAAAGUCCUUGCAUACUAUUU	AGGAGGUAUGUGGAUGCUUUGAAA	Translation
gra-miR8777	Gorai.008G049200	1	20	3663	3682	UUUCCAAUAGAAGAAUGACA	UGGUAUUGUUCUAUGGGAGA	Cleavage
gra-miR8777	Gorai.009G059900	1	20	1325	1344	UUUCCAAUAGAAGAAUGACA	UGGCAUUGUUCAAUUGGGGA	Cleavage
gra-miR8777	Gorai.013G214100	1	20	1361	1380	UUUCCAAUAGAAGAAUGACA	UGGCAUUGUUCAAUUGGGGA	Cleavage
gra-miR8777	Gorai.013G149700	1	20	1358	1377	UUUCCAAUAGAAGAAUGACA	UGGCUUUCUUUAAUUGGGGA	Cleavage
gra-miR8777	Gorai.002G103800	1	20	1250	1269	UUUCCAAUAGAAGAAUGACA	UGGCUUUCUUUAAUUGGGGA	Cleavage
gra-miR8778	Gorai.003G115800	1	24	15	38	UUUCCAUAUUAGGGUUUGAACUUU	CGUUAUCAAGGUUAAGUAUGGAAA	Translation
gra-miR8778	Gorai.009G012400	1	24	2676	2699	UUUCCAUAUUAGGGUUUGAACUUU	UUCCCUUGAAGUUCAAUAUGGGAA	Translation
gra-miR8780	Gorai.010G253900	1	24	715	737	UUUGAUGAUGUGGCAACAAAGCGC	CCUUUUUGUU-CCGCGUCAUCAGC	Cleavage
gra-miR8780	Gorai.006G007000	1	24	657	680	UUUGAUGAUGUGGCAACAAAGCGC	UGUUGCAGUUGUUUCAUCAUCAGA	Translation
gra-miR8780	Gorai.010G148800	1	24	853	875	UUUGAUGAUGUGGCAACAAAGCGC	CCCUUUUGUU-CCACCUCAUCAGC	Cleavage
gra-miR8780	Gorai.001G185100	1	24	3203	3226	UUUGAUGAUGUGGCAACAAAGCGC	UACUAUUAUUGUUAUGUCAUGGAA	Cleavage

gra-miR8780	Gorai.004G198100	1	24	334	356	UUUGAUGAUGUGGCAACAAAGCGC	UGCAGUUGUUGCCA-GUCAUUGAG	Translation
gra-miR8780	Gorai.006G206500	1	24	2811	2834	UUUGAUGAUGUGGCAACAAAGCGC	CCUAGAUGUUUCAAUAUCAUCAGG	Cleavage
gra-miR8781a	Gorai.004G101000	1	24	1741	1764	UAUUGAAUUAUUUAGAACUAGGAU	GGUGUGGUCGAAGGUAGUUCAAUA	Cleavage
gra-miR8781b	Gorai.004G109400	1	24	1832	1855	UUUGAUUAGGAAGUUUGAGGAUCA	GCCUGCUUGAGCUUUCUGAUUUGA	Cleavage
gra-miR8781b	Gorai.003G121600	1	24	192	214	UUUGAUUAGGAAGUUUGAGGAUCA	UGA-CUUCGCUCUUUUUGAUCAGA	Cleavage
gra-miR8782	Gorai.009G348900	1	21	2015	2035	UUUGGUGUUGAAGGGGAAUAA	ACAUGCCUCCUCAGUACCAAA	Cleavage
gra-miR8782	Gorai.006G266700	1	21	1015	1035	UUUGGUGUUGAAGGGGAAUAA	AUUGUCACCUUUAAUACCAAG	Cleavage
gra-miR8782	Gorai.001G068000	1	21	747	767	UUUGGUGUUGAAGGGGAAUAA	UUCUUCACCUUCAAUGUCCAA	Cleavage
gra-miR8782	Gorai.005G072800	1	21	1632	1652	UUUGGUGUUGAAGGGGAAUAA	UGAUUUACCUGCAAUGCCAAU	Translation
gra-miR8782	Gorai.005G259100	1	21	2021	2041	UUUGGUGUUGAAGGGGAAUAA	UGAAGCUACUUCAACAUCAAA	Cleavage
gra-miR8782	Gorai.005G259100	1	21	1755	1775	UUUGGUGUUGAAGGGGAAUAA	CGUUCCUUCUUCGACAUUGAA	Cleavage
gra-miR8784	Gorai.003G002500	1	24	521	544	UUUGUCGACAUGUCAGGAAAGCGC	CUGAGUUAUUGAAAUCUCGACAAA	Cleavage
gra-miR8785	Gorai.013G236600	1	21	1816	1836	UUUUACAGCAGCUACAUCCAU	UUGGAAGUGGCUGUUGAGAAA	Cleavage
gra-miR8785	Gorai.001G185100	1	21	913	933	UUUUACAGCAGCUACAUCCAU	CAGUAUGUGGCUGCUGUCAAU	Cleavage
gra-miR8785	Gorai.006G266700	1	21	749	768	UUUUACAGCAGCUACAUCCAU	GUGGAGG-AGCUGCAGUAAAA	Cleavage
gra-miR8785	Gorai.009G006600	1	21	487	507	UUUUACAGCAGCUACAUCCAU	AGGUAUGUGGAUGCUUUGAAA	Translation
gra-miR8785	Gorai.003G120400	1	21	748	768	UUUUACAGCAGCUACAUCCAU	ACGGUUUUAGCAGCUGCAAAA	Translation
gra-miR8786a	Gorai.011G138700	1	24	2101	2124	UUUUAGUGAUGUGGCAGAAAGAUG	AAAUCAUCAGCAGCAUCACUAGAA	Cleavage
gra-miR8786b	Gorai.011G138700	1	24	2101	2124	UUUUAGUGAUGUGGCAGAAAGAUG	AAAUCAUCAGCAGCAUCACUAGAA	Cleavage
gra-miR8787	Gorai.011G138700	1	23	2146	2168	UUUUCUUUUAAUUGGACGAGAUA	UCAGUUGUUAAAUUGGAAGAAGA	Cleavage
gra-miR8787	Gorai.005G035500	1	23	829	851	UUUUCUUUUAAUUGGACGAGAUA	UUUCUCGUGCCAUUAAAGGAACA	Cleavage
gra-miR8787	Gorai.003G115800	1	23	995	1017	UUUUCUUUUAAUUGGACGAGAUA	CCUUUAGUCCAAGUAAAAGGAGC	Translation
gra-miR8787	Gorai.002G110000	1	23	575	597	UUUUCUUUUAAUUGGACGAGAUA	UACCUCGACCAAGUGACAGAAAG	Translation
gra-miR8787	Gorai.002G110000	1	23	3542	3564	UUUUCUUUUAAUUGGACGAGAUA	ACUAUGGUGCAAUUAUAGGAGGA	Cleavage
gra-miR8787	Gorai.013G236600	1	23	775	797	UUUUCUUUUAAUUGGACGAGAUA	AGCCUUGUGCAACUAAGGAAAU	Translation
gra-miR8787	Gorai.005G126000	1	23	2191	2213	UUUUCUUUUAAUUGGACGAGAUA	UCAGUGGUUAAGUUGGAGGAAGA	Cleavage
