# Supplementary material for: Map-Based Functional Analysis of the GhNLP Genes Reveals Their Roles in Enhancing Tolerance to N-Deficiency in Cotton
Source: Int J Mol Sci. 2019 Oct 8;20(19):4953. doi: 10.3390/ijms20194953 (PMC6801916; doi:10.3390/ijms20194953)
Supplement: Supplementary file 1 [file ijms-20-04953-s001.zip › Table S6.pdf]

Table S6: *NLP* gene specific primers for RT-qPCR analysis

S/No.	Seq ID	Orientation	Len	tm	GC%	Any compl	3' compl	Seq
1	Gh_A01G0794	F	20	59.98	50	4	2	AGCGCAGGGAGACATTCTTA
		R	20	60.18	45	6	2	CCACCACATTTGGATGATGA
2	Gh_A03G0454	F	20	59.98	60	4	2	AAGGGGACTAGCTGGAGAGC
		R	20	60.1	45	3	0	TGTGCTATTACCCGCATCAA
3	Gh_A03G0493	F	20	60.05	55	3	2	CAGTGGAGTGCCTGTAGCAA
		R	20	59.87	50	4	0	AGAACCCAAACACGTTGTCC
4	Gh_A03G1178	F	20	60.09	45	5	2	TAGTTACGGCGGAAAAATCG
		R	19	59.66	52.63	3	2	CCAGTGATTCCCGCTGTTA
5	Gh_A03G1567	F	20	59.94	45	4	3	TGGAGTTGTGATTCCACCAA
		R	20	59.95	50	4	0	CTGTGTCATGCCGGATATTG
6	Gh_A05G0079	F	20	60.07	50	7	2	CCATCGAAACCTTCGACCTA
		R	20	60.45	45	4	0	GGCATTTCAAGGAACGAATG
7	Gh_A06G0421	F	20	60.11	45	4	0	CAAGCATTGACGGGTTTCTT
		R	20	59.85	50	5	2	AACCACAGGTTGCCCATAAG
8	Gh_A06G1787	F	20	59.92	45	3	2	CCGAAGTGCCCGATTATTTA
		R	20	59.68	55	4	2	TACTGTCCCGACCGAGAGAT
9	Gh_A06G2074	F	20	59.83	50	5	0	TTTCAGTGACGACCGATGAG
		R	20	59.82	50	4	1	GACATACTGTGGCCGGATTT
10	Gh_A11G0376	F	20	59.88	40	4	2	TCCAAAATGGGAAATGAAGC
		R	20	60.06	45	7	0	AGGATATGCCGCAAATGAAG
11	Gh_A12G0439	F	20	59.96	50	4	2	GAAGCCGTCTGATTTTGAGC
		R	20	59.93	50	4	0	GGCAATGATCCACAAGAGGT
12	Gh_A12G1336	F	21	59.72	52.38	2	0	GCTCCACCCTAACCATAAACC
		R	20	59.23	35	4	1	TGATCTTCGCCAATTCAAAA
13	Gh_D02G1615	F	20	60.09	45	5	2	TAGTTACGGCGGAAAAATCG
		R	20	59.53	50	6	0	GATTCCCGCTGTGAATAAGG
14	Gh_D02G2018	F	20	59.94	45	4	3	TGGAGTTGTGATTCCACCAA
		R	20	59.95	50	4	0	CTGTGTCATGCCGGATATTG
15	Gh_D03G1042	F	20	60.05	55	3	2	CAGTGGAGTGCCTGTAGCAA
		R	20	59.87	50	4	0	AGAACCCAAACACGTTGTCC
16	Gh_D03G1084	F	20	59.98	60	4	2	AAGGGGACTAGCTGGAGAGC
		R	20	60.1	45	3	0	TGTGCTATTACCCGCATCAA
17	Gh_D04G1546	F	20	59.9	45	3	0	TTGGACTTGAAAAGGGGATG
		R	20	60.07	45	6	1	TTGATATCGGCTGGTGTTGA
18	Gh_D05G1588	F	20	59.84	55	4	0	CCTCACCAAGCTCTCCAAAC
		R	20	60.07	50	4	0	GAATTTGCTGTACGGGAGGA
19	Gh_D05G3139	F	20	60.16	45	4	1	TGTTGTTGATCGCCACACTT
		R	20	59.99	50	3	2	TCATCCTGTTGCTTCACTGC
20	Gh_D06G1329	F	20	59.99	45	6	2	TCTTGACGCGTTGATTTCAG
		R	20	59.82	50	4	1	GACATACTGTGGCCGGATTT
21	Gh_D09G0055	F	20	59.93	50	2	0	GTTATCCAAAGCCCTCACCA
		R	20	59.68	50	5	0	GGTTGGGTGTCATCACCTTT
22	Gh_D11G0397	F	20	60.01	50	4	2	AGACACCCGTTTCCAAAGTG
		R	20	59.84	50	4	1	TTCCAGAAGTGCTCCCAACT
23	Gh_D11G0626	F	20	59.84	60	8	3	GCCCCTGAGAAGTCTCACAC
		R	20	60.17	40	4	2	TGAATTTTGTTCCCCGTCAT
24	Gh_D12G0440	F	20	59.96	50	4	2	GAAGCCGTCTGATTTTGAGC
		R	20	59.93	50	4	0	GGCAATGATCCACAAGAGGT
25	Gh_A01G0750	F	20	59.8	60	4	3	GGCTGATGAGAGTGGGTCTC
		R	20	60.1	50	2	0	ATTTGCTCTCTGCTCCTCCA
26	Gh_A01G1468	F	20	59.99	40	3	3	TCAAAACGCTGAAAGCAATG
		R	20	60.04	45	4	2	TTATGGCTTTGGGATCTTGC
27	Gh_A02G0102	F	20	59.86	50	8	1	TGTCATGCGCATAGCTTACC
		R	20	60.04	50	3	1	CCTTTTCAGGAGGACCATCA
28	Gh_A02G0925	F	20	60.11	50	4	1	AAGTTTGATCCAGCCACAGG
		R	20	60.02	50	5	3	TGAACTTGAAGCGGACAGTG
29	Gh_A02G0949	F	20	59.83	50	2	0	TGTCCAAGGTGCTGATGAAG
		R	20	60	55	4	0	CATGGGGTGTTGTCTGAGTG
30	Gh_A05G3286	F	20	59.92	50	3	1	TTCTCTCTTCCCCTTGACGA
		R	20	60.36	45	7	2	AAGCAGCAATGATGCCTTTC
31	Gh_A05G3990	F	20	59.97	50	3	2	GATGACCAGGCAAAACAGGT
		R	20	60.07	40	5	2	TGCAAGTATGCCATTCCAAA
32	Gh_A08G0768	F	20	59.96	45	5	2	TTAATGCCACTGGTGTTCCA
		R	20	60.05	45	4	0	TTTGGACGGGGATATGTTGT
33	Gh_A08G0810	F	20	59.72	40	5	1	AAAAGTTGCGGAAACCAGAA
		R	20	60.03	55	3	2	CTGCTCTGCTGCTTCATCTG
34	Gh_A09G1689	F	20	59.96	55	5	2	GCAGGGAGTCTCAAAGATGC
		R	20	60.13	55	4	1	AACTTCCGGCACCCTCTACT
35	Gh_A10G1257	F	20	59.91	55	3	0	GGGTTCTTTCCCTGGTCTTC
		R	20	60.14	50	4	3	AACCGCAGCATTCAGGTTAC
36	Gh_A13G2318	F	20	60.11	45	6	2	AGTTCGCCCAATTGTTCAAG
		R	20	59.83	45	6	2	CATGATCGAGCGTTCGTAAA
37	Gh_D01G0769	F	20	59.96	50	4	2	GTGAGCAAGCGATTTCCTTC
		R	20	60.03	50	7	1	GCCACTTGAAGTTGTGCTGA
38	Gh_D01G1705	F	20	60.12	50	7	0	TTATGCAGCCTGCTCCTTCT
		R	20	60.18	50	6	2	TTGCAGACGTGCTCAAGTTC
39	Gh_D02G0126	F	20	60.15	50	5	2	AACCTTCCCGTCGGAATATC

		R	20	59.83	45	4	0	CAGTTGGAATGTCCCGTTTT
40	Gh_D02G1107	F	20	59.88	45	6	3	GCATTTGTTGCAGCAGGTAA
		R	20	60.02	50	5	3	TGAACTTGAAGCGGACAGTG
41	Gh_D03G0017	F	20	59.95	55	6	2	TCTATTGCAGGGGGTACCAG
		R	20	59.84	45	3	0	ACATCATTCGCCTCTTTGCT
42	Gh_D03G0813	F	20	59.83	50	2	0	TGTCCAAGGTGCTGATGAAG
		R	20	60	55	4	0	CATGGGGTGTTGTCTGAGTG
43	Gh_D05G0100	F	20	59.99	55	4	2	CTGGGCATGGTTGTAGTCCT
		R	20	59.96	55	4	0	GACTTGCCATGCTCTCCTTC
44	Gh_D05G1709	F	20	59.99	50	5	3	GCACTGCCATGAAACTCAGA
		R	20	60.26	55	4	1	CAGCTCACCATTCCCAGAGT
45	Gh_D08G0987	F	20	59.69	45	5	3	CATGCCAGAAAAGTTGTGGA
		R	20	60.03	55	3	3	CTCTGCTGCTTCATCTGCTG
46	Gh_D08G1195	F	20	60.01	50	5	3	GCCTTGGTGTTTGTCCAACT
		R	20	60.01	55	3	2	ACTTCCAGCAGCCTCCACTA
47	Gh_D08G1828	F	20	60.46	45	4	2	TGGCAGATCCAAGTTCCAAT
		R	20	60.07	50	4	2	CTGTTCCCTGCTTGATCCAT
48	Gh_D09G1795	F	20	59.96	55	5	2	GCAGGGAGTCTCAAAGATGC
		R	20	60.13	55	4	1	AACTTCCGGCACCCTCTACT
49	Gh_D10G1228	F	20	59.96	50	4	2	CCGTTGGCTCTAACATGGAT
		R	20	60.12	50	7	1	GCTTTCGTGGTCCACTCATT
50	Gh_D12G0368	F	20	59.73	45	6	0	TTGCGGACGTCACATTTTAG
		R	20	59.98	45	5	0	ACTTTTTGGCAAGGGAGGTT
51	Gh_D13G1358	F	20	59.99	50	4	1	AGCCTTCGAGTGGTTCTTCA
		R	20	59.83	50	5	2	CTGAACAGTGCTCCATTCCA
52	Gh_D13G1958	F	20	60.23	45	4	1	CGAAATTGCTGCGGATAAGT
		R	20	59.86	50	4	1	ATGCAGTCACATTGGAGCAG
53	Gh_D13G2470	F	20	60.1	55	2	2	CGGGTGAAGAAGAGGGTGTA
		R	20	59.99	50	4	0	CTCTGCCACTTTTTGGCTTC
